# Genome-wide identification and expression analysis of the NAC transcription factor family in *Saccharum spontaneum* under different stresses

**DOI:** 10.1080/15592324.2022.2088665

**Published:** 2022-06-22

**Authors:** Qingqing Shen, Zhenfeng Qian, Tianju Wang, Xueting Zhao, Shujie Gu, Xibing Rao, Shaozhi Lyu, Rongqiong Zhang, Lilian He, Fusheng Li

**Affiliations:** a Sugarcane Research Institute, Yunnan Agricultural University, Kunming, Yunnan, China; b College of Agronomy and Biotechnology, Yunnan Agricultural University, Kunming, Yunnan, China; c Institute for Bio-resources Research and Development of Central Yunnan Plateau, Chuxiong Normal University, Chuxiong, China; d Key Laboratory for Crop Production and Smart Agriculture of Yunnan Province, Yunnan Agricultural University, Kunming, Yunnan, China

**Keywords:** Sugarcane, NAC gene, transcription factor, stress

## Abstract

The *NAC* (*NAM, ATAF1/2*, and *CUC2*) transcription factor family is one of the largest families unique to plants and is involved in plant growth and development, organs, morphogenesis, and stress responses. The *NAC* family has been identified in many plants. As the main source of resistance genes for sugarcane breeding, the *NAC* gene family in the wild species *Saccharum spontaneum* has not been systematically studied. In this study, 115 *SsNAC* genes were identified in the *S. spontaneum* genome, and these genes were heterogeneously distributed on 25 chromosomes. Phylogenetic analysis divided the *SsNAC* family members into 18 subgroups, and the gene structure and conserved motif analysis further supported the phylogenetic classification. Four groups of tandemly duplicated genes and nine pairs of segmentally duplicated genes were detected. The *SsNAC* gene has different expression patterns at different developmental stages of stems and leaves. Further qRT–PCR analysis showed that drought, low-temperature, salinity, pathogenic fungi, and other stresses as well as abscisic acid (ABA) and methyl jasmonate (MeJA) treatments significantly induced the expression of 12 *SsNAC* genes, indicating that these genes may play a key role in the resistance of *S. spontaneum* to biotic and abiotic stresses. In summary, the results from this study provide comprehensive information on the *NAC* transcription factor family, providing a reference for further functional studies of the *SsNAC* gene.

## Introduction

The *NAC* family is one of the largest transcription factor families unique to plants, and its name derives from the initials of the *NAM* of Petunia × atkinsiana, *Arabidopsis thaliana* activating factor 1 (*ATAF1), ATAF2*, and *CUC2* genes.^1^ *NAC* family members have significant structural characteristics. The N-terminus contains a highly conserved *NAC* domain consisting of approximately 150 amino acid residues.^2^ This domain contains five subdomains (A-E), including conserved subdomains A, C, and D and diversified subdomains B and E; the C-terminus is the transcriptional activation domain (TAR), which is less conserved and has a high degree of diversity.^3,4^

*NAC* transcription factors play important roles in the regulation of plant growth and development,^4,5^ morphogenesis,^6,7^ leaf senescence,^8,9^ and tolerance to biotic and abiotic stresses.^10,11^ Studies have confirmed that many *NAC* genes play key roles in the regulation of drought tolerance, salt tolerance, low-temperature resistance, high-temperature resistance, and pathogen resistance in plants. Overexpression of maize (*Zea mays*) *ZmNAC33* and *ZmNAC77* in *A. thaliana* can improve the drought tolerance of transgenic *A. thaliana*.^12,13^ In rice (*Oryza sativa), OsNAC041* mutants exhibited higher salt sensitivity,^14^ and *OsNAC2* overexpression inhibited the salt tolerance of transgenic *O. sativa*.^15^ The ectopic expression of the *CmNAC1* gene in *Cucurbita moschat*a enhances the tolerance of transgenic *A. thaliana* to cold, salt, and drought stresses.^16^ *VvNAC17* gene overexpression in grape (*Vitis vinifera L*.) increased tolerance and the survival rate under freezing stress, increased the survival rate, and decreased the water loss rate under drought and dehydration stresses.^17^ Overexpression of the drought tolerance gene *HvNAC1* in barley (*Hordeum vulgare*) reduced the occurrence of Ramularia leaf spot symptoms and the colonization of *R. collo-cygni* fungi.^18^ Furthermore, overexpression of *Artemisia annua AaNAC1* in *A. thaliana* enhanced resistance to Botrytis cinerea and drought tolerance.^19^

Since high-throughput sequencing technology was introduced in genomics research in 2005, an increasing number of plant genes have been sequenced, providing a platform for the analysis of gene families at the whole-genome level.^20^ In recent years, *NAC* transcription factor families have been identified and analyzed in many species. Rice and *A. thaliana* contains 151 and 117 *NAC* genes, respectively.^21,22^ Based on the *Z. mays* genome sequence, 148 and 152 *Z. mays NAC* members have been identified, respectively.^23,24^ A total of 131 *SbNAC* genes have been identified in the sorghum genome,^25^ 108 *DgNAC* genes have been identified in the *Dactylis glomerata* genome,^26^ and 132 *AhNAC* and 104 *CaNAC* genes have been identified in the cultivated peanut (*Arachis hypogaea*) and pepper (*Capsicum annuum*) genomes, respectively.^27,28^ *Saccharum spontaneum* L. is a wild relative of sugarcane. It is mainly distributed in tropical and subtropical regions between 8°S and 40°S. Because of its wide distribution, it can adapt to multiple adversities in nature and has been used for sugarcane crossbreeding since the 19th century. Currently, the vast majority of modern sugarcane cultivars contain *S. spontaneum*, the most important resource material for sugarcane resistance breeding.^29^ However, no comprehensive systemic study of the *NAC* gene family in *S. spontaneum* has been conducted. In this study, we identified *NAC* gene family members in the *S. spontaneum* genome, constructed a phylogenetic tree for classification, further analyzed the characteristics of the gene structure and conserved motifs, and comprehensively analyzed gene replication events and collinearity. In addition, the tissue specificity of the *SsNAC* gene in the growth and development of *S. spontaneum* was analyzed. Finally, qRT–PCR was used to identify the differential expression patterns of 12 *SsNAC* genes under biotic and abiotic stresses and hormone induction. In summary, the results from this study provide comprehensive information on *NAC* genes in *S. spontaneum*, laying a foundation for in-depth studies of *NAC* gene function in *S. spontaneum*, and are expected to provide a source of candidate genes for molecular resistance breeding of sugarcane.

## Materials and methods

### *Identification of NAC genes in* S. spontaneum

The *S. spontaneum* genome was downloaded from the *S. spontaneum* AP85–441 genome database (http://www.life.illinois.edu/ming/downloads/Spontaneum_genome/),^30^ the *Arabidopsis thaliana NAC* (*AtNAC*) family data were downloaded from The Arabidopsis Information Resource (TAIR) (www.Arabidopsis.org),^31^ and the information for genes in the *OsNAC* family were downloaded from GenBank (http://www.ncbi.nlm.nih.gov).^32^ Hidden Markov model (HMM) files for the *NAC* domain (PF01849) and the *NAM* domain (PF02365) were downloaded from Pfam (http://pfam.xfam.org/), and the hmmsearch command was used to query the *NAC* and *NAM* conserved domains in the genomic sequence of *S. spontaneum*. Duplicated *SsNAC* gene transcripts were removed. The conserved domain database (CDD, http://www.ncbi.nlm.nih.gov/cdd/) and the PFAM database (https://pfam.xfam.org) were used to confirm the obtained *SsNAC* genes, and sequences with the NAC or NAM domain were regarded as *SsNAC* genes and used in subsequent analyses.

### Construction of the phylogenetic tree

The conserved domains of 115 *SsNAC*, 105 *AtNAC*, and seven *OsNAC* genes were used for evolutionary and phylogenetic analyses of the *NAC* gene family, and unrooted phylogenetic trees were generated using the neighbor-joining method in MEGA-X (https://www.megasoftware.net/). This method includes 1000 guided replications and paired detection.^33^ *SsNAC* classification was determined based on the classification of the *AtNAC* and *OsNAC* gene families.^22^

### Analysis of protein characteristics, gene structure, and gene motifs

ExPASy (http://web.expasy.org/protparam/) was used to predict the physical and chemical characteristics of the *SsNAC* proteins. The subcellular localization of *SsNAC* protein was predicted using WoLF PSORT (https://wolfpsort.hgc.jp/). The structures of the *SsNAC* genes were determined using GSDS 2.0.^34^ The default parameters were set using MEME tools (http://meme-suite.org/tools/meme) to identify the conserved motifs in the *SsNAC* protein, and the maximum number of conserved motifs was set to 10.^35^

### Chromosome localization, gene duplication, and collinearity analysis

Using the *S. spontaneum* genome annotations, Map Gene 2 Chrome (http://mg2c.iask.in/mg2c_v2.0/) was used to map *SsNAC* genes and conduct chromosome matching. Default parameters in MCScanX were used to detect *SsNAC* duplicate genes and collinear blocks.^36^

### Plant material and stress treatment

*S. spontaneum* clones were used as test materials. Drought treatment was applied as follows. Watering in the elongation period (5 months old) was stopped when the soil water content reached 17–20% (0 d), 12.5–15% (2 d), 10–12.5% (4 d), and 7.5–10% (6 d), which were defined as adequate water, mild drought, moderate drought, and severe drought, respectively^37^ and +1 leaves were collected at these time points. Salt treatment was applied as follows. One liter of 150 mM NaCl was used to irrigate the seedlings (1-month-old). Low-temperature treatment was applied as follows. Clones at the 1-month-old of the seedling stage were placed in an incubator at 4°C, and +1 leaves were collected at 0 h, 3 h, 6 h, 12 h, and 24 h after the above treatment. *Fusarium verticillioides* treatment was applied as follows. An *F. verticillioides* spore suspension (1 × 10^6^ cells/mL) was inoculated into seedlings during the 1-month-old stage using a 1-mL syringe, and the leaf sheaths at the lower part of the inoculation site were collected at 0 h, 12 h, 24 h, and 48 h after inoculation. Hormone induction was conducted as follows. The leaves of 1-month-old seedlings were sprayed with 100 μM abscisic acid (ABA) and 100 μM methyl jasmonate (MeJA) until droplets were observed, and +1 leaves were collected at 0 h, 3 h, 6 h, 12 h, and 24 h later. Three independent biological replicates were set up for all treatments. The treated samples were immediately frozen in liquid nitrogen and stored at −80°C for subsequent analysis.

To investigate the expression patterns of *SsNAC* genes under different stresses and hormone treatments, we selected 12 *SsNAC* genes differentially expressed under drought stress according to RNA-seq data (Table S6) for further qRT–PCR analysis. Differentially expressed genes (DEGs) were defined by a log2|fold change| > 1 and a false discovery rate (FDR) < 0.05.

### *The expression patterns of* SsNAC *genes under different stress and hormone treatments*

Total RNA was extracted using an OMEGA Plant RNA Kit (R6827-01) following the manufacturer’s protocol. Agarose gel electrophoresis was used to detect RNA integrity. RNA quality and quantification were assessed using a Thermo Fisher NanoDrop™ One/One c Microvolume UV–visible spectrophotometer. First-strand cDNA was synthesized from DNA-free RNA using a Star Script II First-strand cDNA Synthesis Kit with gDNA Remover (Gen Star) following the manufacturer’s protocol. Real-time fluorescence quantitative PCR was performed using an Applied Biosystems ABI 7500 instrument and SYBR Green, and GAPDH gene expression was used as an internal control. Primer 5.0 was used to design the primers (Table S1). The PCR system and procedures were performed in accordance with the 2 × Real Star Green Fast Mixture (with ROX II) (Gen Star) kit. Each reaction was performed three times, and the relative gene expression level was calculated using the 2^−ΔΔCT^ method. Significance (P < 0.05) was calculated using the T-test.

## Results

### *Identification and phylogenetic analysis of* SsNAC *genes*

After searching using the HMM of the *NAC* domain and *NAM* domain and screening the CDD database and PFAM database, 115 *SsNAC* genes were identified in *S. spontaneum*. All *SsNAC* proteins have an *NAC* or *NAM* domain. The 115 *SsNAC* genes were named *SsNAC* 001 to *SsNAC* 115 according to the chromosome numbering of the *S. spontaneum* genome (Table S2).

**Figure 1.**
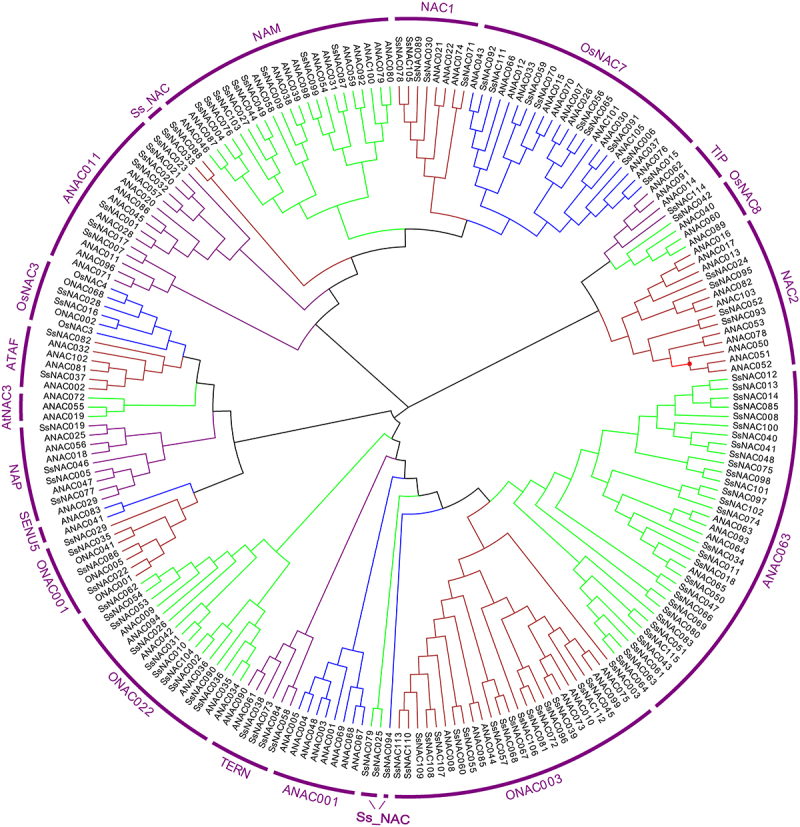
Phylogenetic analysis of NAC proteins in *S. spontaneum, O. sativa*, and *A. thaliana*. Clustal W was used to align the protein sequences, and phylogenetic trees were generated using the neighbor-joining method in MEGA-X; bootstrapping was repeated 1000 times.

Phylogenetic analysis of NAC proteins in *S. spontaneum, O. sativa*, and *A. thaliana* was used to evaluate the evolutionary relationships among SsNAC proteins. Based on the branching and classification of the *NAC* gene family in *O. sativa* and *A. thaliana*, SsNAC proteins were divided into 18 subgroups. The ANAC063 subgroup contained the largest number of SsNAC proteins (30 proteins). The TIP subgroup contained only one SsNAC protein, and the other five SsNAC proteins were divided into specific subgroups and denoted *Ss_NAC*. No member of the SENU5 subgroup was identified in the *SsNAC* family (Figure 1).

### Analysis of the characteristics and structure of SsNAC proteins

The analysis of the physicochemical properties of SsNAC proteins indicated that the length ranged from 159 to 3849 amino acids (aa), the molecular weight (MW) ranged from 17940.33 to 425609.23 Da, and the theoretical isoelectric point (pI) ranged from 4.56 to 10.31, with an average of 6.89. Seventy-one SsNAC proteins were acidic (pI < 7), and the predicted aliphatic amino acid indices ranged from 47.2 to 88.16. In the *SsNAC* family, 24 proteins were stable (instability index (II) < 40), and the rest were unstable. Fifty-six proteins were amphiphilic (grand average of hydropathicity index (GRAVY) = −0.5 to +0.5), and the remaining 59 SsNAC proteins were hydrophilic. The prediction of subcellular localization suggested that all SsNAC proteins were located in the nuclear region (Table S3).

Gene structure analysis (Figure S1B) indicated that the distribution of introns in *SsNAC* genes was diverse and that the number of introns ranged from 0 to 51, mainly with one to three introns. Forty-one *SsNAC* genes contained two introns, accounting for 35.6% of the genes identified, and nine *SsNAC* genes contained more than 10 introns; eight *SsNAC* genes had no introns. Therefore, the structure of *SsNAC* genes is very diverse.

Conserved motifs were used to analyze the structural characteristics of SsNAC proteins. As shown in Figure S1C, a total of 10 conserved motifs (motifs 1–10) were identified in all the SsNAC protein sequences using MEME, where motifs 2, 5, 9, 6, and 7 represent subdomains A, B, C, D, and E, respectively (Table S4). Most of the SsNAC proteins contained seven conserved motifs (motifs 1, 2, 3, 5, 6, 7, and 9), and the SsNAC proteins in the same group had similar motif compositions and positions. These results indicate that *NAC* family members in the same branch may have similar biological functions.

In summary, *SsNAC* family members in the same group on the phylogenetic tree have similar exon–intron gene structures and conserved motifs, further supporting the reliability of the classification.

### SsNAC *gene chromosome localization, gene replication, and collinearity analysis*

**Figure 2.**
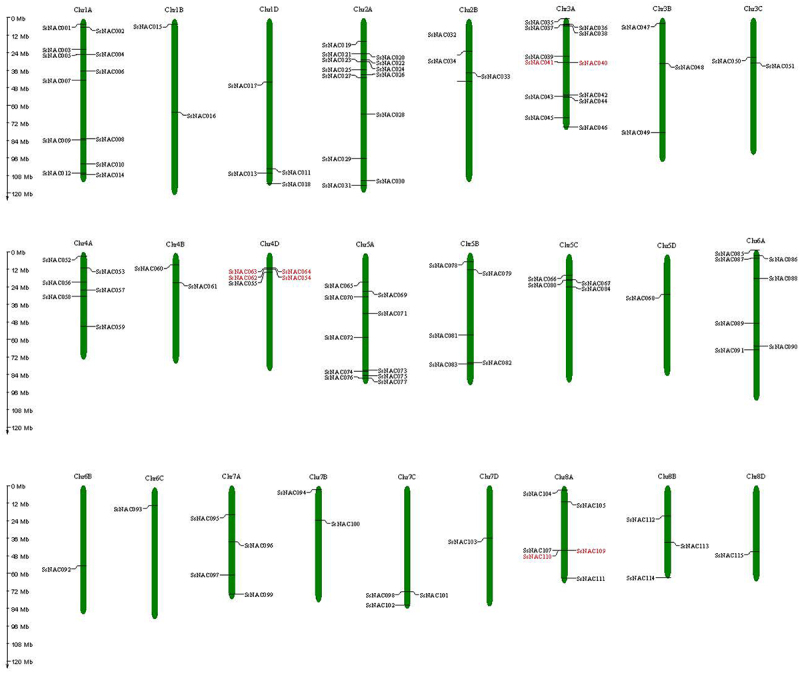
The chromosome locations of *SsNAC* gene family members. Red indicates tandem-duplicated genes.

All *SsNAC* genes were unevenly distributed among the 25 chromosomes of *S. spontaneum*, and chromosome 2 (Chr2A) contained the largest number of *SsNAC* genes (13 genes), followed by chromosome 1 (Chr1A, 12 genes), chromosome 3 (Chr3A, 12 genes), and chromosome 5 (Chr5A, 10 genes) (Figure 2).

**Figure 3.**
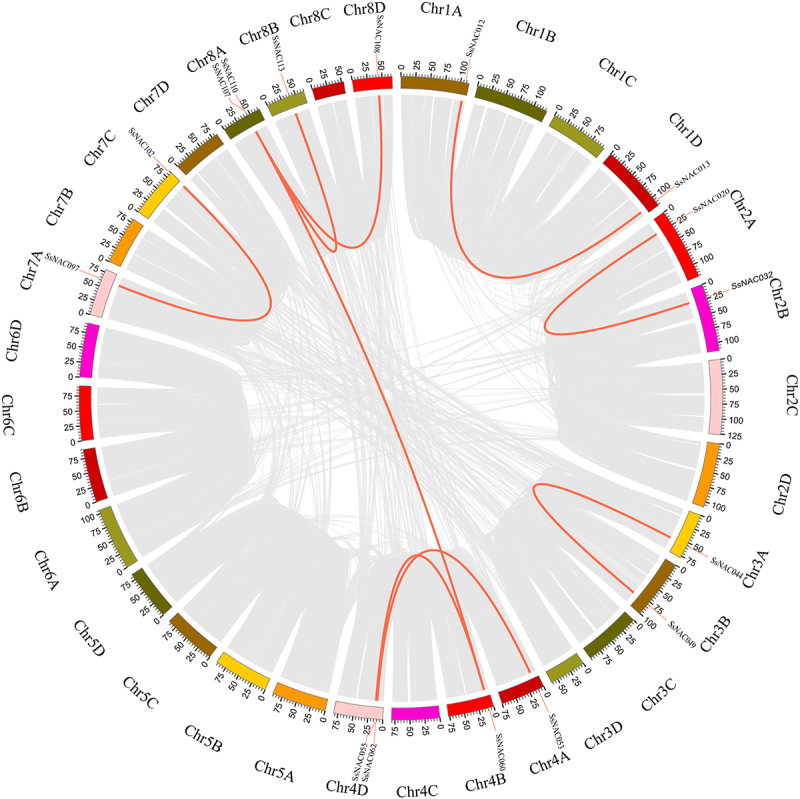
Gene duplication events in the *SsNAC* gene family. The gray line represents the collinear region in the *S. spontaneum* genome, and the red line represents duplicated *SsNAC* gene pairs.

Collinearity analysis was performed using MCScanX software to identify duplicate *SsNAC* events. Nine pairs of segmental-duplicated genes and four groups of tandem-duplicated genes were identified (Figure 2; Figure 3). Some *SsNAC* genes were generated by gene duplication, and segmental duplication events promoted the evolution of the *NAC* family.

**Figure 4.**
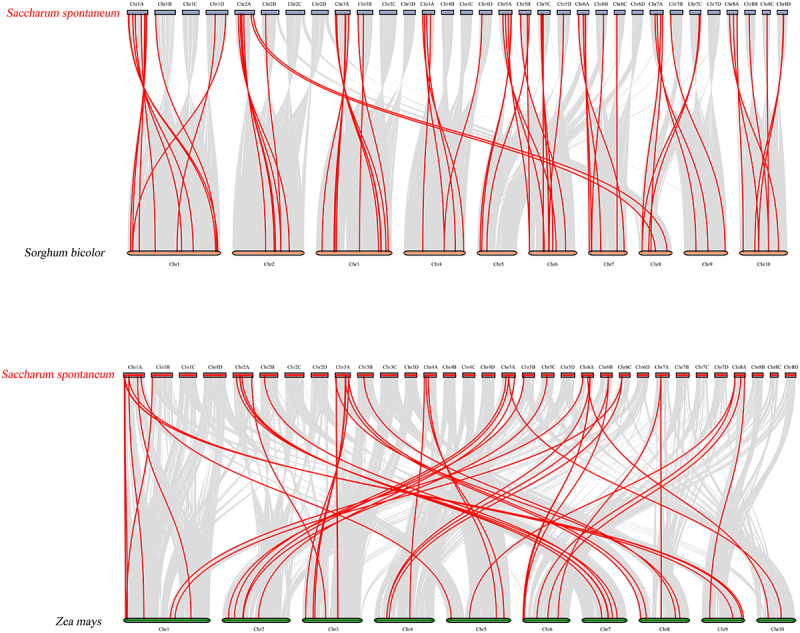
Collinearity analysis among *S. spontaneum, S. bicolor*, and *Z. mays*. The gray line represents collinear regions within the genome, and the red line represents collinear *NAC* gene pairs.

To further infer the phylogenetic mechanisms of *SsNAC* family members, we constructed a comparative alignment diagram of *S. spontaneum* with *Sorghum bicolor* and *Zea mays*. The *Ss*NAC gene is highly homologous to the *NAC* genes of *S. bicolor* and *Z. mays* and exhibits collinearity and conservation. In *S. bicolor*, 69 homologous gene pairs were distributed on all chromosomes, and 49 homologous gene pairs in *Z. mays* were distributed on all chromosomes, 34 of which were shared by *S. spontaneum, S. bicolor*, and *Z. mays* (Figure 4).

### *The expression patterns of* SsNAC *genes during the growth and development of different tissues*

**Figure 5.**
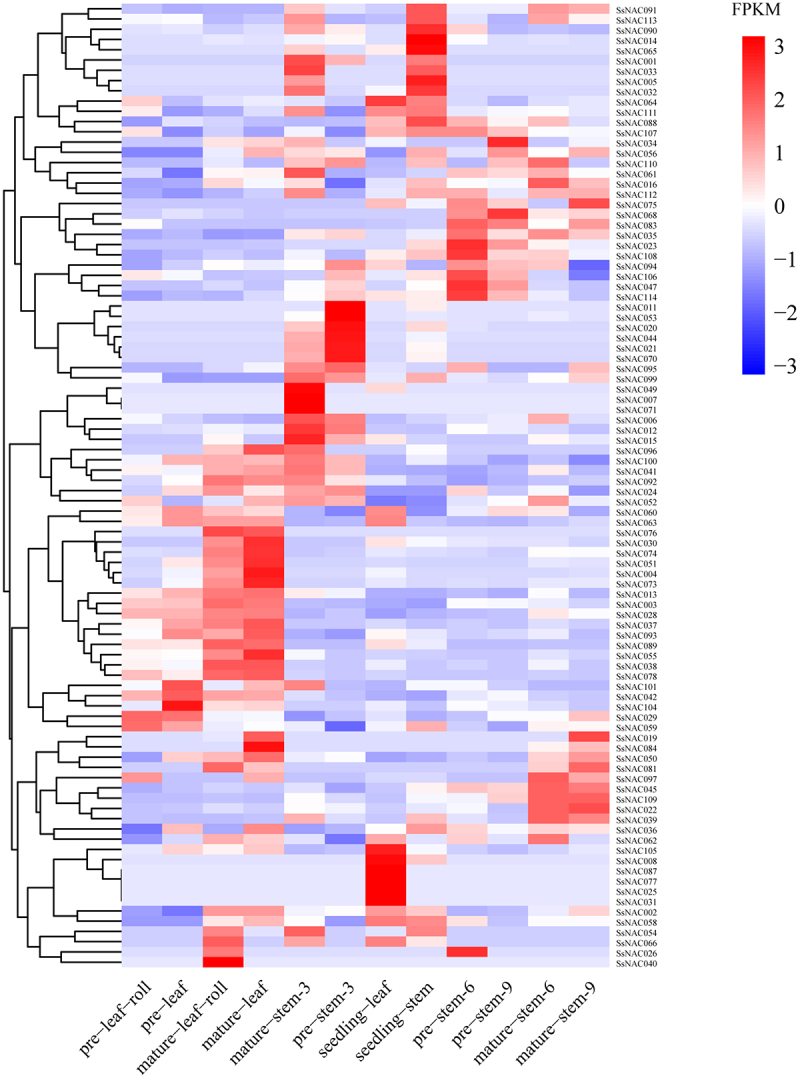
The expression patterns of *SsNAC* genes in different tissues during the growth and development of *S. spontaneum*. Pre-leaf-roll represents immature leaf rolls, pre-leaf represents immature leaves, mature-leaf-roll represents mature leaf rolls, mature-leaf represents mature leaves, mature-stem-3 represents the third segment of mature stems, pre-stem-3 represents the third segment of immature stems, seedling-leaf represents seedling leaves, seedling-stem represents the seedling stem segment, pre-stem-6 represents the sixth segment of immature stems, pre-stem-9 represents the ninth segment of immature stems, mature-stem-6 represents the sixth segment of mature stems, and mature-stem-9 represents the ninth segment of mature stems. Hierarchical cluster analysis was performed using FPKM values to generate heatmaps. The scale represents the relative signal intensity of the FPKM value.

The RNA-seq data in the SGD database (http://sugarcane.zhangjisenlab.cn/sgd/html/mRNA.html) were used to analyze the expression patterns of *SsNAC* genes at different developmental stages of stems and leaves of *S. spontaneum* (Table S5). Thirty-six *SsNAC* genes were commonly expressed during the development of stems and leaves of *S. spontaneum. SsNAC094* expression was high, suggesting that this gene plays an important role in the growth and development of *S. spontaneum*. Fifty-eight *SsNAC* genes were highly expressed during certain developmental periods of the stem and leaves, with low or no expression during some developmental periods. In addition, 21 *SsNAC* genes were not expressed at all stages of stem and leaf development (Figure 5), indicating that *SsNAC* gene expression has tissue and time specificity.

### *The expression patterns of* SsNAC *genes*

**Figure 6.**
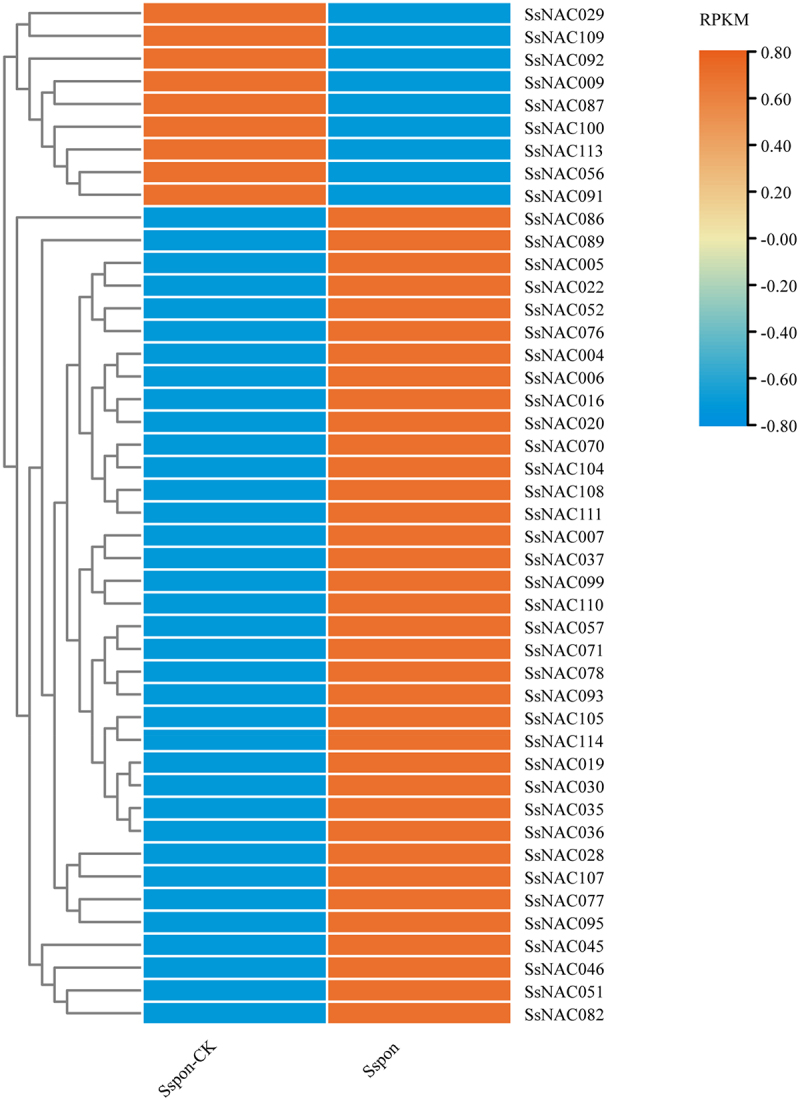
Expression patterns of *SsNAC* genes at the transcriptional level in response to drought stress. A heat map shows the relative expression of *SsNAC* genes at 0 d (Sspon-CK) and 7 d (Sspon) of drought stress. Analysis of RNA-seq data showed 45 differentially expressed *SsNAC* genes that exhibited either increased or decreased expression under drought stress. Red indicates a high expression level, and blue indicates a low expression level.

To analyze the response patterns of *SsNAC* genes under different biotic and abiotic stresses and the transcriptional expression characteristics of *SsNAC* genes under hormone induction, we selected *SsNAC005, SsNAC016, SsNAC020, SsNAC028, SsNAC030, SsNAC037, SsNAC052, SsNAC077, SsNAC095, SsNAC107, SsNAC110*, and *SsNAC111* to conduct qRT–PCR (Figure 6).

**Figure 7.**
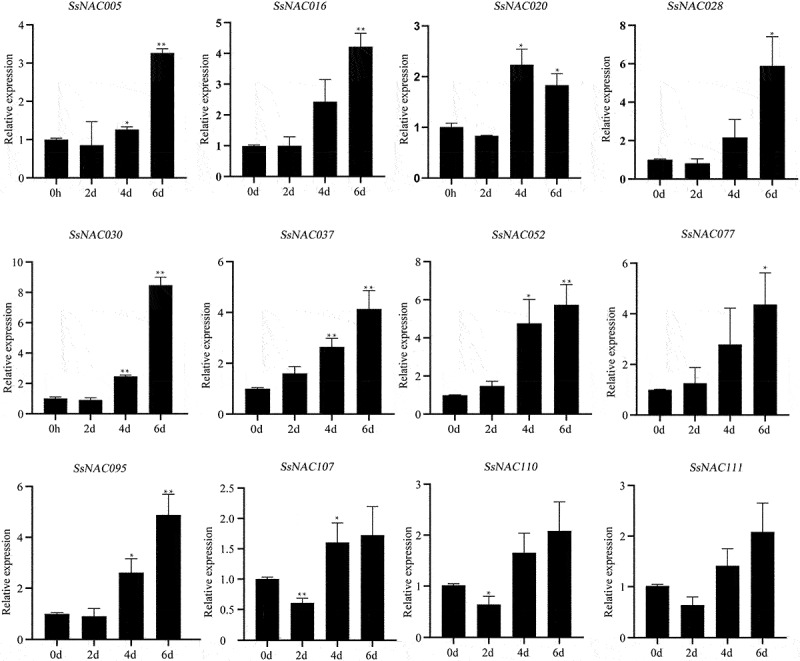
Expression profiles of *SsNAC* genes in *S. spontaneum* under drought treatment. Data represent the mean (± standard deviation (SD)) of three independent replicates. The bars represent the standard error of the mean. Asterisks indicate that the expression of the target gene was significantly upregulated or downregulated under different treatments (* P < 0.05, ** P < 0.01, Student’s t-test).

Under drought treatment, the expression levels of 12 *SsNAC* genes were upregulated with prolonged drought treatment. The *SsNAC005, SsNAC016, SsNAC028, SsNAC030, SsNAC037, SsNAC052, SsNAC077*, and *SsNAC095* genes reached their peak expression levels at 6 d, and these levels were 3.26, 4.21, 5.88, 8.47, 4.13, 5.73, 4.36, and 4.88 times higher than those in the control condition (0 d), respectively (Figure 7), indicating that these *SsNAC* genes exhibit drought stress-induced upregulation.

**Figure 8.**
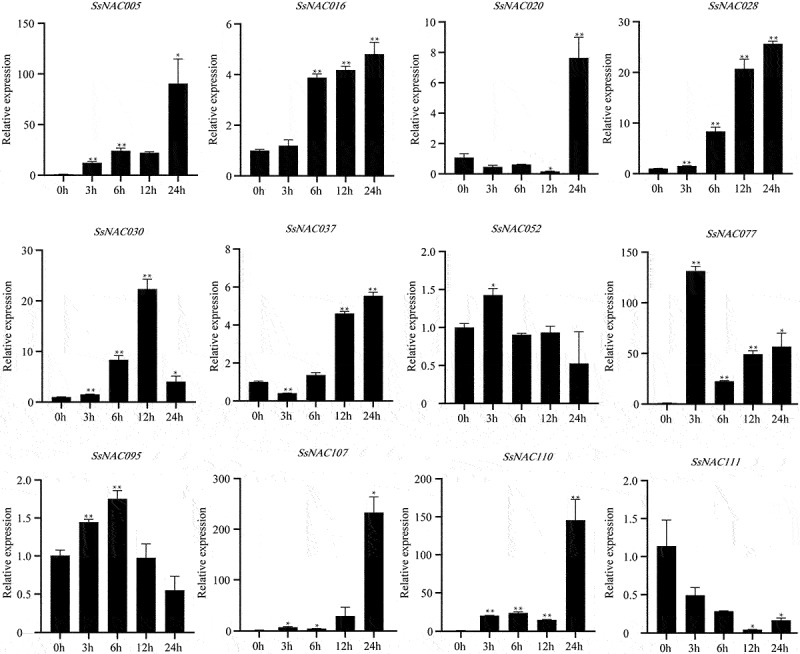
Expression profiles of *SsNAC* genes in *S. spontaneum* under cold treatment. Data represent the mean (± standard deviation (SD)) of three independent replicates. The bars represent the standard error of the mean. Asterisks indicate that the expression of the target gene was significantly upregulated or downregulated under different treatments (* P < 0.05, ** P < 0.01, Student’s t-test).

As shown in Figure 8, *SsNAC005, SsNAC028, SsNAC030, SsNAC052, SsNAC077, SsNAC095, SsNAC107*, and *SsNAC110* gene expression levels were rapidly upregulated at the early stage of low-temperature treatment (3 h). Additionally, the expression levels were significantly higher than those in the control condition (0 h) at the low-temperature treatment stage. In particular, at 3 h, the *SsNAC077* gene expression level was 131.41 times higher than that at 0 h. Seven genes reached the highest expression level in the late stage of low-temperature treatment (24 h), showing significant or highly significant differences relative to the control (0 h). These *SsNAC* genes are more sensitive to low-temperature stress, and it is speculated that they may be specific factors in the signaling pathway of the low-temperature stress response in *S. spontaneum*.

**Figure 9.**
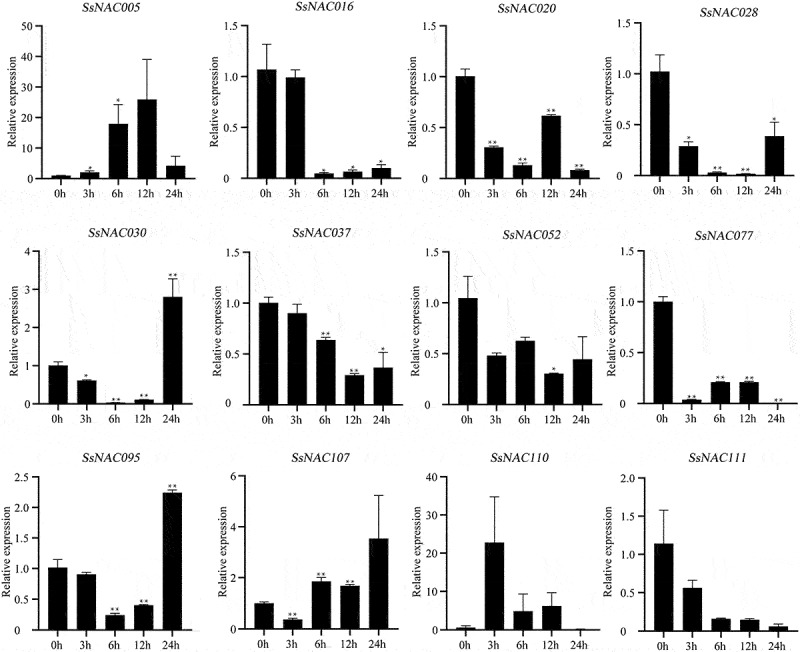
Expression profiles of *SsNAC* genes in *S. spontaneum* under salt treatment. Data represent the mean (± standard deviation (SD)) of three independent replicates. The bars represent the standard error of the mean. Asterisks indicate that the expression of the target gene was significantly upregulated or downregulated under different treatments (* P < 0.05, ** P < 0.01, Student’s t-test).

The expression levels of the *SsNAC016, SsNAC020, SsNAC028, SsNAC030, SsNAC037, SsNAC077*, and *SsNAC095* genes decreased between 3 h and 6 h in the early stage of salt stress. However, only expression of the *SsNAC030* and *SsNAC095* genes increased again in the late stage of salt stress (Figure 9). After salt treatment, some *SsNAC* genes showed a consistent expression response, with low expression at 24 h compared to the control (0 h).

**Figure 10.**
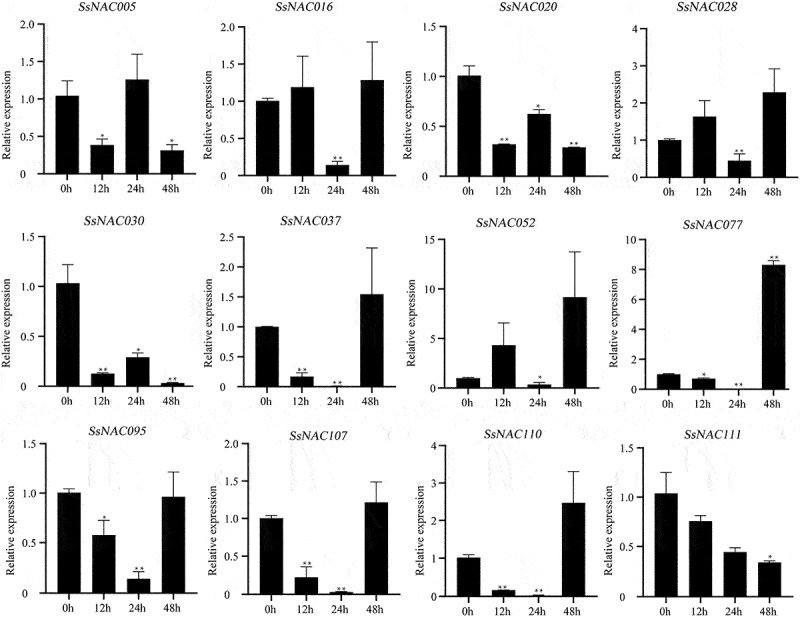
Expression profiles of *SsNAC* genes in *S. spontaneum* under pathogenic fungi treatment. Data represent the mean (± standard deviation (SD)) of three independent replicates. The bars represent the standard error of the mean. Asterisks indicate that the expression of the target gene was significantly upregulated or downregulated under different treatments (* P < 0.05, ** P < 0.01, Student’s t-test).

Most of the *SsNAC* gene expression levels were rapidly downregulated at the early stage of pathogenic fungi treatment (12 h). After 24 h of infection with a pathogenic fungus, some *SsNAC* genes (*SsNAC005, SsNAC020*, and *SsNAC030*) exhibited increased expression followed by a decrease in expression. However, other *SsNAC* genes (*SsNAC037, SsNAC077, SsNAC095, SsNAC107*, and *SsNAC110*) trended toward decreased expression followed by an increase in expression (Figure 10). Therefore, *SsNAC* genes showed two different patterns in response to pathogenic fungal stress.

**Figure 11.**
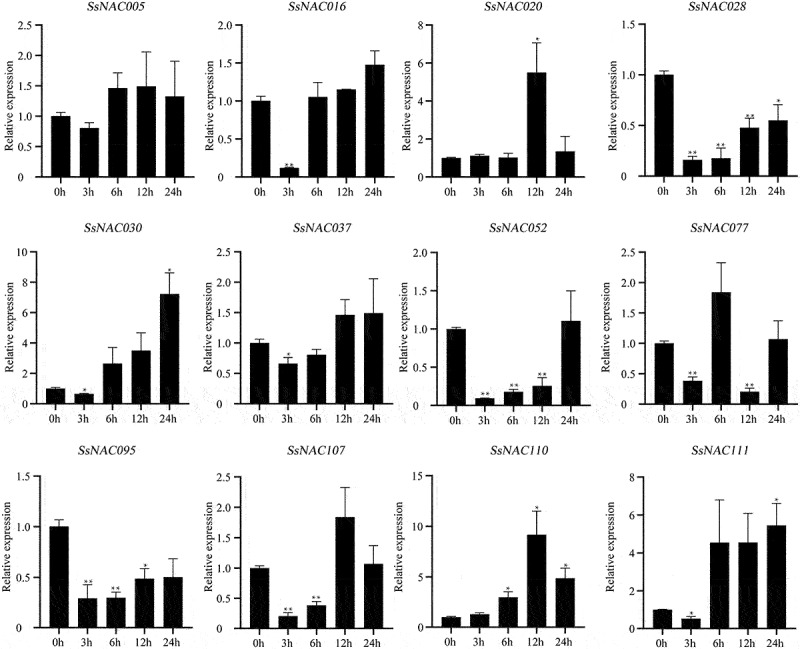
Expression profiles of *SsNAC* genes in *S. spontaneum* under ABA treatment. Data represent the mean (± standard deviation (SD)) of three independent replicates. The bars represent the standard error of the mean. Asterisks indicate that the expression of the target gene was significantly upregulated or downregulated under different treatments (* P < 0.05, ** P < 0.01, Student’s t-test).

**Figure 12.**
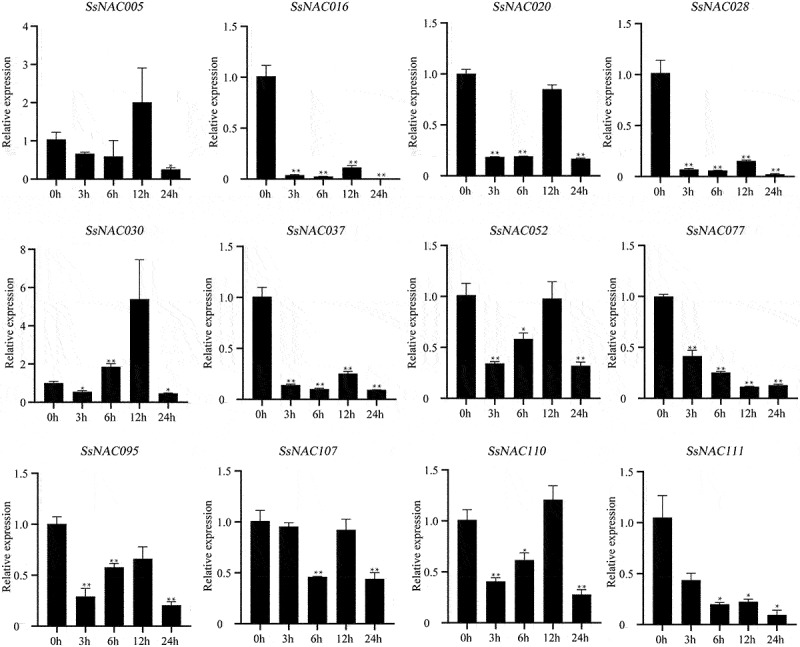
Expression profiles of *SsNAC* genes in *S. spontaneum* under MeJA treatment. Data represent the mean (± standard deviation (SD)) of three independent replicates. The bars represent the standard error of the mean. Asterisks indicate that the expression of the target gene was significantly upregulated or downregulated under different treatments (* P < 0.05, ** P < 0.01, Student’s t-test).

Most *SsNAC* genes (*SsNAC016, SsNAC020, SsNAC030, SsNAC037, SsNAC107, SsNAC110*, and *SsNAC111*) exhibited increased expression between 3 h and 12 h after induction by exogenous ABA, with peak expression levels at 12 h or 24 h (Figure 11). However, after exogenous MeJA induction, only the *SsNAC03* and *SsNAC110* genes showed the same expression pattern as after ABA induction, and the expression levels of the remaining *SsNAC* genes were lower than those of the control at all stages (Figure 12).

In conclusion, although the intensity of the response of each gene to adverse stress conditions and hormone treatment varied, most *SsNAC* genes responded to drought, low-temperature, salt, and pathogenic fungal stresses and could be induced or repressed by ABA and MeJA.

## Discussion

*NAC* genes play important roles in plant life activities and responses by plants to biotic and abiotic stresses.^38^ Whole-genome analysis of the *NAC* gene family has been conducted in species that have been sequenced, including *O. sativa*,^22^ *Z. mays*,^23,24^ *S. bicolor*,^25^ *Triticum aestivum* L.,^39^ *Musa acuminata*,^40^ *Vaccinium corymbosum* L.,^41^ *Juglans mandshurica*,^42^ *Cleistogenes songorica*, and *Coffea canephora*.^43,44^ The identification and analysis of *S. spontaneum NAC* gene families at the whole-genome level have not been reported. Therefore, the purpose of this study was to study the characteristics of *SsNAC* genes at the genomic level and to provide a reference for further analysis of the potential function of *NAC* genes.

In this study, 115 members were identified by searching for *NAC* genes in the *S. spontaneum* genome. Similar to other Gramineae crops, numerous *NAC* family members were evident, but fewer *NAC* genes than in *S. bicolor* (188) and *Z. mays* (189) were observed. Notably, the *S. spontaneum* genome used in this study was derived from the haploid *AP85–441* (1 n = 4x = 32) produced by the cultivation of octoploid SES208. Therefore, octoploid *S. spontaneum* may contain more than 115 *SsNAC* genes. In genetic evolution and phenotypic evolution, gene duplication plays a crucial role in gene expansion and functional diversification. Homologous genes are generated through tandem duplication and segmental duplication, increasing the total number of genes.^45^ The collinearity analysis of the *SsNAC* genes in this study revealed nine pairs of segmental-duplicated genes and four groups of tandem-duplicated genes, indicating that the expansion of the *SsNAC* gene family may be related to these duplication events. *S. spontaneum* has been reported to have undergone two whole-genome duplication (WGD) events, and its homologous chromosomes were duplicated from one to two and then to four.^30^ *SsNAC* gene duplication is speculated to have occurred during these two WGD events.

*SsNAC* family proteins exhibit significant differences in their characteristics. Similarly, the *SsNAC* family has a very diversified gene structure, but *SsNAC* genes in the same subfamily have relatively conserved gene structures and protein motifs, which lays the foundation for further analyses of their biological functions. Phylogenetic analysis divided *SsNAC* family members into 18 subgroups. The distribution of *SsNAC* family members among the subfamilies was uneven, and some subfamilies did not have a wide distribution of *SsNAC* family members, indicating that the *NAC* gene family diverged after the differentiation of *S. spontaneum* and *A. thaliana* during evolutionary processes. The analysis of the conserved motifs of *NAC* proteins further confirmed the classification of *SsNAC* family members. The motifs at the N-terminus of *NAC* genes are highly conserved and are usually associated with protein interactions, transcriptional activity, and DNA binding ability,^46^ indicating that these conserved motifs are very important for the function of *NAC* genes.

NAC transcription factors play a key role in the regulation of plant growth and development. For example, during cell division in *A. thaliana*, a membrane-bound NAC transcription factor, *NTM1*, is activated by proteolytic cleavage and mediates cytokinin signal transduction;^47^ additionally, *AtNAC1* and *AtNAC2* participate in lateral root development by downregulating auxin signaling.^48^ In addition, *NST1* and *NST3* are involved in the biosynthesis of the secondary wall in *A. thaliana*, including the production of lignin and interfascicular fibers and pod shattering.^49–51^ Similarly, Gossypium spp *GhFSN1* participates in fiber development by activating downstream secondary cell wall-related genes.^52^ The *SsNAC094* gene, which was highly expressed at different stages of the growth and development of the stems and leaves of *S. spontaneum*, is a member of the *Ss_NAC* subgroup, and this gene should be investigated as a key regulator of the growth and development of *S. spontaneum*.

Due to its remarkably high resistance and stress tolerance, *S. spontaneum* is recognized as a key source of stress resistance genes, and modern sugarcane cultivars all contain *S. spontaneum*.^53^ In plants, studies on *NAC* gene regulation in response to drought, salinity, low-temperature, high-temperature, heavy metals, disease, and other stresses have been published.^54–59^ However, few reports about *SsNAC* genes involved in responses to biotic and abiotic stresses are available. Therefore, this study further analyzed the expression patterns and potential functions of 12 *SsNAC* genes in response to various biotic and abiotic stresses. As determined by qRT–PCR, the 12 *SsNAC* genes showed varying degrees of response to drought, salinity, low-temperature, and pathogenic stresses. In particular, the same treatment simultaneously induced the expression of multiple *SsNAC* genes. For example, drought stress induced the simultaneous significant upregulation of the expression of the 12 genes studied, a finding that was also reflected in the RNA-seq data, indicating the reliability of the RNA-seq data. The study further showed that the expression of seven genes, *SsNAC016, SsNAC020, SsNAC028, SsNAC030, SsNAC037, SsNAC077*, and *SsNAC110*, changed significantly in response to salt and low-temperature stresses, indicating that these *SsNAC* transcription factors had a single-cause pleiotropic effect, a finding that has been confirmed in *O. sativa*,^21^ *Z. mays*,^60^ *A. hypogaea*,^61^ and *C. annuum*.^28^ The qRT–PCR results showed that the expression of *SsNAC005* was highly induced by at least one stress factor, therefore, *SsNAC005* can be used as a candidate gene for further studies on sugarcane stress. The expression levels of the 12 *SsNAC* genes after ABA and MeJA treatment varied. Therefore, *NAC* genes may play important roles in the adaptation and resistance of *S. spontaneum* to various environmental stresses.

## Conclusions

In this study, whole-genome analysis was performed on the *SsNAC* gene family, and 115 *SsNAC* genes were identified. A phylogenetic tree analysis divided the *SsNAC* gene family into 18 groups, and the gene structures and protein motifs within the same group were similar. Evolutionary analysis indicated that segmental duplication and tandem duplication were the main evolutionary mechanisms contributing to the expansion of the *NAC* gene family. In addition, *SsNAC* genes had different expression patterns during different developmental stages of *S. spontaneum*. qRT–PCR analysis revealed that the expression patterns of *SsNAC* genes could be induced or repressed by ABA and MeJA, but that the expression patterns under biotic and abiotic stresses were different. In summary, these results provide a basis for further studies on the function of *SsNAC* genes and provide a theoretical basis for genetic improvements in sugarcane resistance to stresses.

## Supplementary Material

Supplemental Material
