# Supplementary material for: Genome-wide identification and expression analysis of the NAC transcription factor family in Saccharum spontaneum under different stresses
Source: Plant Signal Behav. 2022 Jun 22;17(1):2088665. doi: 10.1080/15592324.2022.2088665 (PMC9225438; doi:10.1080/15592324.2022.2088665)
Supplement: Supplemental Material [file KPSB_A_2088665_SM8717.zip › Supplementary Materials/Table S1.pdf]

Table S1. Sequences of primers used in qRT-PCR.

Gene Name	Forward primer (5′ → 3′)	Reverse primer (5′ → 3′)
SsNAC005	GACATCTACAAGTCCGAT	GCTGAAGAAGTACCACTC
SsNAC016	CAAGAAGGGATCGCTCAG	ATCTTCTCCCACTCGTTCT
SsNAC020	TCCAAGGATAGGAAGTAT	TGTTCTGGTAGTTGATTC
SsNAC028	AGGTCGATCTTTACAAGT	GAAGAAGTACCACTCCTT
SsNAC030	CTCCAACTTCTCCTCCAA	CTCCTGCTAGTTTCTTCTTG
SsNAC037	ATCGACCTCTACAAGTTC	GAAGAAGTACCACTCCTT
SsNAC052	AAGATAATGGCTATGTAGG	TCTGATTACTATATGCTCTC
SsNAC077	ACAACAACAACAACAACTT	TTATTAGCAACGATAGAATCAG
SsNAC095	CGTGGATAACAACTTCAA	TAATCATCAGCAGACAGA
SsNAC0107	ACCCAAGTAAAGAGTGCCCAAAGTG	TGCCCGTAAGTGCCAGATTAATTCC
SsNAC0110	CAATCCAAGTTGTCCATA	TTATCTCCATCCTCTGTT
SsNAC0111	TGACCTACTACCTGAAGA	GCACTTCTCTTGGATGTC
GAPDH	CACGGCCACTGGAAGCA	TCCTCAGGGTTCCTGATGCC
