# Supplementary material for: Genome-wide identification and expression analysis of the NAC transcription factor family in Saccharum spontaneum under different stresses
Source: Plant Signal Behav. 2022 Jun 22;17(1):2088665. doi: 10.1080/15592324.2022.2088665 (PMC9225438; doi:10.1080/15592324.2022.2088665)
Supplement: Supplemental Material [file KPSB_A_2088665_SM8717.zip › Supplementary Materials/Table S2.pdf]

Table S2.The nucleotide and protein sequences of *SsNAC* gene family

Gene ID	Gene name	gene sequence	protein sequence
Sspon.01G0001190-1A	*SsNAC001*	ATGGCGCCAGTCAGTTTGCCTCCT<br>GGTTTCAGGTTCCACCCCACTGAC<br>GAGGAACTCATC<br>ATCTACTACCTTAAGAGGAAGAT<br>CAACGGGAGACAGATAGAGCTCG<br>AAATCATTCCAGAG<br>GTTGATCTTTACAAGTGTGAGCCC<br>TGGGATTTGCCAGAAAAATCCTTT<br>CTTCCAAGCAAA<br>GACCTTGAATGGTATTTCTTCAGC<br>CCTCGAGACCGCAAGTACCCAAA<br>TGGATCAAGGACA<br>AACCGTGCAACAAAATCTGGGTA<br>CTGGAAGGCAACTGGGAAGGACA<br>GAAAGGTGAACTCG<br>CACAGGCGTGCAGTTGGTATGAA<br>GAAGACCTTGGTGTACTATCGGG<br>GCCGAGCTCCACAT<br>GGTTCTCGCACTGATTGGGTCATG<br>CACGAGTACCGCCTCGACGAGAG<br>GGAATGCGAGACT<br>GACACTGGCTTACAGGATGCGTA<br>TGCATTATGCCGAGTGTTTAAGA<br>AGACAGCGCCTGGG<br>CCAAAAATCATAGAGCATTATGG<br>CGCAGTGCACCACCCCATCGAGC<br>AACCTCAGTGGATG	MAPVSLPPGFRFHPTDEELII<br>YYLKRKINGRQIELEIIPEVD<br>LYKCEPWDLPEKSFLPSK<br>DLEWYFFSPRDRKYPNGSR<br>TNRATKSGYWKATGKDRK<br>VNSHRRAVGMKKTLVYYR<br>GRAPH<br>GSRTDWVMHEYRLDEREC<br>ETDTGLQDAYALCRVFKKT<br>APGPKIIEHYGAVHHPIEQP<br>QWM<br>ASSVDRSPTLDLSSDVRGD<br>DFESSSFSFPTEAPMDSMHG<br>GFGMQMSAPHEDGKWMQF<br>LSE<br>DAFNATNPFFMNPASSSFSC<br>LPSKVDVALECARLQHRLS<br>LPPLEVEDFPQDVSLDTKTS<br>V<br>LRSNPNEVDILQEFLSVASA<br>SQELINGTSSSYAAEMWPG<br>AGTSSTSTHYINELSSLVEL<br>G<br>VKAKEEADNFYHMDCIGTS<br>AGFASKQAHVDEPVRLVEI<br>ADMEEFKQEEKRQVENLRG<br>VRL

Sspon.01G0001940-1A	*SsNAC002*	ATGGCAATCATGGCCGGGGCGCC<br>GTCCATGGAGGTGGAGCAGGACC<br>TCCCGGGCTTCCGG<br>TTCCACCCCACCGAGGAGGAGCT<br>CCTCGGCTTCTACCTCTCCCGCGT<br>CGTCCACGGCAAG<br>CAGCTCCACTTCGACATCATCGGC<br>ACCCTCAACATCTACCGCCACGA<br>CCCCTGGGACCTT<br>CCTGCGATGGCGAAGATCGGGGA<br>GCGGGAGTGGTACTTCTTCGTGCC<br>GCGCGACCGCAAG<br>GCCGGCAGCGGCGGGCGGCCGAA<br>CCGGACGACGGAGCGTGGGTTCT<br>GGAAGGCCACGGGG<br>TCGGACAGGGCCGTCCGGAGCTC<br>CGCCGACGCCAAGCGGGTGATCG<br>GGCTCAAGAAGACG<br>CTCGTCTTCTACCAGGGGCGCGCC<br>CCGCGCGGCACCAAGACGGACTG<br>GGTCATGAACGAG<br>TACCGCCTCCCCGACCGCGCCGCC<br>GCCGGCCGCGCCGCGCCGCCACC<br>TCCCAAGGAGGAC<br>ATGGTGCTCTGCAAGATCTACAG<br>GAAGGCCACTCCACTGAAGGAGC<br>TGGAACAGAGGGCC	MAIMAGAPSMEVEQDLPGF<br>RFHPTEEELLGFYLSRVVHG<br>KQLHFDIIGTLNIYRHDPWD<br>L<br>PAMAKIGEREWYFFVPRDR<br>KAGSGGRPNRTTERGFWKA<br>TGSDRAVRSSADAKRVIGL<br>KKT<br>LVFYQGRAPRGTKTDWVM<br>NEYRLPDRAAAGRAAPPPP<br>KEDMVLCKIYRKATPLKEL<br>EQRA<br>SAMEEMQRRSSAARAASLV<br>QAAGSAADDYLSVSSDDAH<br>DSSFLQLPSSSSSAPSGDSYG<br>A<br>PAPREAKTEAADAMAITVA<br>STSSMAAVQPPAVRHADLP<br>TLQVPTNNLGVADWTQMQ<br>DPFQ<br>LRSPWQDQLFFSPLAHLLY

Sspon.01G0007700-1A	*SsNAC003*	ATGAAGCTTTGGAGGACGGAGCC<br>CCGCGGCAAGGCCGCGGCCCCGG<br>GGCGTGACGATCCC<br>GTCCGCGTCCTGGTGACGATGAC<br>CATCCTTGGGAGCGTCGGCCCGC<br>AGCGCTTCATCGTG<br>CTTGAGGGAGACATGGTCGCCGC<br>GCTCCGCCGCTACGCCCGAGAGG<br>GCTGCATGCCGCTG<br>CTCGGCACCGACCCCGCCGGCTTC<br>GTCCTCTCCACCGCCAACGGCGG<br>ATCCGGCGGTACC<br>GCTCAAAGAAGACGAGAAGATCA<br>CCTTCAACGGGTGCCAGAGCTTCC<br>TGCTCTGGCAGAA<br>GGATGCGGTCCAGGGTGCCACCA<br>ACCGCGCCGGGCCCGATGCTTTG<br>ACAGCAACCCGAGC<br>CCTAGCAGGAAGGGCAGCGGTGG<br>CTTCAAGGCTACTCCAAGCCCTGG<br>CAGGAAAGGCAGT<br>TGTGGCCGGAAGGCTGGCCTAAA<br>CAAGTTCCTCCTCAGCTTCAGCAT<br>CAAGTTAGATCGT<br>GGAGCAATTGCAACACGTATATA<br>TTTGGACACTTCAGACAATACATC<br>CCGGGCTGGACGA	MKLWRTEPRGKAAAPGRD<br>DPVRVLVTMTILGSVGPQR<br>FIVLEGDMVAALRRYAREG<br>CMPL<br>LGTDPAGFVLSTANGGSGG<br>TAQRRREDHLQRVPELPAL<br>AEGCGPGCHQPRRARCFDS<br>NPS<br>PSRKGSGGFKATPSPGRKGS<br>CGRKAGLNKFLLSFSIKLDR<br>GAIATRIYLDTSDNTSRAGR<br>RHFDSHHRVAPQSWPGLPR<br>GVEFNPSDSDILWHLAAEV<br>GNGLIERHPFIHEFIKFVDGD<br>G<br>GFYCRHPQDIPGVSQDGRA<br>SYFFHRSFEPYNNENDANN<br>CWKKIGSPRSIILDGTLQGC<br>KE<br>VFALYADMPSDKRSQETN<br>WRLHRYHLQNTVKAESEIV<br>VSKIFLASRSNLCELAEEARI<br>ES<br>ERYVSTHKDSSVECAKGFN<br>PEICTETDELDHIPLKERYRI<br>LLADKSSVLATVSSGKSIIY<br>STEKMLEEDAYTVKEGVAY

Sspon.01G0008840-1A	*SsNAC004*	ATGTCTGAGGTGTCGGTGATAAA<br>CCAGGCGGAGGTGGAGGATGCGG<br>GTGCCGGGCAGCTG<br>GACCTGCCGCCGGGCTTCCGCTTC<br>CACCCCACCGACGAGGAGATCAT<br>CTCGCACTACCTC<br>ACCCACAAGGCCCTCAACCACCG<br>CTTCGTCTCCGGCGTCATCGGCGA<br>GGTCGACCTCAAC<br>AAGTGCGAGCCATGGGACCTCCC<br>AGGCAGGGCCAAGATGGGGGAAA<br>AGGAGTGGTACTTC<br>TTCTGCCACAAGGATCGCAAGTA<br>CCCGACGGGCACACGGACCAATC<br>GCGCCACGGAGACC<br>GGCTACTGGAAGGCCACCGGCAA<br>GGACAAGGAGATCTTCAGGGGCC<br>GCGGCATCCTCGTG<br>GGCATGAAGAAGACGCTCGTCTT<br>CTACCGCGGCCGCGCCCCGCGCG<br>GGGAGAAGACCGGC<br>TGGGTCATGCACGAGTTCCGCCTC<br>GAGGGCAAGCTTCCCCACCCGCT<br>CCCGCGCTCCGCC<br>AAGGACGAGTGGGCCGTGTGCAA<br>GGTGTTCAACAAGGAGCTGGCGG<br>CAAGGACCGAGCCA	MSEVSVINQAEVEDAGAGQ<br>LDLPPGFRFHPTDEEIISHYL<br>THKALNHRFVSGVIGEVDL<br>N<br>KCEPWDLPGRAKMGEKEW<br>YFFCHKDRKYPTGTRTNRA<br>TETGYWKATGKDKEIFRGR<br>GILV<br>GMKKTLVFYRGRAPRGEKT<br>GWVMHEFRLEGKLPHPLPR<br>SAKDEWAVCKVFNKELAA<br>RTEP<br>MAAAAGAELERVGSLGFLN<br>ELLDSAELPALIGADVDEVI<br>DFKGPASTSGHAGAPGTSY<br>LP<br>VKMEEYALLQMQYQQQQP<br>PPMFYSSQYFSLPAMNSGD<br>LPPAIRRYCKAEQQVVSSGQ<br>TAS<br>VVSPSRETGLSTDPNAAGG<br>YAEISSAVTPSSSSHQFLPEL<br>DDSVLNLADLW

Sspon.01G0008870-1A	*SsNAC005*	ATGATCATGGCGAACCCGGACAT<br>GCTGCCGCCGGGGTTCCGGTTCCA<br>CCCGACGGACGAG<br>GAGCTGATCCTCCACTACCTCCGC<br>AACCGCGCCGCCAACGCGCCGTG<br>CCCGGTCGCCATC<br>ATCGCCGACGTCGACATCTACAA<br>GTCCGATCCATGGGACCTGCCATC<br>CAGGGCTGCGTAC<br>GGGGACAAGGAGTGGTACTTCTT<br>CAGCCCGCGCGACCGCAAGTACC<br>CGAACGGGACCCGG<br>CCGAACCGCGCGGCAGTGTCCGG<br>CTACTGGAAGGCCACCGGCACCG<br>ACAAGCCCATCCAG<br>AGCAGCGCCACCGGCGAGAGCGT<br>CGGCGTCAAGAAGGCGCTCGTCT<br>TCTACAAGGGCCGC<br>CCGCCCAAGGGCACCAAGACCAA<br>CTGGATCATGCACGAGTACCGCC<br>TCGCCGCCGACGCG<br>CACGCCGCACACACCTACCGCCC<br>CATGAAGTTCCGCAACGCCTCCAT<br>GAGGCTGGATGAC<br>TGGGTGCTGTGCCGGATCTACAA<br>GAAGGCCAGCCACGCGCCGCCGA<br>TGTCGGTGCCGCCG	MIMANPDMLPPGFRFHPTD<br>EELILHYLRNRAANAPCPVA<br>IIADVDIYKSDPWDLPSRAA<br>Y<br>GDKEWYFFSPRDRKYPNGT<br>RPNRAAVSGYWKATGTDK<br>PIQSSATGESVGVKKALVFY<br>KGR<br>PPKGTKTNWIMHEYRLAAD<br>AHAAHTYRPMKFRNASMR<br>LDDWVLCRIYKKASHAPPM<br>SVPP<br>LSDHELQDEPCGGFDDNPY<br>AATSAAMLLQGASFPVLHA<br>ASAGAQRMPRIPSFSELFND<br>PS<br>LLAHFFEEGGMQQDMPRLG<br>NQQQHAPLLGRPVTSQLLV<br>NSGSSMSPGQIQQMDPPAST<br>SA<br>AGDGAAGKRKRSSEAITAS<br>ASALSSQQQASAAKKPNGY<br>CFGATTTFQIGNGLQGSSLG<br>HQ<br>MQLYSSNMGMN

Sspon.01G0013390-1A	*SsNAC006*	ATGGAGTCATGCGTCCCTCCTGGG<br>TTTAGGTTTCACCCCACGGACGAG<br>GAGCTCGTCGGC<br>TACTACCTCCGGAAGAAGGTGGC<br>CTCGCAGAAGATCGACCTCGACG<br>TCATCCGCGACGTT<br>GACCTGTACCGCATTGAGCCATG<br>GGATCTCCAAGAACACTGCAAGA<br>TTGGTTACGAGGAG<br>CAGAGTGACTGGTACTTCTTCAGC<br>TACAAGGACCGCAAGTACCCGAC<br>GGGGACGCGCACG<br>AACCGGGCCACGCTGACGGGGTT<br>CTGGAAGGCGACCGGGCGGGACA<br>AGGCGGTGCGCGGC<br>AGCAAGCAGGGCGCCGGGCTCAT<br>CGGCATGCGGAAGACGCTCGTCT<br>TCTACACGGGGAGG<br>GCGCCCAACGGCCGCAAGACCGA<br>CTGGATCATGCACGAGTACAGGC<br>TGGAGACCGACGAG<br>AACGCCGCACCTCAGGCAAGACC<br>CCTAGTCATCTGCGTGCTGCTTCT<br>GTCGCGCTTACCT<br>AGCCTCGGCCGGCCGCACCTACA<br>CTGTCCGCCGGAACCAACGGGGT<br>ACCAGGATGGGGCC	MESCVPPGFRFHPTDEELVG<br>YYLRKKVASQKIDLDVIRD<br>VDLYRIEPWDLQEHCKIGY<br>EE<br>QSDWYFFSYKDRKYPTGTR<br>TNRATLTGFWKATGRDKA<br>VRGSKQGAGLIGMRKTLVF<br>YTGR<br>APNGRKTDWIMHEYRLETD<br>ENAAPQARPLVICVLLLSRL<br>PSLGRPHLHCPPEPTGYQDG<br>A<br>TGSAGKFQCLFGMALISKID<br>RISNRTIGPENEEGWVVCRA<br>FKKRTMHPPRSVAGAGSW<br>DP<br>SYSYYHDPIVVASAARFKQ<br>ESPEDGAAAAASAALLQYS<br>SRLAELPQLESPPLMPHQGS<br>HR<br>AVADGEGDSAATTDWRAL<br>DRFVASQLSPDEERSGQGL<br>HPEYCGGKQPLGTHAGDNE<br>DATD<br>MAALLLLDGVWHGEAGLL<br>GS

Sspon.01G0015060-1A	*SsNAC007*	ATGGGAACCATGACTCTGCCGCC<br>CGGGTTCCGGTTCCACCCGACGG<br>ACGACGAGCTGGTG<br>GGCTACTACCTCAAGAGGAAGGT<br>GGACAACCTCAAGATCGAGCTCG<br>AGGTCATCCCCGTC<br>ATTGATCTCTACAAATCCGAGCCA<br>TGGGAACTACCAGGTTACCCCAA<br>CGGTTCCCGCACC<br>AACCGCGCCACGACCACGGGGTA<br>CTGGAAGGCCACGGGCAAGGACC<br>GCCGCATTGCCTGC<br>GACGGCGGCGTCTACGGCCTCCG<br>CAAGACGCTCCGCACCGACTGGG<br>TCATGCACGAGTAC<br>CGCCTCTGCCAGGACATCGCCCA<br>CGGCGCATGCAACTTCATCCGGC<br>ACGAGGCCGGGCTG<br>CTGCAGGGCGAGCCGGCGGCGGG<br>CAGGGCGAAAGCAGCCGCCGGCA<br>GCAGCGCGAGAGGG<br>CAGATGAGCAAGGTCTCCAGCAG<br>CTCGTCCCTCGTCAGCAGCGAGC<br>AGCTCAGCGCGTCG<br>TTCACGACCACCAACTCCACCAGT<br>CCACCTCCTACTCTGGACGTGGGC<br>AGCAGGGGAATA	MGTMTLPPGFRFHPTDDEL<br>VGYYLKRKVDNLKIELEVIP<br>VIDLYKSEPWELPGYPNGSR<br>T<br>NRATTTGYWKATGKDRRIA<br>CDGGVYGLRKTLRTDWVM<br>HEYRLCQDIAHGACNFIRHE<br>AGL<br>LQGEPAAGRAKAAAGSSAR<br>GQMSKVSSSSSLVSSEQLSA<br>SFTTTNSTSPPPTLDVGSRGI<br>MCTVSESGNPFQSPVGYGV<br>TATATTGTGLPSSPLPPPLLL<br>SSPHDTFFIGDDFPAAAAEE<br>SRSHAHLFGDFMGMGMEM<br>GGVSDQELKWDSFACPTTF<br>SSGRCILPRFLLNAALRAPD<br>RAD<br>TWNAAAAVASATMLCRQA<br>SDGIEDLAAIFFSDDNRIVF

Sspon.01G0023330-1A	*SsNAC008*	ATGGCCATGGCCATGGCCGATGG<br>CCCTCGAGGGGGCTTTGCTCTCCG<br>CCCCAGCGATCAC<br>GACCTGATCACGTTCGTCCTCCAC<br>CCCATGTTCGCCCGGCAGCAGCG<br>CTTCGACGGTGCT<br>GGCTTCGTCGTCCACCTGGCCGAC<br>GTCTACTCCGTCGCCCCCGAGAG<br>GCTGACCAAGCGG<br>TACGCGCCGGCGCCGGGCAGGAC<br>CTGCTGGTACTTCATCTGCCCCGC<br>ACGCTGCCGCCAC<br>ATGGCAGCGGCAGGCGCCCTGGG<br>CGAGGGGCGATGGAACTCGGAGA<br>CCGGCGAGGTCAGC<br>CCCGTCAGGGGCGCGGACGGCCG<br>CTGCGTCGGGTACTCGCGCACGTT<br>GTCCTACTCCTAC<br>GGCGGCGCCAGGATGACGACGAC<br>GCCGTGGACGACGGTCACCAGGC<br>ACGGCTGGTGCATG<br>GTGGAACTCGCGCTCGACGACGA<br>GCACGACGGTGGCGGTGTGGGAG<br>GAGGAGATTTCGTC<br>CTGTGCAATTTGTTCAGGTCGTCG<br>CCTCACAATGAGGTAGCCGTGCC<br>TGCGCTCCTCTTC	MAMAMADGPRGGFALRPS<br>DHDLITFVLHPMFARQQRF<br>DGAGFVVHLADVYSVAPER<br>LTKR<br>YAPAPGRTCWYFICPARCR<br>HMAAAGALGEGRWNSETG<br>EVSPVRGADGRCVGYSRTL<br>SYSY<br>GGARMTTTPWTTVTRHGW<br>CMVELALDDEHDGGGVGG<br>GDFVLCNLFRSSPHNEVAV<br>PALLF<br>NCKRKAVGDHTEAPPSVRQ<br>QLLHHLDPEAVFHHLHNRG<br>MPNTVSST

Sspon.01G0023640-1A	*SsNAC009*	ATGGAGGAAGGGCTCCCGCCGGG GTTCCGGTTCCACCCGACGGACG AGGAGCTGGTCACC TACTACCTCACCCGCAAGGTCTCC GACTTCGCCTTCGCCACCCGCGCC ATCGCCGACGTC GACCTCAACAAGTGCGAGCCATG GGACCTCCCAAGCAAAGCTAGCA TGGGTGAGAAGGAA TGGTACTTCTTCAGCATGCGTGAC AGGAAATACCCGACGGGAATCCG GACGAACCGCGCC ACCGACTCAGGCTACTGGAAGAC GACGGGCAAGGACAAGGAGATCT TTCACTGCGGCATG CTTGTGGGGATGAAGAAAACCCT AGTCTTCTACAGGGGCAGGGCTC CCAAGGGCCAGAAG ACCAGCTGGGTCATGCACGAGTA CAGGCTCCAGAACAAGTTCCCCT ACAAACCAAACAAG GAGGAATGGGTGGTGTGCAGGGT GTTCAAGAAGTGCCAGGTCATCA AGATGAGACCGCCG CAGGACAGCCCCACGATGGGCTC CCCATGCCACGACGCCGCCAACG CCTCCCTGGGCGAG	MEEGLPPGFRFHPTDEELVT YYLTRKVSDFAFATRAIAD VDLNKCEPWDLPSKASMGE KE WYFFSMRDRKYPTGIRTNR ATDSGYWKTTGKDKEIFHC GMLVGMKKTLVFYRGRAP KGQK TSWVMHEYRLQNKFPYKP NKEEWVVCRVFKKCQVIK MRPPQDSPTMGSPCHDAAN ASLGE LGELDVSSILGGLASSGSPP GALHHQGSAGAESFGAHRP VDMSAYMSWMAAAANQG AAAA AAMLPWATTPPPGLFGNVF APNNHQLLQKPLPFAGCSQ PRELGGVVANVIGSGEHTM FGS SVVK

Sspon.01G0029280-1A	*SsNAC010*	ATGGTGGCCAAGGAGTTTGGAAG<br>AGAAGTAATAAGCATGGACAAGG<br>TGAAGAGAGACGGA<br>GAGGCGCTCATCACCACCGGAGC<br>CGGAGACGAAGAGGAAGATGACG<br>TGGTGCTCCCCGGG<br>TTCCGGTTCCACCCGACTGACGAG<br>GAGCTCGTCACGTTCTACCTCCGC<br>CGGAAGGTGGCG<br>AGGAAGCCGCTCAGCATGGAGAT<br>CATCAAGGAGATGGACATCTACA<br>AGCACGATCCATGG<br>GACCTCCCTAAGGCGAGCACGGT<br>TGGTGGAGAGAAGGAATGGTACT<br>TCTTCTGCCTGAGA<br>GGAAGGAAGTACCGGAACAGCAT<br>CCGGCCCAACAGGGTCACCGGCT<br>CCGGCTTCTGGAAG<br>GCCACCGGCATCGACCGCCCGAT<br>CTACTCTGTCGCTGCTGGTGCCGC<br>CGCTGCAAACTCC<br>GGCGAGTCCATCGGGCTCAAGAA<br>GTCCCTCGTGTACTACCGCGGCAG<br>CGCCGGCAAGGGC<br>ACCAAGACCGACTGGATGATGCA<br>CGAGTTCCGCCTCCCGCCGGCCGC<br>CACCGACGCCTCC	MVAKEFGREVISMDKVKR<br>DGEALITTGAGDEEEDDVV<br>LPGFRFHPTDEELVTFYLRR<br>KVA<br>RKPLSMEIIKEMDIYKHDPW<br>DLPKASTVGGEKEWYFFCL<br>RGRKYRNSIRPNRVTGSGF<br>WK<br>ATGIDRPIYSVAAGAAAANS<br>GESIGLKKSLVYYRGSAGK<br>GTKTDWMMHEFRLPPAAT<br>DAS<br>RACRKLIFKRNIAFKRQPQQ<br>QPAWRQQVGSNAPPPMLAE<br>SSSNTGGFESDGGGDEYMN<br>CL<br>PVPAVALGMPRLHRIGSML<br>NGGGVSVTGSSFFREGVHS<br>QQFQGQWLNRFPAPAIEQK<br>PQL<br>LDSSAMTIAFHQNDQSVAA<br>AAMTNDQCYKDGYWDEIA<br>RFMEVNDPTVLYDRRYA

Sspon.01G0030860-3D	*SsNAC011*	ATGGAGGGCCCCGAGAAGTACTA<br>CGAGTTCAGCCCAAGCGATCAGA<br>GGCTGATCGACGTC<br>TACCTCCGCTCCAAGATCGCCGGC<br>AAGGATGTGGGCGGCGGCTCCAT<br>CCACGACGTCGAC<br>GTGACCTCCGATCACCCCTACAAT<br>CTCGTGCGCAAGCACGCGCCCGC<br>CCCTGGGAGTCTC<br>ACCGGCGGTGGAGACAAGGGAGT<br>CTGGTTCTTCTTCAGCCCGAAGCG<br>CTACATCGGGAAT<br>TCGAAGGCGAGCGCGCGCAGCGG<br>ATCTCGGCTCACGAACCGGGCGC<br>GAACCGTGCTCGGC<br>GTCGGCGGCAGGAAGAAGGGGGC<br>GTGGCACACGGAGGGTAGGAAGA<br>AGACGGTGCCTGGA<br>AGCGCTGGCGGGTACTTTCAGAA<br>GCTGTCGTACGAGGAGGTGACGC<br>CGTCGGGGTCGGTC<br>GTCAAGCCGGGATGGCTGATGAT<br>CGAGTACGCCATCGAGGAAGAGC<br>ACGGCGGCTGTGGC<br>GCCATGGTCCTGTGCAAGGTGTA<br>CAAGTCACCACGAGGCCCTGGAT<br>CGGACGTGCCGTCG	MEGPEKYYEFSPSDQRLIDV<br>YLRSKIAGKDVGGGSIHDV<br>DVTSDHPYNLVRKHAPAPG<br>SL<br>TGGGDKGVWFFFSPKRYIG<br>NSKASARSGSRLTNRARTV<br>LGVGGRKKGAWHTEGRKK<br>TVPG<br>SAGGYFQKLSYEEVTPSGS<br>VVKPGWLMIEYAIEEEHGG<br>CGAMVLCKVYKSPRGPGSD<br>VPS<br>LSRKRKADVVEQPVVEQLQ<br>RPCKRTHREVMFLAGTLLC<br>EDLTETDYPAAVPVAYQET<br>ERT<br>LGRSGESATPADQCIGKTDQ<br>VPRRIQEDARDELTRFWESL<br>ATGHDEMALDYLLQEDQP<br>VP<br>PCSSRTEDEDDVMEISFEEF<br>RGSSSLAGAVPPPCAAQPED<br>AAISCVQMSEQKVVPIDDDS<br>EIEFTFEELMMGSSTPGGYS<br>SMPSPRTLTDDDEDDDDDM<br>RGLACPTMDYAILEALVEL<br>VA

Sspon.01G0031460-1A	*SsNAC012*	ATGCGCACTGCGCCAGAGGCAAG<br>GCAATATAAGGAGACCTGCATCT<br>GCATGGGCTGCGCT<br>GCCACGCACACCTTCACGTCGATC<br>GTCGTAGATTCCACTGCTCCCATC<br>GATCGTTCCACG<br>CCCACGCCCAGATCCACCGCCAC<br>CACCTCGAGACCGGCGGCATGGC<br>CACCAACGAGGCCC<br>ACCGACGAGGCATCGGTCGCCCT<br>CCTGCGCGGCCTCCGTGCCAAAA<br>CCATCAACCGCAGC<br>TTCATCCACTGCGCCGACGTGTGC<br>TCCGCCGCCCCCGAGGAGCTGGT<br>CGCCGATCTACAG<br>CCGGTGCCGGGCACCGACGTGAC<br>GGAGGACGGGTACAACAGCGTTT<br>GGTACATCTACTGC<br>CCGAAGCGATACAAGAATACCCG<br>GGGCAAGGCCAGCGGGCACAGGC<br>AGCGCGCGGTGGAG<br>CCCGACGGCAGCAAGATGTGCTG<br>GCACTCGGAGGCTGGCCCGAAGC<br>CCGTGCAAGGCGTG<br>CCCGGCGCCACGTTCTGCAACTTC<br>TCCTACGGCCGCAAGGAGAAGGA<br>GGGGGCGTCGGGT	MRTAPEARQYKETCICMGC<br>AATHTFTSIVVDSTAPIDRST<br>PTPRSTATTSRPAAWPPTRP<br>TDEASVALLRGLRAKTINRS<br>FIHCADVCSAAPEELVADLQ<br>PVPGTDVTEDGYNSVWYIY<br>C<br>PKRYKNTRGKASGHRQRA<br>VEPDGSKMCWHSEAGPKP<br>VQGVPGATFCNFSYGRKEK<br>EGASG<br>RQRLDRMGWCMTEFDDTQ<br>GGGGDHVLCRVHRSSSSLA<br>KRKFKSSSSSSKRKAAGDH<br>PGAP<br>PTKLIHDKQVQQNPAVFFT<br>NAYPTMPCYGYGPPQGVDP<br>VTMVGGEEDHGVQQPRVE<br>PEYG<br>GEFNFDELLNIDEVVQMQD<br>PAAFFTNAYPTMPCYGYGP<br>PEDVDPVTLVGGEEEHGVQ<br>QPR<br>VELEAEHVDSGEFNVEELL<br>NMEEFTNAYPTMTMPCYV<br>HGGPQGVDDPAMFGGEEE<br>HGVQQ

Sspon.01G0031480-3D	*SsNAC013*	ATGGCCACCAACGACGAGTTCCG<br>GCCCAACGACGAGGCATCGGTCG<br>CCCTCCTGCGCGGC<br>CTCCGTGCCAAAACCATCAACCG<br>CAGCTTCATCCACTGCGCCGACGT<br>GTGCTCCGCCGCC<br>CCCGAGGAGCTGGTCGCCGATCT<br>ACAGCCGGTGCCGGGCACCGACG<br>TGACGGAGGACGGG<br>TACAACAGCGTTTGGTACATCTAC<br>TGCCCGAAGCGATACAAGAATAC<br>CCGGGGCAAGGCC<br>AGCGGGCACAGGCAGCGCGCGGT<br>GGAGCCCGACGGCAGCAAGATGT<br>GCTGGCACTCGGAG<br>GCTGGCCCGAAGCCCGTGCAAGG<br>CGTGCCCGGCGCCACGTTCTGCA<br>ACTTCTCCTACGGC<br>CGCAAGGAGAAGGAGGGGGCGTC<br>GGGTCGGCAGCGCTTGGATAGGA<br>TGGGGTGGTGCATG<br>ACAGAGTTCGACGACACGCAGGG<br>CGGCGGCGGCGACCATGTGCTGT<br>GCAGGGTGCACCGC<br>TCGTCCTCGTCTCTCGCCAAAAGG<br>AAGTTCAAGTCGTCGTCGTCGTCG<br>TCCAAGAGGAAG	MATNDEFRPNDEASVALLR<br>GLRAKTINRSFIHCADVCSA<br>APEELVADLQPVPGTDVTE<br>DG<br>YNSVWYIYCPKRYKNTRGK<br>ASGHRQRAVEPDGSKMCW<br>HSEAGPKPVQGVPGATFCN<br>FSYG<br>RKEKEGASGRQRLDRMGW<br>CMTEFDDTQGGGGDHVLC<br>RVHRSSSSLAKRKFKSSSSS<br>SKRK<br>AAGDHPGAPPTKLIHDKQV<br>QQNPAVFFTNAYPTMPCYG<br>YGPPQGVDPVTMVGGEED<br>HGVQ<br>QPRVEPEYGGEFNFDELLNI<br>DEVVQMQDPAAFFTNAYPT<br>MPCYGYGPPEDVDPVTLVG<br>GE<br>EEHGVQQPRVELEAEHVDS<br>GEFNVEELLNMEEFTNAYP<br>TTTMPCYVHGGPQGVDDP<br>AMFG<br>GEEEHGVQQPLVESEAEHG<br>SDLGGFNLEELLNTDDWKG<br>FLQMLQAEVEQGQQQPCAE

Sspon.01G0032330-1A	*SsNAC014*	ATGCGCTTTCGCGCAACATCTCCT<br>TGCTGCCGCTGCTATCCAAGATCA<br>TCCACGAGCAGG<br>CCAACCATCAGCACAGGCGACGC<br>GAAAGACATCTACGTGAGTAGGG<br>AATGGAGGCGCATC<br>AAGGCAGCTAGCACAGCACGACA<br>CCATAACAAAACAGGAGGGCCAG<br>GCGGCTACTTCATC<br>TACTACGCGCCGCCAGCGGAGGG<br>TTGTCCCATGGGAGAGGCAGCCA<br>CATCTGGCACCATA<br>TGGCCAGAGCAAGGCGAGGACGA<br>CGCAAGGGCAGGAGGAAGACGG<br>GCGACCGCGGCGGGC<br>ATGAAGCGTGATGGAGGGGGGGG<br>AGGCGGGCAGCCGTGCTCGGGCG<br>CGGTGGAGATGCCG<br>GCCGTGCTCAAGAGAGATACGGT<br>GGAGGGTGGGGAGCGAGGCCTGG<br>GCGGACTGCAACTC<br>GTGAAGGACTCCACAGTCGGCCA<br>CACCAAGGCAACCATGGCCAGCA<br>CCACCCCACTGCTG<br>CCCTCCCAGTTCCTCAGGGGCGTG<br>CCGCAGTGTCTTCCTACCTCCTGC<br>TCACTGGCAGGA	MRFRATSPCCRCYPRSSTSR<br>PTISTGDAKDIYVSREWRRI<br>KAASTARHHNKTGGPGGYF<br>I<br>YYAPPAEGCPMGEAATSGT<br>IWPEQGEDDARAGGRRATA<br>AGMKRDGGGGGGQPCSGA<br>VEMP<br>AVLKRDTVEGGERGLGGLQ<br>LVKDSTVGHTKATMASTTP<br>LLPSQFLRGVPQCLPTSCSL<br>AG<br>AGAAPLGPRTGCRAWLPPG<br>PRQVLVAWSWATCTCWAC<br>CRTRTYESWHGGAPRDAV<br>ATEHG<br>AAREMLKAHDIDCCSRPAS<br>PGPKRLSYDLKDVAFAPFG<br>EYWREMRSLLVVELLSTRR<br>VDK<br>LVANLTRAARASTPVELKE<br>YIFGLADGIIGAVAFGNIYGT<br>EWFAHRERRFQLVVEEGM<br>DL<br>LATFSAEDFFPNAAGCLLVF<br>RDLDAFYEMVVDQHTDPA<br>RPEPVDALINLCKHRGTFRF

Sspon.01G0034390-1B	*SsNAC015*	ATGGACTCCATGGAATCATGCGT<br>GCCCCCGGGCTTCAGGTTCCACCC<br>CACCGACGAGGAG<br>CTCGTCGGCTACTACCTCAGGAA<br>GAAGGTCGCCTCCCAGAAGATCG<br>ACCTCGACGTCATA<br>CGCGACATCGATCTCTACCGCATC<br>GAGCCATGGGATCTCCAAGAACA<br>TTGCGGGATCGGG<br>TACGAGGAGCAGAACGAGTGGTA<br>CTTCTTCAGCTACAAGGACCGCA<br>AGTACCCGACAGGG<br>ACGCGGACCAACAGGGCCACCAT<br>GGCCGGGTTCTGGAAGGCAACGG<br>GCAGGGACAAGGCG<br>GTGCACGACAAAAGCCGCCTCAT<br>TGGCATGAGGAAGACACTCGTCT<br>TCTACAAGGGAAGG<br>GCACCCAACGGGCAGAAGACCGA<br>CTGGATCATGCATGAGTACCGGC<br>TCGAGACCGACGAG<br>AATGCACCGCCTCAGGCAAGCTC<br>CCTCACCATGATCTTAGAAGAAG<br>GCTGGGTGGTGTGC<br>CGGGCGTTCAAGAAGAGAACAGC<br>GTACCCGGCAAGGAGCATGGCTA<br>TGGCATGGGACCCC	MDSMESCVPPGFRFHPTDE<br>ELVGYYLRKKVASQKIDLD<br>VIRDIDLYRIEPWDLQEHCGI<br>G<br>YEEQNEWYFFSYKDRKYPT<br>GTRTNRATMAGFWKATGR<br>DKAVHDKSRLIGMRKTLVF<br>YKGR<br>APNGQKTDWIMHEYRLETD<br>ENAPPQASSLTMILEEGWV<br>VCRAFKKRTAYPARSMAM<br>AWDP<br>SYAYRDVSAMGAAAAAEV<br>AAFVDPNAAYAQIRRQSNK<br>SARFKQEAELDGAAAALLQ<br>YSSS<br>HLFELPQLESPSAPLAPANQ<br>SQASGADEVVDGTDSGRRP<br>GKKARADKVATDWRALDK<br>FVA<br>SQLSPAAECGGSLEAAAAA<br>ASTVAASNVACSQLDHGED<br>DDMAALLFLNSDGREEAER<br>WTG<br>LLGPAGGDGDFGLCVFEK

Sspon.01G0042440-1B	*SsNAC016*	ATGGGACTGCCGGTGAGGAGGGA<br>GAGGGACGCGGAGGCGGAGCTGA<br>ACCTGCCGCCGGGG<br>TTCCGGTTCCACCCCACCGACGAC<br>GAGCTGGTGGAGCACTACCTGTG<br>CCGGAAGGCGGCG<br>GGGCAGCGCCTCCCGGTCCCCAT<br>CATCGCCGAGGTGGACCTCTACA<br>AGTTCGACCCCTGG<br>GACCTGCCGGAGCGCGCTCTT<br>CGGGGTCAGGGAGTGGTACTTCT<br>TCACGCCCAGGGAC<br>CGCAAGTACCCCAACGGGTCCCG<br>CCCCAACCGCGCCGCCGGCAACG<br>GCTACTGGAAGGCC<br>ACCGGCGCCGACAAGCCCGTCGC<br>GCCGCGGGGGCGCACGCTCGGGA<br>TCAAGAAGGCGCTC<br>GTCTTCTACGCCGGGAAGGCGCC<br>GCGTGGGGTCAAGACGGACTGGA<br>TCATGCACGAGTAC<br>AGGCTCGCGGACGCCGGCCGCGC<br>CGCCGCCGCCAAGAAGGGATCGC<br>TCAGGCTGGATGAC<br>TGGGTGCTGTGCCGCCTGTACAAC<br>AAGAAGAACGAGTGGGAGAAGAT<br>GCAGCTGGGGAAG	MGLPVRRERDAEAELNLPP<br>GFRFHPTDDELVEHYLCRK<br>AAGQRLPVPIIAEVDLYKFD<br>PW<br>DLPERALFGVREWYFFTPR<br>DRKYPNGSRPNRAAGNGY<br>WKATGADKPVAPRGRTLGI<br>KKAL<br>VFYAGKAPRGVKTDWIMH<br>EYRLADAGRAAAAKKGSL<br>RLDDWVLCRLYNKKNEWE<br>KMQLGK<br>ESAAGVGTAKEEAMDMTT<br>SHSHSHSQSHSHSHSWGET<br>RTPESEIVDNDPFPELDSFPA<br>FQ<br>DPAAAMMMVPKKEQVDD<br>GSASANAGKSSNLFVDLSY<br>DDIQGMYSGLDMLPPPGED<br>FYSLF<br>ASPRVKGNQPAGAAGLGPF

Sspon.01G0059240-1D	*SsNAC017*	ATGGGAACCATGACTCTGCCGCC<br>CGGGTTCCGCTTCCACCCGACGG<br>ACGACGAGCTGGTG<br>GGCTACTACCTCAAGAGGAAGGT<br>GGACAACCTCAAGATCGAGCTCG<br>AGGTCATCCCCGTC<br>ATTGATCTCTACAAATCCGAGCCA<br>TGGGAACTACCAGAGAAGTCTTT<br>CCTGCCGAAAAGA<br>GACCTGGAATGGTTCTTCTTCTGC<br>CCCCGCGACCGCAAGTACCCCAA<br>CGGTTCCCGCACC<br>AACCGCGCCACGACGACGGGGTA<br>CTGGAAGGCCACGGGCAAGGACC<br>GCCGCATTGCCTGC<br>GACGGCGGCGTCTACGGCCTCCG<br>CAAGACGCTCGTCTTCTACCGCGG<br>TCGCGCCCCCGGC<br>GGCGAGCGCACCGACTGGGTCAT<br>GCACGAGTACCGCCTCTGCCAGG<br>ACCTCGCCCACGGC<br>GCATGCAACTTCATCGGTGCTTAC<br>GCGCTGTGCCGCGTGATCAAGCG<br>GCACGAGGCCGGG<br>CTGCTGCAGGGCGAGCCGGCGGC<br>GGGCAGGGCGAAAGCAGCCGCCG<br>GCAGCAGCGCGAGA	MGTMTLPPGFRFHPTDDEL<br>VGYYLKRKVDNLKIELEVIP<br>VIDLYKSEPWELPEKSFLPK<br>R<br>DLEWFFFCPRDRKYPNGSR<br>TNRATTTGYWKATGKDRRI<br>ACDGGVYGLRKTLVFYRG<br>RAPG<br>GERTDWVMHEYRLCQDLA<br>HGACNFIGAYALCRVIKRH<br>EAGLLQGEPAAGRAKAAA<br>GSSAR<br>GQMSKVSSSSSLVSSEQLSA<br>SFAPTNSTSPPPTLDVGSRGI<br>MCTMAESSNAFQSPVGVGY<br>GVTATATTGTGGLPSSPLPP<br>PLLLPSPHDTFFIGDDFPAAA<br>ADESRSHAHLFGGDIMRMG<br>GGVSEHELKWDSFLSCPNT<br>FSS

Sspon.01G0063470-1D	*SsNAC018*	ATGGTGATCATCAGCCCGGCCGT<br>CGACGACGCCGATCCCCTCGCGG<br>CGGCCAATGGCGCC<br>CGCGCTCCTCGACCGGCGCCGGC<br>GGCAAACAACTTCGACCCTGCTG<br>CTGCATTGTCGCTC<br>TTCGACGTCGTCCACGGGACGAC<br>GGCGACGGCAGACGTCGGTGCAG<br>GCACTACGCCCTGG<br>TGGAGCTCTCCCCCGTCGTATTAT<br>GCGGCGTCGACGACAGCCACCAG<br>CTCCGGCGCGTAC<br>GACGCCTCCTCGCCGTTCCAAAGC<br>CATGGCGACGATCTCGCCTGCTAC<br>GCCGGGTCCATT<br>ATGGACACCTTGTTCCTGCAGCCG<br>CCGGTGCACATGCAGCCGCCGTC<br>GATGGCGGAGCTA<br>TCCTACTTCTACTTCCCGCAGCTG<br>CAGGGGTTCCCGCCATGCTTCGCC<br>TCACCTTCCGCT<br>ATTGAACCTAGTACCATACGGCC<br>GGAACTACAGTTGCCGACAACGA<br>TGATGCAGCCGGCG<br>AGCGTCCCGCCGCCGCCGTACCC<br>GCAAACCCAAGGGACAACGACCG<br>CCCCTGCACTTGCA	MVIISPAVDDADPLAAANG<br>ARAPRPAPAANNFDPAAAL<br>SLFDVVHGTTATADVGAGT<br>TPW<br>WSSPPSYYAASTTATSSGA<br>YDASSPFQSHGDDLACYAG<br>SIMDTLFLQPPVHMQPPSM<br>AEL<br>SYFYFPQLQGFPPCFASPSAI<br>EPSTIRPELQLPTTMMQPAS<br>VPPPPYPQTQGTTTAPALA<br>DTQQAQEDDTRPAPRRRGR<br>PSKRALAVSEPPPVSKPTKR<br>VAVGRNRAASSSQKTSSAA<br>TG<br>WATVSSAGHVPDQAGATSS<br>TGSTSGQAAACTSTKSPMA<br>LLCEERCQLQPDFSNGASTG<br>VV<br>EGHQQAAIAPSEAMVTGPQ<br>YADTSAVGVRFHPTDQQLI<br>GFLRMKYAGQHMPVKFFK<br>EFDV<br>YQAHPMAIKGACGESQDGC<br>WYAFSPRDRKYRNGKRPAR<br>SVFAEGGGEQVGFWKSNSR<br>LGD

Sspon.02G0005070-1A	*SsNAC019*	ATGAGGTCCATGCAGAGCACGGA<br>CTCGTCGTCCGGCGAGTTGCCACC<br>GCAGAGGCAGCCG<br>AGCTCGGCGCCGGATCTCCCGCC<br>GGGGTTCCGGTTCCACCCGACGG<br>ACGAGGAGCTGGTC<br>GTCCACTACCTCAAGAAGAAGGC<br>CGCGTCCGTGCCGCTCCCCGTCGC<br>CATCATCGCCGAG<br>GTCGACCTTTACAAGTTCGATCCG<br>TGGGAGCTACCTGATAAGGCGAC<br>CTTCGGGGAGCAG<br>GAGTGGTACTTCTTCAGCCCGAG<br>GGACCGCAAGTACCCCAACGGAG<br>CGCGGCCGAACAGG<br>GCGGCAACGTCCGGCTACTGGAA<br>GGCGACTGGCACAGACAAGCCCA<br>TCATGGCGTCGTCC<br>GGCGGCAACCGCGAGAAGGTCGG<br>CGTTAAGAAGGCGCTCGTGTTCTA<br>CCGCGGGAAGCCA<br>CCAAAGGGCATCAAGACCAACTG<br>GATCATGCACGAGTACCGCCTCG<br>CGGATGCGGCGAGC<br>TCAACCACCAGCCGGCCGCCGCC<br>TTGCAACGTCGTCGGAGGCAAGG<br>CCACATCGTCTCTC	MRSMQSTDSSSGELPPQRQ<br>PSSAPDLPPGFRFHPTDEEL<br>VVHYLKKKAASVPLPVAIIA<br>E<br>VDLYKFDPWELPDKATFGE<br>QEWYFFSPRDRKYPNGARP<br>NRAATSGYWKATGTDKPI<br>MASS<br>GGNREKVGVKKALVFYRG<br>KPPKGIKTNWIMHEYRLAD<br>AASSTTSRPPPCNVVGGKA<br>TSSL<br>RLDDWVLCRIYKKINKLGA<br>GERSMECEDSVEDAVAAYP<br>THAAAAMAAVAGGGRAAH<br>HNGN<br>NYTSLIHHHHEDNFLDGFIT<br>TEDTAGLSAGASSLSQLAA<br>AARAAAPADTKQLLVPSSS<br>TT<br>PFNWLDASTIAILPPAKRFH<br>GYNRDTTDGGGTSLSSPSER<br>NNLAAAAGAVDSGACSGG<br>TN<br>AIVPTFLNPLGVQGATAYH<br>HHAIFGTPVTPEAAAAAAT<br>ATCGFQHPYQLSGVNWNP

Sspon.02G0008330-1A	*SsNAC020*	ATGGCGCCGGTGGGTCTCCCGCC<br>GGGCTTCCGGTTCCACCCGACGG<br>ACGAGGAGCTGGTG<br>AACTACTACCTGAAGCGCAAGAT<br>CCACGGGCTGAAGATCGAGCTGG<br>ACATCATCCCCGAG<br>GTCGACCTCTACAAGTGCGAGCC<br>ATGGGAGCTCGCAGACAAGTCGT<br>TCCTGCCTAGCCGT<br>GACCCGGAATGGTACTTCTTCGG<br>GCCGCGGGACCGCAAGTACCCCA<br>ACGGGTTCCGCACC<br>AACCGCGCGACGCGGGCGGGGTA<br>CTGGAAGTCCACGGGCAAGGACC<br>GCCGCGTGCTGCAC<br>CACGGCGGCCGCCCCATCGGCAT<br>GAAGAAGACGCTCGTCTACTACC<br>GCGGCCGCGCGCCG<br>CAGGGGGTCCGCACCGACTGGGT<br>CATGCACGAGTACCGCCTCGACG<br>ACAAGGACGCCGAG<br>GACACGCTACCCATCCAGGACAC<br>ATATGCATTATGCCGAGTCTTCAA<br>GAAGAACGCCATC<br>TGCACCGAGGTGGACGGCCTGCA<br>AGCGCAGTGCAGCATGGCGCTGC<br>TGGAGGGCGCCTGC	MAPVGLPPGFRFHPTDEEL<br>VNYYLKRKIHGLKIELDIIPE<br>VDLYKCEPWELADKSFLPS<br>R<br>DPEWYFFGPRDRKYPNGFR<br>TNRATRAGYWKSTGKDRR<br>VLHHGGRPIGMKKTLVYYR<br>GRAP<br>QGVRTDWVMHEYRLDDKD<br>AEDTLPIQDTYALCRVFKK<br>NAICTEVDGLQAQCSMALL<br>EGAC<br>RQLLTSGSQEYQTPSPDVPV<br>GSTSGGADDDADKDESWM<br>QFISDDAWCSSTADGAEEST<br>SS<br>SWEAMAPADLPPGFRFHPT<br>DEELVNYYLKRKVHGLSIE<br>LDIIPEVDLYKCEPWELAGN<br>LK<br>SKDRKYPNGCRTNRATQAG<br>YWKSTGKDRRINYQNRSIG<br>MKKTLVYYKGRAPQGLRT<br>NWVM<br>HEYRIEESECENTMGIQYSR<br>SSSLISHRRAEDRERETYTH<br>VVTMAPVGLPPGFRFHPTD

Sspon.02G0008350-1A	*SsNAC021*	ATGGCGCCGGTGGGTCTCCCGCC GGGCTTCCGGTTCCACCCGACGG ACGAGGAGCTGGTG AACTACTACCTGAAGCGCAAGAT CCACGGGCTGAAGATCGAGCTGG ACATCATCCCCGAG GTCGACCTCTACAAGTGCGAGCC ATGGGAGCTCGCAGACAAGTCGT TCCTGCCTAGCCGT GACCCGGAATGGTACTTCTTCGG GCCGCGGGACCGCAAGTACCCCA ACGGGTTCCGCACC AACCGCGCGACGCGGGCGGGGTA CTGGAAGTCCACGGGCAAGGACC GCCGCGTGCTGCAC CACGGCGGCCGCCCCATCGGCAT GAAGAAGACGCTCGTCTACTACC GCGCCGCGCGCCGC AGGGGGTCCGCACCGACTGGGTC ATGCACGAGTACCGCCTCGACGA CAAGGACGCCGAGG ACACGCTACCCATCCAGAAATTTT	MAPVGLPPGFRFHPTDEEL VNYYLKRKIHGLKIELDIIPE VDLYKCEPWELADKSFLPS R DPEWYFFGPRDRKYPNGFR TNRATRAGYWKSTGKDRR VLHHGGRPIGMKKTLVYYR AARR RGSAPTGSCTSTASTTRTPR TRYPSRNFLDALLSRVSTV

Sspon.02G0010630-1A	*SsNAC022*	ATGGAGAGGCCTGCGCAGGCGCC<br>CACGCAGCTGCCGCCGGGGTTCC<br>GGTTTCACCCCACC<br>GACGAGGAGCTGGTACTGCTGTA<br>CCTCCGCCGGAAGGCCCTGGCTC<br>GCCCGCTGCCGTCC<br>GCCGTCATCCCCGTCGTCCACGAC<br>GTCGCCGGGCTCGATCCATGGGA<br>CCTCCCTGGAGCA<br>AGCGAAGGGGAGGGCTACTTCTT<br>TAGCCTGCGGCGAGCGCCGGCGA<br>CCGGGCGTGGCAGC<br>CGCAGGAGGAGAGCTGGGAGCGG<br>GTACTGGAAGGCCATGGGGAAGG<br>AGAAGCCGGTGTTC<br>CTGCAGTGCGGCGGCGGCGTAGG<br>CTACAAGAGGCAGCTGCTCGTGG<br>GCGTGAAGACGGCG<br>CTCGTCTTCCACCGCAGTGAGCCG<br>GCGCCGTCGTCGCGGACAGGCTG<br>GATCATGCACGAG<br>TACCGGCTCGCCGTGCCCCGCGG<br>CGTGGCCGAGCAGAGGGGGAAGA<br>ATGCGAGCCAGGGT<br>TGCGTTGCTGAGCCGGCGGGAGA<br>GTGGGTCGTGTGCCGGGTCTTCCT<br>GAAGAACAACAGG	MERPAQAPTQLPPGFRFHPT<br>DEELVLLYLRRKALARPLPS<br>AVIPVVHDVAGLDPWDLPG<br>A<br>SEGEGYFFSLRRAPATGRGS<br>RRRRAGSGYWKAMGKEKP<br>VFLQCGGGVGYKRQLLVG<br>VKTA<br>LVFHRSEPAPSSRTGWIMHE<br>YRLAVPRGVAEQRGKNASQ<br>GCVAEPAGEWVVCRVFLK<br>NNR<br>PGSSRPNRVASSKTLGHRAS<br>AGPPQHREDVGGRQQPLLF<br>SAPQSSSSSCVTGVIDSSDED<br>HEVRSGGMTRDAPAAPQRE<br>AY

Sspon.02G0011250-1A	*SsNAC023*	ATGGAAGGGTCAGCAGCAGGAGG<br>TGGTGGAGGGGGAAGGGGAGGG<br>GGAGAGTCGAAGAAG<br>AAGGAGGAGGAGAGCTTGCCGCC<br>GGGCTTCAGGTTCCACCCGACGG<br>ACGAGGAGCTGATC<br>ACGTACTACCTGCGGCAGAAGAT<br>CGCCGACGGCAGCTTCACGGCGA<br>GGGCCATCGCCGAG<br>GTCGACCTCAACAAGTGCGAACC<br>GTGGGATCTCCCGGAGAAAGCGA<br>AGCTGGGAGAAAAA<br>GAGTGGTATTTCTTCAGCCTAAGG<br>GACCGGAAGTACCCAACAGGCGT<br>TCGAACAAACCGT<br>GCCACTAACGCTGGGTATTGGAA<br>GACAACGGGGAAAGATAAGGAA<br>ATCTACACCGGTCAG<br>CTACCAGCCACGCCAGAGCTAGT<br>AGGGATGAAGAAAACCCTGGATG<br>AGTGGGTGGTGTGC<br>CGGGTGTTCGCCAAGAGCGCCGG<br>CGCCAAGAAGTACCCGTCCAACA<br>ACGCGCACTCGCGG<br>TCGCACCACCACCACCACCCGTAC<br>GCGCTGGACATGGTGCCGCCCCT<br>CCTGCCCACGCTG	MEGSAAGGGGGGRGGGES<br>KKKEEESLPPGFRFHPTDEE<br>LITYYLRQKIADGSFTARAI<br>AE<br>VDLNKCEPWDLPEKAKLGE<br>KEWYFFSLRDRKYPTGVRT<br>NRATNAGYWKTTGKDKEI<br>YTGQ<br>LPATPELVGMKKTLDEWV<br>VCRVFAKSAGAKKYPSNNA<br>HSRSHHHHHPYALDMVPPL<br>LPTL<br>LQHDPFARHYHHHPHPYMT<br>PADLAELARFARGTPGLHP<br>HIQPHPGTSAAAYMNPAAA<br>AVA<br>APPSFTLSGGGLNLNLGASP<br>AMPSPPPPPQALHAMSMAM<br>GGQTGNHHQVMAGEHQQQ<br>QMA<br>TAAGLGGCVIVPGADGAFG<br>ADAAGGRYQSLDVEQLVER<br>YWPVGYQV

Sspon.02G0011260-1A	*SsNAC024*	ATGGAGACCCCGCCGCCGCGCCG<br>GTGGCCGCCGGGCTTCCGCTTCAG<br>CCCCACCGACGAG<br>GAGCTTGTTCTCTACTTCCTCAAG<br>CGCCGGATCGCCTCCGGCCGCCCC<br>TCTCCCTACGTC<br>GCCGACGTCGAAGTGTACAAGTC<br>ACACCCCTCCCACCTCCCCGAGAG<br>GTCGGCGCTGCAG<br>ACGGGGGACAGGCAGTGGTTCTT<br>CTTCAGCCGGATGGACCGCAAGT<br>ACCCCAACGGCTCG<br>CGCGCCAGCCGCACCACTGGCGA<br>CGGCTACTGGAAGGCCACGGGGA<br>AGGACCGCTTCATC<br>TGCGGCGGCGGCCGGGCGGTGGG<br>CAACAAGAAGACGCTGGTGTACC<br>ACCACGGCCGGGCC<br>CCGCGCGGGGAGCGCACGGATTG<br>GGTCATGCACGAGTATACCCTACT<br>CGCCGACGCGCTC<br>CCGCCGGCCGCGCAGGGGAGGGA<br>GTTCTACGCGCTGTACAAGCTCTT<br>CCAGAAGAGCGGC<br>GCCGGGCCCAAGAACGGCGAACA<br>GTACGGTGCTCCCTTTCGGGAGG<br>AGGATTGGTTGGAT	METPPPRRWPPGFRFSPTDE<br>ELVLYFLKRRIASGRPSPYV<br>ADVEVYKSHPSHLPERSAL<br>Q<br>TGDRQWFFFSRMDRKYPN<br>GSRASRTTGDGYWKATGK<br>DRFICGGGRAVGNKKTLVY<br>HHGRA<br>PRGERTDWVMHEYTLLAD<br>ALPPAAQGREFYALYKLFQ<br>KSGAGPKNGEQYGAPFREE<br>DWLD<br>DDDEGVTADAAANSVPNTS<br>NPPSTVEEHAITDRELPIEDL<br>DELLSNFGNDQEGFSEAQP<br>A<br>SSQGWLSEGGDKAQVVDA<br>SISNGAVVVAENTCTDLPLG<br>DIEQLLMQISDDQQNAELFS<br>DL<br>STSVPELQFQCDDGQVWLD<br>ADGGHEVCAADPTASSGAV<br>VTAECTDTELPLGDLEGLLL<br>QI<br>ANDQDMVEPQSDLSAPIPH<br>HNFNQAGIGDLHESHGAPV<br>GNLSCIVQESTFCEPRTEPSS

Sspon.02G0013400-1A	*SsNAC025*	ATGGCGGGAGACGCCGAGAAGGA<br>GGAGGAGTACCCGATCGGGTTCC<br>GGTTCAAGCCCAAG<br>GACGAGGAACTCGTCGAGTACTA<br>TCTCGTCCCCAGGCTGAGGCGCC<br>AGCCGACGGTGCCC<br>AACGACTGCATCACCGAGTGCGA<br>CGTCTACTCGTGCCACCCGGACAC<br>GCTCACCAAAGAG<br>CACAAGGGCGTGGACCAGGAGGA<br>GTGGTACTTCCTGTCGCCGAGGTC<br>TCGCATGTACGGG<br>AACGGCGTGAGGCCGGCGCGCAA<br>GACGCGGGACGGCCGCGGCCGGT<br>GGAAGGCGTCCACG<br>GCCTCCAAGGAGGTGGACCAGAA<br>GGTGGTCTGCAACGGCATCACCTT<br>CTGCCGGAGCGTG<br>CTCAACTACTTCGAGGGCGTCCCC<br>AAGAAGGAGGTGCGCACCAAGTG<br>GATCATGCTCGAG<br>CTCAAGGTCCCGTGCTTCGAGATC<br>AAGCTCGACAAGGCCGGCCCAAA<br>GAACATGTTGGAC<br>GAGTACGTCGTGTGCAAGATCTA<br>CGTGTCCCCACAGCACAAAAAGA<br>AGGGTGACGCCGAC	MAGDAEKEEEYPIGFRFKP<br>KDEELVEYYLVPRLRRQPT<br>VPNDCITECDVYSCHPDTLT<br>KE<br>HKGVDQEEWYFLSPRSRM<br>YGNGVRPARKTRDGRGRW<br>KASTASKEVDQKVVCNGIT<br>FCRSV<br>LNYFEGVPKKEVRTKWIML<br>ELKVPCFEIKLDKAGPKNM<br>LDEYVVCKIYVSPQHKKKG<br>DAD<br>EEGTSSACEGDDGEEQACA<br>PTQHVQGMAGSMLSEKQA<br>SKRPMLDKVRPGTLGIAST<br>QASC<br>PRTEAYYGVPGQPTGAHSS<br>QAPQMPQRQAGAFHGQSLL<br>VQQPTQQTPVLYHQSFTDA<br>NAT<br>NLGVPGNPPPKRDPGTAFRP<br>QVSLQCHYDQNYRGVKPPG<br>NASSSQPQMLAASSFLPRQP<br>P<br>PQQPLFFNGSYPYQSRALTL<br>EAAGHDGAANGRNGAARC<br>LNVNAEQYFVDLAMIMSNP

Sspon.02G0014720-1T	*SsNAC026*	ATGGATGAGAGAGCTGATATGGA CAAGTCTGATGAGGTTCTTCTTCC AGGTTTCCGGTTC CATCCTACAGATGAAGAGCTCAT AAGCTTTTATCTCAAGAGAAAGA TTCAGCAAAAACCT GTGTCCATTGAGCTCATTAGGCAG CTGGACATCTACAAGTATGATCC ATGGGATCTCCCA AAGCTTGCGAGCACCGGGGAGAA GGAATGGTACTTTTACTGCCCAAG GGACCGGAAGTAT CGGAATAGTGCTAGACCAAATAG AGTCACAGGAGCTGGATTCTGGA AAGCAACAGGAACA GATAGGCCAGTCTACTCTTATGAA GGGACCAAGTGCATAGGCCTGAA GAAGTCCCTTGTC TTTTACAAAGGGAGAGCAGCGAG AGGAATCAAGACTGACTGGATGA TGCATGAGTACAGG CTTCCTTCACTCAATGACCCCTCA CGTCCTAAAACACCAAGAGACAA AAATATTCCAGCC AATGATGCATGGGCTATTTGCAG GATATTTAAAAAGCCTAATTCTGT GGCACAAAGAGTG	MDERADMDKSDEVLLPGF RFHPTDEELISFYLKRKIQQ KPVSIELIRQLDIYKYDPWD LP KLASTGEKEWYFYCPRDRK YRNSARPNRVTGAGFWKA TGTDRPVYSYEGTKCIGLK KSLV FYKGRAARGIKTDWMMHE YRLPSLNDPSRPKTPRDKNI PANDAWAICRIFKKPNSVA QRV LSHSWGPQSITTTEPELLSA LQSIQASHFALESSTCSANR FNSQQCLQGQQQQNLNNSQ D RSPCKVITFNRSPSLPSEKDI HSSSVILPLEIQSQQKSSDVT SVMLSMAPGILNSMNEAI PNTESGLLGPSNGYMVDW ATDSSGGIGNSDGDAYTRK SGNGYSSGNECGIPGKIKFP FNL G

Sspon.02G0015260-1A	*SsNAC027*	ATGGCGGAGCAGCAGCAGCCGAAGCAGGTGGAGATGAACATCGTTGGCGCTAGTGGAGGCCTCAGCCTGCCTCCAGGGTTTCGCTTCCACCCGAGTGACAATGAGATTGTCAGCATCTACCTCACGAACAAGGTGCGTAATAGGGACCTCACCAGCACTGTCATCACAGAGGTCGATCTAAACAAGACTGAGCCATGGGACCTCCCACGAGAGGCAAAACTAGGCGAGAAAGAGTGGTACTTCTTCTACCAGAAGGACCGCAAGTATCAGACAGGGCTAAGGGCGAACCGGGCCACAAAGGGCGGTTACTGGAAAGCAACCGGCAAGGACAAGGAGGTCTACAATACCACACAAGGGGTGGTGTTGCTTGTCGGCATGAAGAAGACGCTCGTCTTCTACAAGGGCAGGGCTCCCAGGGGCGACAAAACGAACTGGGTGATGCACGAGTACAGGCTCGAAGGTAGCGGTAGGCTCCCCGACCCGGCATCCGCATCCAGCTCAGATGAGTGGGTGGTTTGTCGTGTGTTCGACAAGACCACT	MAEQQQPKQVEMNIVGASGGLSLPPGFRFHPSDNEIVSIYLTNKVRNRDLTSTVITEVDLNKTEPWDLPREAKLGEKEWYFFYQKDRKYQTGLRANRATKGGYWKATGKDKEVYNTTQGVVLLVGMKKTLVFYKGRAPRGDKTNWVMHEYRLEGSGRLPDPASASSSDEWVVCRVFDKTTRIEKMTTPTYKVSMAGAEIGQNQNNIPTIPIPMPLQPPLLVPMPMESPILSDFATDPMATYFPNTGTGMAPMMPSMAGIGGTSRLQINDALFGNLIATPPQTNFYHQMGTGAATGHMGMGAAGHMDMGAAGTDGFDVDAPRPSSMASQKDEQANAAEIWSMMSVAGPESATPTIEMDGIWKY

Sspon.02G0020600-1A	*SsNAC028*	ATGGCAATGGTGGCTGCGGCGGA<br>GGGGAGCGGGCGGAGCCGGAGG<br>GACGCCGAGGCGGAG<br>CTCAACCTGCCGCCGGGGTTCCG<br>GTTCCACCCCACCGACGAGGAGC<br>TCGTGGTGTACTAC<br>CTCTGCAGGAAGGTGGCGCGGCA<br>GCAGCTGCCCGTGCCCATCATCGC<br>CGAGGTCGATCTT<br>TACAAGTTCGATCCGTGGGATCTG<br>CCCGAGAAGGCGCTGTTCGGCCG<br>CAAGGAGTGGTAC<br>TTCTTCACGCCGCGGGACCGCAA<br>GTACCCGAACGGCTCGCGCCCCA<br>ACCGCGCCGCCGGG<br>AGGGGGTACTGGAAGGCCACGGG<br>GGCTGACAAGCCGATCGCGCCCA<br>AGGGCAGCGGCAGG<br>GTGGCGGGGATCAAGAAGGCGCT<br>TGTGTTCTACTCCGGCAAGGCGCC<br>CAGGGGCGTCAAG<br>ACAGACTGGATCATGCACGAGTA<br>CCGCCTCGCCGACGCGGACCGAG	MAMVAAAEGSGRSRRDAE<br>AELNLPPGFRFHPTDEELVV<br>YYLCRKVARQQLPVPIIAEV<br>DL<br>YKFDPWDLPEKALFGRKE<br>WYFFTPRDRKYPNGSRPNR<br>AAGRGYWKATGADKPIAP<br>KGSGR<br>VAGIKKALVFYSGKAPRGV<br>KTDWIMHEYRLADADRAPS<br>KKGSQKP

Sspon.02G0027090-1A	*SsNAC029*	ATGGAGGTGAAGGATGCTGCGAA<br>GCCGAGGCTGCCGCCGGGATTCA<br>GGTTCCGGCCCACG<br>GACGAGGAGCTGATCGTTCACTA<br>CCTCCGGCGCCGCGCGCTGGCCTC<br>CCCGCTGCCGCCC<br>GCCGTCGACATCCCGGACGTCCG<br>CATCCTGGCGCACGACCCTTCCGA<br>CCTGCTGCCCCCA<br>GGGTTCAGCGAGCAGGAGCGCTA<br>CTTCTTCACGTGCAAGGAGGCCA<br>AGTACGTGAAGGGC<br>CGGCGCGCGAACCGCGCCACGGG<br>GGCCGGGTACTGGAAGGCCACGG<br>GCAAGGAGAAGCCG<br>GTGGCCGTGGCTATCCCGGCGGC<br>GGCTCGGGGCGGCCAGGGCCAGG<br>CCGTGCTTGTGGGC<br>ATGAAGCGGTCGCTGGTGTTCTAC<br>CGGGGAAAGCCCCCGACGGGGAG<br>CAAGACGGACTGG<br>GTGATGCACGAGTACCGGCTCGC<br>GGGCGCCGGGCTCGCCCCGTGCC<br>GCCGCGCGGCAGCG<br>CAGGACGGCGACGCCGACGCGGC<br>AGTGTCGCGGCCCGCCGAGGGCT<br>GGGTGCTGTGCCGC	MEVKDAAKPRLPPGFRFRP<br>TDEELIVHYLRRRALASPLP<br>PAVDIPDVRILAHDPSDLLPP<br>GFSEQERYFFTCKEAKYVK<br>GRRANRATGAGYWKATGK<br>EKPVAVAIPAAARGGQGQA<br>VLVG<br>MKRSLVFYRGKPPTGSKTD<br>WVMHEYRLAGAGLAPCRR<br>AAAQDGDADAAVSRPAEG<br>WVLCR<br>VFRKKKGSAAASPGEDRSD<br>GESESAGAGGPGFIDFFARA<br>DAAGRRRRAASPVVSSSCL<br>TD<br>ASPERQQGREQETTSRGA

Sspon.02G0031110-1A	*SsNAC030*	ATGGTGGAGGCGGAGCTGCCACC<br>GGGGTTCCGGTTCCACCCGAGGG<br>ACGACGAGCTCATC<br>TGCGACTACCTGGCGCCCAAGCT<br>CGGCGGCAAGGTCGGCTTCTCCG<br>GCCGCCGGCCACCC<br>ATGGTCGACGTCGACCTCAACAA<br>GGTCGAGCCATGGGATCTCCCCG<br>GTGAGGAGTGGTAT<br>TTCTTCAGCCTCAAGGACCGGAA<br>GTACGCGACAGGGCAGCGGACGA<br>ACCGGGCTACAGTG<br>TCGGGCTATTGGAAGGCGACGGG<br>GAAGGACCGGGTGGTGGCGCGGC<br>GAGGGGCGATGGTG<br>GGGATGAGGAAGACGCTGGTGTT<br>CTACCAGGGGAGGGCGCCCAAGG<br>GCAGGAAGACCGAG<br>TGGGTGATGCATGAGTATAGGAT<br>GGAGCCTGCTCTTGACCAATCCTC<br>CTCCTCCAACTTC<br>TCCTCCAACTCCAAGGATGAAGA<br>TTGGGTGCTGTGCAGAGTCATCTG<br>CAAGAAGAAACTA<br>GCAGGAGGAGGAGGCAGCTCCAA<br>GGCATCAAGGAGCCTGGCCAGCA<br>ATGGCGGCCGTGAG	MVEAELPPGFRFHPRDDELI<br>CDYLAPKLGGKVGFSGRRP<br>PMVDVDLNKVEPWDLPGE<br>EWY<br>FFSLKDRKYATGQRTNRAT<br>VSGYWKATGKDRVVARRG<br>AMVGMRKTLVFYQGRAPK<br>GRKTE<br>WVMHEYRMEPALDQSSSS<br>NFSSNSKDEDWVLCRVICK<br>KKLAGGGGSSKASRSLASN<br>GGRE<br>TAPTTSPPLPPLMDTTLAQL<br>QAAMNTTATAGALEQVPCF<br>SSFNNNIASNSNGNAAAAQ<br>PC<br>YLPMATGGSHGTSYLDHGL<br>LPELGGCFDPLNSDKKLLK<br>AVLSQFGDDVVPSLQHEMA<br>AAT<br>ATSTWMYHF

Sspon.02G0032050-1A	*SsNAC031*	ATGGAGGAGGAGAAGAAGCCATA<br>TCAAAGGATCACTAAGAAGGATA<br>GCGGCAGCGGAGCA<br>GATGACGGAAATGAAGACGATGA<br>TTTGGTGTTCCCGGGCTTCCGGTT<br>CCATCCAACTGAT<br>CAAGAGCTGGTGGGGTTCTACCT<br>GAAGAGGAAAGTGGAGAAGAAG<br>CTTTTCAGTATTGAC<br>ATCATCAGGGAGATCGATATCTA<br>CAAGCATGACCCCTGGGATCTTCC<br>AAATGAAGCTCGG<br>CATGTGGTGCAGGGCTCAGGAGA<br>TAAGGACTGCTACTTCTTCTGCCT<br>GCGTGGCCGGAAG<br>TACCGGAACAGCATCCGGCCCAA<br>CCGCGTCACTGGCTCTGGCTTCTG<br>GAAGGCCACCGGC<br>ATCGACAAGCCCATACATGATGG<br>TGCTGGCGAGTGCGTCGGTCTCA<br>AGAAGTCCCTCGTC<br>TACTACCGTGGCAGCGCCGGCAG<br>GGGCACAAAGACTGACTGGATGA<br>TGCACGAGTTCCGA<br>CTCCCCTCTACGGATTTGGTTGAG<br>CTCCATGATGCTAGGAGCACGTC<br>CTACAACCGGAGG	MEEEKKPYQRITKKDSGSG<br>ADDGNEDDDLVFPGFRFHP<br>TDQELVGFYLKRKVEKKLF<br>SID<br>IIREIDIYKHDPWDLPNEARH<br>VVQGSGDKDCYFFCLRGRK<br>YRNSIRPNRVTGSGFWKAT<br>G<br>IDKPIHDGAGECVGLKKSLV<br>YYRGSAGRGTKTDWMMHE<br>FRLPSTDLVELHDARSTSYN<br>RR<br>CQQQQKQEHGSKQSHHQQ<br>QQLQYYYDYYRYHQHHQH<br>HYLHSSEAAYAATGHHQPF<br>LLRDD<br>DKDCVISIHGSSIAAVDIDIN<br>SRDSGSTWDELGRIMEI

Sspon.02G0036100-1B	*SsNAC032*	ATGGCGCGCGCTGATCTCCCACC<br>AGGCTTTAGGTTCCATCCAACTGA<br>TGAGGAGCTTGTG<br>AACTATTACCTCAAGAGGAAGGT<br>CCATGGGCTCAGCATCGAGCTCG<br>ACATAATCCCTGAA<br>GTAGACCTCTACAAATGTGAGCC<br>TTGGGAGCTAGCAGAGAAATCAT<br>TCCTGCCTAGTAGA<br>GACTCCGAGTGGTATTTCTTTGGG<br>CCAAGGGATAGGAAGTATCCAAA<br>TGGATGCCGCACA<br>AACCGCGCAACCCAAGCAGGATA<br>CTGGAAATCAACAGGCAAAGATC<br>GGCGAATCAACTAC<br>CAGAACAGATCAATTGGCATGAA<br>GAAGACATTGGTCTACTACAAGG<br>GTCGAGCTCCTCAG<br>GGGTTGAGGACCAACTGGGTGAT<br>GCATGAGTACCGCATCGAGGAAA<br>GCGAATGCGAGAAC<br>ACCATGGGGATTCAGGACTCATA<br>TGCATTGTGTCGTGTCTTCAAGAA<br>AAATGTGGCACTC<br>GGAGAGTTTCAGAAGCAAAAGCA<br>AGGCGAGTGCAGCTCATCACAAG<br>CTAAAGAAAAACAA	MARADLPPGFRFHPTDEEL<br>VNYYLKRKVHGLSIELDIIP<br>EVDLYKCEPWELAEKSFLP<br>SR<br>DSEWYFFGPRDRKYPNGCR<br>TNRATQAGYWKSTGKDRRI<br>NYQNRSIGMKKTLVYYKG<br>RAPQ<br>GLRTNWVMHEYRIEESECE<br>NTMGIQDSYALCRVFKKNV<br>ALGEFQKQKQGECSSSQAK<br>EKQ<br>EQLQVSGMLDSHLVQMSM<br>FGQDDH

Sspon.02G0037950-1B	*SsNAC033*	ATGGAAGGTGATGGTGACGGCCG ACGCGCCGCGGTCGGGCCGCCGC TGCCGCCGGGCTTC CGGTTCCGGCCGACTGACGAGGA GCTGCTCACGCATTACCTCGCTCC GAAGGTGGCCGAC GCCGGCTTCGCCCCCGCCGCGCTC CGCGAGGTCGACCTGTACAAGGC CGAGCCCTGGGAC CTGCTGCCGGCCGGAGGCGGAGA GGACGACGGCGGCGTCGGCTACT TCTTCTGCAGGAGG AGCGTCAAGTTCCCGTCGGGCCT GAGGACCAACCGCGCCACGCGGG CCGGGTACTGGAAG TCCACTGGGAAGGACAGGGTGGT GGCGGCGAGCACGGGCAGGAGCA GCCGCGGTGACGGT GACTGTCCCCTCGGGGTGAGGAA GACGCTCGTCTTCTACCGCGGCCG CGCGCCTACAGGC CACAAGACCGTCTGGGTCATGCA CGAGTACCGCCTGCTCCACGGCC ATGGATACACATCA TCCCCAGTGCATGCAACGGGAAG AGCCCAAAGTGAGTGGGTGATTT GCAGGATGTTGATG	MEGDGDGRRAAVGPPLPPG FRFRPTDEELLTHYLAPKVA DAGFAPAALREVDLYKAEP WD LLPAGGGEDDGGVGYFFCR RSVKFPSGLRTNRATRAGY WKSTGKDRVVAASTGRSSR GDG DCPLGVRKTLVFYRGRAPT GHKTVWVMHEYRLLHGHG YTSSPVHATGRAQSEWVIC RMLM KKAPGEASQPGQAEAVLHP PLDDHMQPPVDSHHQHAN CFSNIALAMAPGDTNLDGIE SML PLNRHGHEELWMNYSESTY PPVATSTSGSAQAALGVRD ELTAADSFDLLPQLLDYDE AFP LQDF

Sspon.02G0038600-1B	*SsNAC034*	ATGCTCTTATTAGGCCTCCCGGAG<br>ATCTCAAGTATGGAGGCTCCTATC<br>GGTCCTTTGGAC<br>GGCCCAATTTTGAAGCTTCCATGG<br>AAGCTCGACAGGGTACTGCGCGA<br>CGGATCCATCCTG<br>CCCAAGGGCGTGGAAGACGAGAT<br>ACCTCTCATCAAGCGTGATCTTGG<br>GGAAATCATATCC<br>ATTCTTTCAAACCTGGACGACCAC<br>CATGCTACTGCTATGAAGGTCAGT<br>TGCTGGAGAAAG<br>GAAGTGCGGGAGCTGTCCTACGA<br>TATGGAGGACTTCATCGACCAGT<br>ATGAGCACGCCACT<br>GCCGTGCCCTTGCTGGCTGGATCC<br>GTTCCTCGCCGCAAGACTATTCAG<br>AAGCGTAAGAGC<br>AAGACCAGTACTCTCTTTAGGCTC<br>CGCGAGAGGCTGAAGCAGCGCCT<br>GTGGATGGCCAAC<br>AAGATTAGGGAATTCAGCGTGCG<br>CACACAAGAGGCACTTCAACGGC<br>ACAGCATGTATAAT<br>CTTGACGTTATTGCTGTCTCTGGT<br>TCCACATACACTAGATGTGCTGAT<br>GCTTGTTCTACC	MLLLGLPEISSMEAPIGPLD<br>GPILKLPWKLDRVLRDGSIL<br>PKGVEDEIPLIKRDLGEIIS<br>ILSNLDDHHATAMKVSCWR<br>KEVRELSYDMEDFIDQYEH<br>ATAVPLLAGSVPRRKTIQKR<br>KS<br>KTSTLFRLRERLKQRLWMA<br>NKIREFSVRTQEALQRHSM<br>YNLDVIAVSGSTYTRCADA<br>CST<br>SSHSTPCGKENAYVGISAA<br>MEKLQELLMVMHDEGHRK<br>LKVVSIVGVGGIGIQDQWD<br>YVNK<br>SIDYSLLTNPTSEGMKQILD<br>LSYNNLPQNLKACILYTGLY<br>EEDTIIWKDDLVNQWIAEGF<br>IQPSEGRDMKEIASSLFDRLI<br>SRKLILPVDINKNGEVLSCV<br>VHRMVLNLVIRYKSIEENF<br>VTAIHHSQATMTLADKDSIS<br>FDLTRISELFRLRYLKLTSN<br>VTLELGNQIRGLQSLETLTI<br>DARVNAVPSDIVRLPGLLHL<br>GLPAETDLPNGIGHMTLLRT<br>FGYFDLCSNSMENVQSLSM

Sspon.03G0000160-1A	*SsNAC035*	ATGGAGGGCTGGCCGCCCGGCGCGTGGCGCCTGCAGCAGCTGCAGCAGCAGCGGGGCCCCGGCGCACCCGTCGCGGGCCTGCCCATCGGCTTCCGCTTCCGCCCCACCGACGAGGAGCTGCTGCTGCACTACCTCCGCCGCAAGGCGCTCGCCTGCCCGCTCCCCGCCGGCGTCATCCCCGACGCCGACCTCGCGCGCCTCCACCCGTGGGACCTCCTCCCCGCCGCCGGACCAGGAGCAGCCGACGCCGACGGGGAGCGCTTCTTCTTCCACCGCCCGGCGACGCGGTGCTGGCGCAAGGGCGGCGGCGCGGCCAGGGCGGCCGGCACCGGCGTCTGGAGGCCGTCCGGGAAGGAGACCCTCGTCGTCTCGCCGCGCTGCAAGCGCCCCGTCGGCACCAAGCGGACGCTCGTCTTCTGCCCCCGCCGCGGCCGCGGCGGCGCCCGCACCGACTGGGCCATGCACGAGTACCGCCTCCTCCCCGCCGGCCTCCACCTCCACGGCTGCTCCGCCGCCGCCGCCACCACCAACGCGCCTCCG	MEGWPPGAWRLQQLQQQRGPGAPVAGLPIGFRFRPTDEELLLHYLRRKALACPLPAGVIPDADLARLHPWDLLPAAGPGAADADGERFFFHRPATRCWRKGGGAARAAGTGVWRPSGKETLVVSPRCKRPVGTKRTLVFCPRRGRGGARTDWAMHEYRLLPAGLHLHGCSAAAATTNAPPPTNVSSHGAGAAADWVVCRIFRRARPAHRARLSGGEEEHEAEESPSSPSSCVTDASETVGQEDDGDEGSSSGGCSVASSN

Sspon.03G0001670-1A	*SsNAC036*	ATGGACGCACGGTCGTTCATGAG<br>CGGCGGCAGCAGCAGGCACGAGA<br>ACGGGCACGGCGGC<br>GCCGCGGCTGCCGCCGCGGCGGC<br>GGCGGCAGCGGCAGGTGGAGGTG<br>GAGGTGGAGGTGTA<br>GGCGGCGGCGACGCGCATGAGGA<br>CGACCTGGTGATGCCGGGGTTCC<br>GGTTCCATCCCACG<br>GAGGAGGAGCTCATCGAGTTCTA<br>CCTCCGCCGGAAGGTGGAGGGCA<br>AGCGCTTCAACGTC<br>GAGCTCATCACCTTCCTCGACCTC<br>TACCGCTACGACCCATGGGAGCT<br>CCCTGCAATGGCC<br>GCGATTGGGGAGAAGGAGTGGTT<br>CTTCTACGTGCCGAGGGACCGCA<br>AGTACCGCAACGGG<br>GACAGGCCCAACCGGGTGACGGC<br>GTCGGGCTACTGGAAGGCCACGG<br>GCGCCGACCGCACC<br>ATCAAGGCCGACAACAGCAGCCG<br>CCCCATCGGGCTCAAGAAGACGC<br>TCGTCTTCTACTCC<br>GGCAAGGCGCCCAAGGGCGTCCG<br>CAGCAGCTGGATCATGAACGAGT<br>ACCGACTCCCGCCC	MDARSFMSGGSSRHENGHG<br>GAAAAAAAAAAAAGGGGG<br>GGVGGGDAHEDDLVMPGF<br>RFHPT<br>EEELIEFYLRRKVEGKRFNV<br>ELITFLDLYRYDPWELPAM<br>AAIGEKEWFFYVPRDRKYR<br>NG<br>DRPNRVTASGYWKATGAD<br>RTIKADNSSRPIGLKKTLVF<br>YSGKAPKGVRSSWIMNEYR<br>LPP<br>DHTDRYHKTEISLCRVYKR<br>TGIDDGHGHHPASARSTPSR<br>GGATAAQQQDNKQASSSST<br>PT<br>PPTTPSKMMQLLHGECTSPP<br>AAAICRNNNHAAAAHSDSK<br>AAAAPRQQMPTKPYCNGY<br>HLL<br>STASSSAAAVSDQQQLVGM<br>GTAAAVSSSSYEQSSRNAN<br>YAFAASTYSLLSLVNAASM<br>GGS<br>GSAAAIDELSTLVGHVHGPP<br>AYFSHQAGGGGHGHSFLPL<br>PAPSSQQPMALGTLPMSLA

Sspon.03G0001860-1A	*SsNAC037*	ATGAGCGGCGGCGGTCAGGATCT GCAGCTGCCGCCGGGGTTCCGGT TCCACCCGACGGAC GAGGAGCTGGTGATGCACTACCT CTGCCGCCGCTGCGCCAGCCTGCC CATCGCCGTCCCC ATCATCGCCGAGATCGACCTCTAC AAGTTCGATCCATGGCAGCTCCCC AGGATGGCGCTG TACGGCGAGAAGGAGTGGTACTT CTTCTCCCCGCGGGACCGCAAGT ACCCGAACGGGTCC AGGCCCAACCGCGCCGCCGGGTC CGGCTACTGGAAGGCCACCGGCG CCGACAAGCCCGTG GGCACGCCCAAGCCGCTCGCCAT CAAGAAGGCGCTCGTCTTCTACG CCGGCAAGGCGCCC AAGGGCGAGAAAACCAACTGGAT CATGCACGAGTACCGCCTCGCCG ACGTCGACCGCTCC GCCCGCAAGAAGAACAGCCTCAG GTTGGATGACTGGGTGCTGTGCC GCATCTACAACAAG AAGGGCGGGCTGGAGAAGCCGTC GGCGGCCGCCGGCGACCACAAGC CGATGGTGTTCGCC	MSGGGQDLQLPPGFRFHPT DEELVMHYLCRRCASLPIA VPIIAEIDLYKFDPWQLPRM AL YGEKEWYFFSPRDRKYPNG SRPNRAAGSGYWKATGAD KPVGTPKPLAIKKALVFYA GKAP KGEKTNWIMHEYRLADVD RSARKKNSLRLDDWVLCRI YNKKGGLEKPSAAAGDHK PMVFA AGAVSSPPEQKPFVATPGGL PPAAFPADLAAYYDRPSDS MPRLHADSSCSEQVLSPEQL A CDREVQSQPKISEWERTFAS DPVNPAGSMLVDPVVGGH AGDPLLQDILMYWSKPF

Sspon.03G0002180-1A	*SsNAC038*	ATGGCGGCCGGCCTGCCCCCGGG<br>GTACCGCTTCTACCCGACGGAAG<br>AGGAGCTGATATCC<br>TTCTACCTCCGGAACAAGCTGGA<br>CAACCTCCGCGACGACATCGAGC<br>GCGTCATCCCCGTC<br>GTCGACGTCTACTCCGTCGACCCC<br>TGGCAGCTCCCAGGGATCCACGA<br>GACGATGCTGCGC<br>GGCGGTGGCGGCGCCGGGGAGGG<br>GGAGCCGTGGTTCTACTTCTGCCC<br>GCGGCAGCAGCGG<br>GAGGCGCGGGGCGGGCGGCCCAG<br>CCGGACGACGCCGTCCGGGTACT<br>GGAAGGCCGCGGGC<br>ACGCCCGGGGTCGTCTACTCCACC<br>GCCGATCACGACCGCCGCGCCAT<br>CGGGATGAAGAAG<br>ACCATGGTGTTCTACCGCGGCCGC<br>GCGCCGTCGGGGACCAAGACCAA<br>TTGGAAGATGAAC<br>GAGTACAGGGCGCTCCAGTACGA<br>AGCCCCCGGCCCCGGCCCCGCCG<br>CCGTCGGAGTTGGA<br>GTCGGAACTGACGGGTCCTCGTC<br>GTCGTCTAGCGCCCACCACCACG<br>CCGCCGCCGTACCC	MAAGLPPGYRFYPTEEELIS<br>FYLRNKLDNLRDDIERVIPV<br>VDVYSVDPWQLPGIHETML<br>R<br>GGGGAGEGEPWFYFCPRQQ<br>REARGGRPSRTTPSGYWKA<br>AGTPGVVYSTADHDRRAIG<br>MKK<br>TMVFYRGRAPSGTKTNWK<br>MNEYRALQYEAPGPGPAAV<br>GVGVGTDGSSSSSSAHHHA<br>AAVP<br>VPPPPNVAPQPQLQLRGEFS<br>LCRLYTKSGSLRQFDRRPLA<br>VAGGVVVVPGEDPAGPSTA<br>V<br>AASPPDDDDGSGSSSMQQP<br>QQLTEERGAKDPYGDDVVT<br>ESESAHGHDPFGDDVAILD<br>ALL<br>YWPGD

Sspon.03G0009960-1A	*SsNAC039*	ATGACATGGTGCAACAGCTTCAA CGACGTGCGCGCCGTGGAGAACA ACCTGGCCACCGCC GCGGCCGTGGCCGCCGCCAAGAA ACAGCAGCAGGCCTCCTCCCACG TCAACCTCATCAGG ACCTGCCCCTCCTGTGGCCACCGC GCGCAGTATGAACAGGTGCAGGC GGCGGCGACGATC CAGGACCTGCCGGGGCTGCCGGC CGGCGTGAAGTTCGATCCGACGG ACCAAGAGCTGCTG GAGCACCTGGAAGGGAAGGCGAG GCTGGACGCCCGCAAGCTGCACC CGCTCATCGACGAG TTCATCCCCACCATCGAGGGCGA GAACGGCATCTGCTACACGCATC CGGAGAGGCTTCCT GGTGTCGGCAAGGATGGCCTCAT CCGGCACTTCTTCCACCGCCCATC CAAGGCTTACACG ACCGGGACGCGGAAGCGGCGGAA GGTCCACACCGACGAGCAGGGCG GGGAGACGCGGTGG CACAAGACGGGCAAGACCCGTCC CGTGCTCACCAACGGCAAACTCA AGGGCTACAAGAAG	MTWCNSFNDVRAVENNLA TAAAVAAAKKQQQASSHV NLIRTCPSCGHRAQYEQVQ AAATI QDLPGLPAGVKFDPTDQEL LEHLEGKARLDARKLHPLID EFIPTIEGENGICYTHPERLP GVGKDGLIRHFFHRPSKAY TTGTRKRRKVHTDEQGGET RWHKTGKTRPVLTNGKLK GYKK ILVLYTNYGKQRKPEKTNW VMHQYHLGSDEEEKDGEL VVSKVFYQTQPRQCGSGSA TAKD AVPLAAASTATDHHHHHD GGNSMLKEAGMVDFYNPA ALIGYNQAAAPTNRAAASV HLMPN FEVHTTAGAAAFGP

Sspon.03G0011440-1A	*SsNAC040*	CCGCCCAAGCCACCGCCTCCGAC CTTCGCCGCCGGCTCCTCCTCGCC CAAGCCGCCGCCT CCGACCTTCAACAGCCACCCCTCT TACGAGGAGCTCATGGACTCCTA CCTGCGCCACCGG GTCGTCTCCGGCACCAAGGTGGG CTTCATCCACGAGGCCGACCTCTA CAGCGCCGACCCC TACCAGCTCACCCAAAATCACCT GCCGGCGACTGCGAGAAGTGGGG AGAGGGCGTGGTAC TTCTTCACGACGCTGCGCCCCAAG GGCTCCTCCAAGACCCAAATGAA GCGCAATGTGGAC ACCGGAAGGGAGGGAACCTGGGT CAGCGCCGGGGTCGTGAACAACG TGCTCTCCAACGGC CAGCTGATAGGGCGGCGTAGGGG CTTCGCCTTCCACGATAAGGTGGG CGGCAACCTTGTG AAATCGGCATGGCGCATGATGGA GCTCCGCCTCGACCCCAATGAGG GAGGCCAAGAAGAG GGCCCCTCGGACCTTCTTGCCTTG TGCAAGGTGTACCGCATCCCGAA TGCCAAGGCGGAG	PPKPPPPTFAAGSSSPKPPPP TFNSHPSYEELMDSYLRHR VVSGTKVGFIHEADLYSAD P YQLTQNHLPATARSGERAW YFFTTLRPKGSSKTQMKRN VDTGREGTWVSAGVVNNV LSNG QLIGRRRGFAFHDKVGGNL VKSAWRMMELRLDPNEGG QEEGPSDLLALCKVYRIPNA KAE DESSMAVAKADAEGPAASA VVTPGPKGADEEIGAETAA GPGRKRKAGDKDSGAETV ASSP GRQKKADGDGESADAATT G

Sspon.03G0011450-1A	*SsNAC041*	ATGCTACGATCTCCGTCGGCGGCC<br>ATCAAGCCGCCGTGTCGGGCGTT<br>CCAATCCCACCCC<br>AGCGATCTGGAGCTCGTCGACTC<br>CTACCTCCGTCCCTGGGTCGAGAC<br>GGGCGTCAAGACC<br>AGCGCGTTCATCCACGACGCCGA<br>CGTGTACGCCGCCGACCCCGCCG<br>ACCTCACCCGGCAG<br>TTCGCGCCGGCGGTGGCCGGCGA<br>CGGTGAGCGGGCGTGGTACTTCT<br>ACACCCCGCTGCGC<br>CACAAGAGCGTCCACGGGAAGCG<br>GAAGACGCGCACGGTGGCCACCG<br>GCGGCGGGTGCTGG<br>CACAACGAGGCGAAATCAAAGCC<br>CGTGTACATGGTCTCCAACGGCA<br>AGCAGCACCAGATT<br>GGGCACCGCCAGAGCTTCTCCTTC<br>GTCAAGAAGGATACCGGCGTGCG<br>GGTCCGCACGGGT<br>TGGCTCATGATAGAGCTCCGCCTC<br>CTCAAAGACGGCGAGAAACCGGA<br>GGAGGAGGATAGC<br>GCCCTCGGAAATCGCGTCCTGTGC<br>AAGGTGTACCGGAGCCCGCGCAA<br>CCCCGAGCCTAGC	MLRSPSAAIKPPCRAFQSHP<br>SDLELVDSYLRPWVETGVK<br>TSAFIHDADVYAADPADLT<br>RQ<br>FAPAVAGDGERAWYFYTPL<br>RHKSVHGKRKTRTVATGG<br>GCWHNEAKSKPVYMVSNG<br>KQHQI<br>GHRQSFSFVKKDTGVRVRT<br>GWLMIELRLLKDGEKPEEE<br>DSALGNRVLCKVYRSPRNP<br>EPS<br>GSGAAAAASPGHKAEADD<br>DESSDATLDDDYDDDDSSN<br>ASLEAASGPKRRRPDDMES<br>SEAT<br>VAAPSRHSKANDEIPGGAA<br>AAPGRSEKAEIGEDSVETSA<br>AAPPRKRKALDDESSGAAA<br>PA<br>RKKKADGSSSPGAPVSATE<br>MQCPQCGTHLVVTLKRAES<br>KSETEFAKNESAPGASGAP<br>QRG<br>ETR

Sspon.03G0017190-1A	*SsNAC042*	ATGCCTGCTGATCGTGGGACTCGC<br>TTAGTTAGGATATATTGTAAAATG<br>GAAGTACTTCGT<br>GATATGCACCTGCCACCAGGATTT<br>GGATTCCATCCTTCGGATCCTGAA<br>CTTATTTCTCAC<br>TATCTGAAGAGGAAAATACTTGG<br>CCAGAAAATTGAATATGATCTTA<br>TACCAGAGGTGGAT<br>ATATACAAGCATGAACCATGGGA<br>TTTACCTGCAAAGTGCAATCTTCC<br>AATCAAGGACAAC<br>AAGTGGCATTTCTTTGCCTCTCGT<br>GACAGGAAGTACCCTACTGGCTC<br>TAGGTCAAACAGG<br>GCAACACTCGCTGGTTACTGGAA<br>ATCAACTGGGAAGGACCGAGCCA<br>TAAAGCTGAACAAG<br>CGAACTCTAGGAACAAAGAAGAC<br>TTTAGTTTTTCATGAAGGCCGTCC<br>TCCCTCTGGCAGA<br>CGCACTGAGTGGATTATGCATGA<br>GTACTACATAGACGAGAATGAAT<br>GTAAAGTCAGCCCT<br>GATATGAAGGATGCCTTTGTCCTC<br>TGCCGTGTTACTAAAAGAAATGA<br>CTGGGCATTAGAT	MPADRGTRLVRIYCKMEVL<br>RDMHLPPGFGFHPSDPELIS<br>HYLKRKILGQKIEYDLIPEV<br>D<br>IYKHEPWDLPAKCNLPIKDN<br>KWHFFASRDRKYPTGSRSN<br>RATLAGYWKSTGKDRAIKL<br>NK<br>RTLGTKKTLVFHEGRPPSGR<br>RTEWIMHEYYIDENECKVS<br>PDMKDAFVLCRVTKRNDW<br>ALD<br>NDNEVGNRNSHLEQLDDA<br>ATSAVSTVKPEDAAASVICP<br>EESNHAATPVGSAELSNDG<br>AQA<br>AITPDSTSPNGGNDLETWLE<br>ELLDPSPSFNLVADSGSADL<br>SLTEQCAESSNPGSVAPNIG<br>PGHASPIQDGTDATDYLFID<br>DLPEDLYSMLYPGTDQFNG<br>SIFLEQAGQEGIAFPTNQAY<br>M<br>MGTDAYALPNNFENGTAN<br>AELQLDQENNKMNLSNGNI<br>DNGIIIRSRRATTSPANNSLA<br>AG

Sspon.03G0017800-1A	*SsNAC043*	ATGTCCGCGGTGGAGGCACTAGG<br>GCTCCCGCCGGGGGTTCGCTTCGA<br>CCCCACAGGCGAC<br>GAGCTCGTCGAGTACTACCTCCTG<br>CCGCGCGCCCTGGGCTGCCCGCCC<br>GCGGTCCCTGGG<br>ATCATCATCGAGGACGACGCCGC<br>CACCGCCACCACCAGCGCCCAGC<br>ACCCCTGGAAGCTG<br>CTCACGCGCCACCGCCGCACCAA<br>CGACAGCGAGGCCTACTTCTTCG<br>AGAGGATCGCCGGC<br>ACCGACGACGCCACCAAAGGCGC<br>TCGTCAGGACCGGAGCTGTGGCG<br>GCGGGCGCTGGACG<br>TGGGTGGGGCAGAAGCGCGCGCC<br>GGACGAGGCGCTCCCCCCGCGCG<br>GCGAAGGCGAGCAC<br>GTCTCGTGGGGCAAGTACTCGCTC<br>AACCTGCAGGAAGGCCGCCGCAA<br>GGGCGGCAGCACG<br>GGGTGGGTCATGCACGAGTACAC<br>CGTCGCCTCGCCGCAGTGCCCGTT<br>GCTCCCCGTCAAG<br>CTCTGCCATGTCTCCTTCACCGGC<br>CACGGCCAGAAGCGCCAGCGCGT<br>GCCCGACGACGAC	MSAVEALGLPPGVRFDPTG<br>DELVEYYLLPRALGCPPAVP<br>GIIIEDDAATATTSAQHPWK<br>L<br>LTRHRRTNDSEAYFFERIAG<br>TDDATKGARQDRSCGGGR<br>WTWVGQKRAPDEALPPRG<br>EGEH<br>VSWGKYSLNLQEGRRKGG<br>STGWVMHEYTVASPQCPLL<br>PVKLCHVSFTGHGQKRQRV<br>PDDD<br>DGEGEGQELEPQAAKSAPQ<br>HKRAATGSSMVTTAMPNQ<br>ELGEDQEHRFSSYNPSSIGH<br>FWS<br>SDAGFTTLPSYDSSRLP

Sspon.03G0018260-1A	*SsNAC044*	ATGGCAGACCAGCAGCAGGAGATGAACATGGTCCGTGCCGGCGGCCTCGACCTACCTCCAGGGTTCCGCTTCCACCCAAGTGATGTTGAGATTGTCAGTGACTACCTGATGAACAAGGTGCGCAACACGAACTTCACCTGCATCGCCATCGGAGAGGCCGACATAAACAAGACTGAGCCATGGGACCTTCGGGATAAAGCAAAATGGGGCGAGAAAGAGTGGTACTTCTTCTACCAGAAGGACCGGAAGTACCCGACGGGGTTGAGGGCGAACCGAGCCACAGTGGGTGGTTATTGGAAAGCCACAGGCAAAGATAAGGAGGTCTACAAAACCATAGAAGGGGTGGTGTTGTTGGTCGGTATGAAGAAGACGCTCGTCTTCTACAAGGGCAGGGCTCCCAAGGGCGATAAAACCAACTGGGTGATGCATGAGTACAGGCTCGAAGGCAGCGGCAGGCTCCTCGTCCCCACATCCGCATCTAGCTCAGCTGCCAACACCGCCACAACCATGAAAGCTTCAACTTCTGCTTTCAAGGATGAG	MADQQQEMNMVRAGGLDLPPGFRFHPSDVEIVSDYLMNKVRNTNFTCIAIGEADINKTEPWDLRDKAKWGEKEWYFFYQKDRKYPTGLRANRATVGGYWKATGKDKEVYKTIEGVVLLVGMKKTLVFYKGRAPKGDKTNWVMHEYRLEGSGRLLVPTSASSSAANTATTMKASTSAFKDEWVVCRVFEKTIGIKKMTTPAYQVAMASAEIDQIQNNIPAIPDPMPLQLPLPVSMPMQFPILPDFSMDPVAPYYPNAGAGMPTMMPPMEGINGAGGLQINGAMFGNPIAAPPQMNIYHQMGMGAAADQMDMGVAAGQMGIGAAASQMGMGTAGASGFDVAAPESRPSSMVSQKDEHANAAEISSMMSVTGPGSATTTIEMDGLWKYKY

Sspon.03G0022860-1A	*SsNAC045*	ATGTACCCCTCTCCCTCACTGCTC<br>GCTCTCTCCTTTGCACCCCTAGTA<br>CTACTGCTCCTC<br>CGACTTGCTCTTCGCGCAGCAAG<br>ACGGAGAGACAAGAATCATCCCT<br>CCCCTACTACTACT<br>ACTACTACTACTACTCATGCATGC<br>GCACTGCCTCCTGGTGCCGCGTAT<br>GAACCAGACATG<br>AATAGGGGGCACAGCAGCAGCTC<br>TAAGCTCATCAACGAGAAGCTCG<br>AGGAACACCGGATC<br>TCCACCGCGAAGCACTGCCCCCA<br>CTGCGGCGAGAAAATCGACAGCA<br>AACCGGATTGGGTG<br>GGGCTGCCGGCAGGCGTCAAGTT<br>CGATCCAACTGACCAGGAGCTGA<br>TCGAGCACCTCGAG<br>GCGAAAGTGAAGGATGAAGGCTC<br>GAGATCTCACCCTCTCATCGACGA<br>GTTCATACCCACG<br>ATAGATGGGGAGGACGGCATATG<br>TTACACCCACCCCGAGAAACTTCC<br>AGGTGTGACGAGG<br>GATGGCCTAAGCAAGCACTTCTTC<br>CACCGGCCGTCCAAGGCCTACAC<br>GACGGGCACGAGG	MYPSPSLLALSFAPLVLLLL<br>RLALRAARRRDKNHPSPTT<br>TTTTTTHACALPPGAAYEP<br>DM<br>NRGHSSSSKLINEKLEEHRIS<br>TAKHCPHCGEKIDSKPDWV<br>GLPAGVKFDPTDQELIEHLE<br>AKVKDEGSRSHPLIDEFIPTI<br>DGEDGICYTHPEKLPGVTR<br>DGLSKHFFHRPSKAYTTGT<br>R<br>KRRKIQTECDVHKGETRWH<br>KTGKTRPVMVNGRQKGCK<br>KILVLYTNFGKHRKPEKTN<br>WVMH<br>QYHLGDLEEEKEGELVVCK<br>IFYQTQPRQCSWSSDRGAA<br>ATAMVATTSAAQEQHRRD<br>SGSG<br>SCSSRDHEVSATSFPAGYTV<br>TTAVEMQQHMKQSADHFS<br>FAPFRKTFDQEVGIGGDQVP<br>SN<br>QLGRSEPHHAGQGQPHGPV<br>LATTTAMPATAFLISRPSNP<br>VSTIVPPAMQHASVVLDHD<br>QF

Sspon.03G0025270-1A	*SsNAC046*	ATGGAAGGCGCAGCAGCAGGAGG<br>AAGAGGAGGTTCGTCGTTGGTGT<br>TCCGCGGGTGCCAT<br>CTGCCGCCGGGCTTCCGATTCCAA<br>CCGACAGACCAAGAGATCATCGT<br>CTGCTACCTCAAG<br>AAGAAGATCGCCGGCACCGCCAC<br>CTCTGTCACCTCCATCATCGCCGA<br>CGTCGACATCTAC<br>AAGTTCGACCCATGGGACCTCCCT<br>GACAAGGCCATGTTCGGCGAGGG<br>CGAGTGGTTCTTC<br>TTCAGTCCTCGTGACCGCAAGTAC<br>CCCAACGGCGCCCGCCCCAACCG<br>CACGGCGGGCTCC<br>GGCTACTGGAAGGCCACCGGCAC<br>CGACAAGCCCATCCTGGCGGCCG<br>GCGGCGCGCACTGC<br>CTGGGCGTCAAGAAGGCGCTCGT<br>CTTCTACCAGGGGCGCTCCCCGA<br>AGGGCAGCAAGACG<br>GAGTGGGTCATGCACGAGTACCG<br>CCTCCTCGACACCGACGCCGCCGC<br>GGTGCTGGCCAGG<br>CCCGCCGCCGCCAACTCCATGAG<br>GCTTGACGACTGGGTCCTCTGCCG<br>CGTCCGCAAGAAA	MEGAAAGGRGGSSLVFRGC<br>HLPPGFRFQPTDQEIIVCYLK<br>KKIAGTATSVTSIIADVDIY<br>KFDPWDLPDKAMFGEGEW<br>FFFSPRDRKYPNGARPNRTA<br>GSGYWKATGTDKPILAAGG<br>AHC<br>LGVKKALVFYQGRSPKGSK<br>TEWVMHEYRLLDTDAAAV<br>LARPAAANSMRLDDWVLC<br>RVRKK<br>GVSLGPADMDDTSEGTTTT<br>ATTRAAVPANAGTDYHRA<br>ETMEMATAAAAYSRREVL<br>VPDAG<br>TGGFFGDVVIDCNKNDDDD<br>GGDDLLQYFIASGGLSGSPS<br>AHHMHHDQGHGHGGMVP<br>VPGA<br>ASAAPLHTQHHHGLVSVLE<br>SIKRNLSFQAIDELYLLQPSA<br>KRANYMTTMLSRGDDDDH<br>HQ<br>HSMSSPTCFSISDTDEVF

Sspon.03G0026820-1B	*SsNAC047*	ATGGAGGAGGAGGAGGCGAATCT<br>GAGCGCCGGCGACGGCGAGGAGG<br>AAGAGGAAGCGGCG<br>GAGCGCGGTTCCTGCGGCGGCGG<br>GTTCGACTTCGACCCGACGGAGG<br>ACGAGTTGGTGCTG<br>CACTTCCTGCGTCCGCAGCTGCGC<br>GGGTTCCCGCCGCGCGTGGCGGG<br>CGCCGTACTGGAG<br>GCGGACCCGTGCGGCGCGGCGCC<br>GTGGGAGCTGCTGGCGCGGTACG<br>GCCTGCGGGAGCGG<br>GGCCACTTCTTCGTGGCGCGCGG<br>GCGGGGGCGGGGGCGGACCGCCG<br>TGCGCCGCGCCGTG<br>GCCGGCGCCGGCTCCTGGATGCG<br>CAGCGCCACCAGGCTCGGGCAGT<br>CCGTGACGGACCTC<br>GGCGTCGTGGTGCGGTGGAGCAG<br>GGCCAAGTTCTGCTTCTACGTGCC<br>CCAGCCGGGTCCG<br>GGGCCGGGGCCGGGGCAGGGGCG<br>GCAGCTGCGGAGCGCCGGGTGGG<br>TGCTGGAGGAGTAC<br>CAGATCACGGACCCGCGGTGCTA<br>CCGCCGCGCCGACGAGGAGGAGG<br>ACCGGTACTGGGTG	MEEEEANLSAGDGEEEEEA<br>AERGSCGGGFDFDPTEDEL<br>VLHFLRPQLRGFPPRVAGA<br>VLE<br>ADPCGAAPWELLARYGLRE<br>RGHFFVARGRGRGRTAVRR<br>AVAGAGSWMRSATRLGQS<br>VTDL<br>GVVVRWSRAKFCFYVPQPG<br>PGPGPGQGRQLRSAGWVLE<br>EYQITDPRCYRRADEEEDR<br>YWV<br>LCRVRRSGDHGRRTESEAG<br>AGMPPPSWNRVME

Sspon.03G0031890-1B	*SsNAC048*	ATGGTCGCGACCCGCTCCAAGAC<br>AGCGCCAGGCCCACGCCCACGCT<br>CACGGCCGCGGCCC<br>GACTTGGCGGCCCACCCATCCGA<br>GGAAGAGCTCATCACGTCCTTCCT<br>CCGACCCCGCGTC<br>GTCTGTGTCGCTGGCGACAGGCC<br>GTGCGCGTCGTTCATCCATGACGC<br>GGACGTCTACTCC<br>GCCGGCCCCGGCGAGCTCACGGG<br>CGGGCACGCTCCGGCGGTCGCGA<br>GCAACGGCGACAGC<br>GCATGGTACTTCTTCTCCGCCGTG<br>AGGGCCAAGACCCGGGACGGGCA<br>GCGCAAGGCGCGC<br>ACGGTGGACACCGGGGAAGGGTG<br>CTGGCACTCGGAGGCCGGCGCGA<br>AGCCCGTGGTGGAG<br>GAGGGGCACGGTGGCCGGGTCCT<br>AGTAGGGCACCGCCAGGGATTCT<br>CGTTCGTGACGAAG<br>GTGGACGGGCGGCGCGTCCGGTC<br>GGGGTGGCTGATGGTGGAGCTCA<br>GCCTCGACGGCGCC<br>GACGCCGACGACGTGGTCCTCTG<br>CAAGATCTACTTCAGCCCGCGCG<br>CGCGCGCCTCGGCG	MVATRSKTAPGPRPRSRPRP<br>DLAAHPSEEELITSFLRPRV<br>VCVAGDRPCASFIHDADVY<br>S<br>AGPGELTGGHAPAVASNGD<br>SAWYFFSAVRAKTRDGQR<br>KARTVDTGEGCWHSEAGA<br>KPVVE<br>EGHGGRVLVGHRQGFSFVT<br>KVDGRRVRSGWLMVELSL<br>DGADADDVVLCKIYFSPRA<br>RASA<br>AAASASSGRKRKAAADDK<br>NPANSARRRRRGRPTEAEA<br>GTPAPNDAEEKDNIQSRRGL<br>ADD<br>NSTVTDDPDALWTDDSFFS<br>WWMRNKDWLMEEYSIVD<br>RPNEENQKTYGLDEYLRLL<br>KHHYI<br>DFGCGHSPVLNS

Sspon.03G0035360-1B	*SsNAC049*	ATGGCAGACCAGCAGCAGGAGAT<br>GAACATGGTCCGTGCCGGCGGCC<br>TCGACCTACCTCCA<br>GGGTTCCGCTTCCACCCAAGTGAT<br>GTTGAGATTGTCAGTGACTACCTG<br>ATGAACAAGGTG<br>CGCAACACGAACTTCACCTGCAT<br>CGCCATCGGAGAGGCCGACATAA<br>ACAAGACTGAGCCA<br>TGGGACCTTCGGGATAAAGCAAA<br>ATGGGGCGAGAAAGAGTGGTACT<br>TCTTCTACCAGAAG<br>GACCGCAAGTACCCGACGGGGTT<br>GAGGGCGAACCGAGCCACAGTGG<br>GTGGTTATTGGAAA<br>GCCACAGGCAAAGATAAGGAGGT<br>CTACAAAACCATAGAAGGGGTGG<br>TGGTGTTGGTCGGT<br>ATGAAGAAGACGCTCGTCTTCTA<br>CAAGGGTAGGGCTCCTAAGGGCG<br>ATAAAACCAACTGG<br>GATGAGTGGGTGGTCTGTCGCGT<br>GTTTGAAAAGACCATTGGGATCA<br>AGAAGATGACTACA<br>CCAGCATACCAGGTCGCCATGGC<br>CAGCGCTGAGATTGATCAAATTC<br>AGAACAACATCCCG	MADQQQEMNMVRAGGLD<br>LPPGFRFHPSDVEIVSDYLM<br>NKVRNTNFTCIAIGEADINK<br>TEPIAGAGGFDVAAPESRPS<br>SMVSQKDEHANAAEISSMM<br>SVTGPGSASTTIEMDGLWK<br>YKY<br>WDLRDKAKWGEKEWYFFY<br>QKDRKYPTGLRANRATVG<br>GYWKATGKDKEVYKTIEG<br>VVVLVG<br>MKKTLVFYKGRAPKGDKT<br>NWDEWVVCRVFEKTIGIKK<br>MTTPAYQVAMASAEIDQIQ<br>NNIP<br>AIPDPMPLQLPLPVPMPMQF<br>PILPDFSMDPVAPYYPNAGA<br>GMPTMMPPMEGINGADEL<br>QI<br>NDAMFGNPIAAPPQMNIYH<br>QMGMGAAADQMDMGVAA<br>GQMGIGAAAGQMGIGAAA<br>SQMGMG

Sspon.03G0040250-1P	*SsNAC050*	ATGGACGGGTTCAACGGGGACGA<br>CAACGACGGCCACCACTTCAACG<br>ACGACGGCTACGCC<br>TCCGGCTTGGCCGGTGGATTCAAT<br>TCCGGCTACACCGGCGCCTTCAAC<br>TCCGGCTACGGC<br>GACGGCAGCTTCAACTCCGCGTA<br>CGGCGACGGCGGCTTCAACTCCG<br>GTTTCGGCGAAGGC<br>GGCCACACCCGGGACTTCCTCTCC<br>CAGCCGGTGTCCGTCTCCGCCTCC<br>GGTGCGCACACC<br>TCCGCCGGCGCGCCGCCTCCTGCC<br>TTCGCGTCGTCGAGCAACCCCAG<br>CCCAAGCCAACTG<br>AGGCTGGATTCGCTCGACCTCAA<br>CGCCAGCCAATCTTGGCCGGACA<br>TGGAAGCCTACCAA<br>GGAACGTCAACAAACAGAGTACT<br>ACACATCAACGAGGACACGGACG<br>AGGTCTGTTTGCGC<br>TGCACACGAACCAACCTAGCCTA<br>CGCGGCTACGTCCCGAGCGCCGG<br>GCCCCGCAGAAAGA<br>ACGCCTCGCATGCGCCTGCTGAA<br>ACCGAAGGAAGCCGCCGCACTCC<br>CGCGCGAGGCCCTC	MDGFNGDDNDGHHFNDDG<br>YASGLAGGFNSGYTGAFNS<br>GYGDGSFNSAYGDGGFNSG<br>FGEG<br>GHTRDFLSQPVSVSASGAH<br>TSAGAPPPAFASSSNPSPSQL<br>RLDSLDLNASQSWPDMEAY<br>Q<br>GTSTNRVLHINEDTDEVCLR<br>CTRTNLAYAATSRAPGPAE<br>RTPRMRLLKPKEAAALPRE<br>AL<br>EAHIVSLLRGCRGLLALRSA<br>HAHLTRLRLPRLAAAFALS<br>KLLASCASAPAPQAAAASS<br>YA<br>RSLFDQIPDPTAFCYNSLIRA<br>LPAPAPALAVYRRMLRAGS<br>PHPNTFTLAFALKACAAVP<br>A<br>TGEGRQLHAQALRQGLEAS<br>AYVQTGLLNLYARCEQVAL<br>AMTVFDGMAPDKSLIAWS<br>AMIS<br>GYSRVGMVNEALGLFREM<br>QAAGVEPDEVTMVGVISAC<br>AKAGALDLGKWVHAYIDR

Sspon.03G0040660-1C	*SsNAC051*	ATGGCCACGGTGCAGGGTCTTCCTCCTGGCTACCGTTTCGTGCCCACTGACGAGGAGTTGGTCGAGCTTTGCCTGCTCCCTCGCATCCAGGACCAGCCCCTCCTGCCGAACGACATCATCGAGGACGACCCGCTGAGCGCGCCGCCGTGGGCTCTCCTCGAGAAGCACGGGCGGAGGCACCAGGCCTTCTTCTTCGCGGCGTGTCAGGCCATGAACGCCAAGGGCAACCGCCAGAAGCGCTCCTGCGTGGGACACGGGACCTGGCAAGGGCAGGGCAAGAGGAAGCGCCAACAGGAAGAAGAAGGGCAGGAGGTCAAGAAGAAGCTGCGCGTGCCCGTGCGCGGTAGCGCCGAGAAGATTGAGATCGAGTGGGACAAGTACGCGCTCAACTTCCAGGAGCACGGTGTCAAGGGCAGCACGGGCTGGGTCATGCACGAGTACTCCATCACCGCCCCGCCCGAGCTCGCGCGGTCGCCGGTGAGGGTGTACTGCATCCGCTTCAGCGGCCACGGCAAGAACGCCAAGAAGAACAGCAGGGAT	MATVQGLPPGYRFVPTDEELVELCLLPRIQDQPLLPNDIIEDDPLSAPPWALLEKHGRRHQAFFFAACQAMNAKGNRQKRSCVGHGTWQGQGKRKRQQEEEGQEVKKKLRVPVRGSAEKIEIEWDKYALNFQEHGVKGSTGWVMHEYSITAPPELARSPVRVYCIRFSGHGKNAKKNSRDAQHRGDDDEFEDEEEVDDDAAGAIATTTRSAAEEDAALLIEDYLTSFPVVAVDVVSANPADGADAGAGYDQDLPGGGTFSRVDLPQPLGPRSIQSCPGGTRREHPLSTGNTLPRRVVTESPTSRPSMAGAAGGGKVEAHIAKLRRGGASRV

Sspon.04G0000480-1A	*SsNAC052*	ATGAGCCTATCACCGCTCGACTCC<br>TCCTCCTCCCCGCCGGCGGCGGCG<br>GAGGTGCCGCTC<br>GCCCCGGGCTTCCGCTTCCACCCC<br>ACCGACGAGGAGCTCGTCTCCTA<br>CTACCTCCGCCGC<br>CGCGTCCTCGGCCGCCGCCTCCGC<br>GTCGACGCCATCGCCGAGGTCGA<br>TCTCTACCGCCTC<br>GAGCCCTGGGACCTGCCCTCCCTC<br>TCCCGCATCCGCAGCCGCGACGC<br>CCAGTGGTACTTC<br>TTCGCCCGCCTCGACCGCAAGGTC<br>GCCGGCGCCGGCGCCGGGGGCCG<br>GGGCGGCCCCGGC<br>AACAGGACCAACCGCGCCACGCC<br>GAGGGGGTACTGGAAGACCACGG<br>GCAAGGACCGCGAG<br>GTGCACCACCGCGGCAAGCCCGT<br>CGGGATGAAGAAGACGCTCGTCT<br>TCCACGCCGGGAGG<br>GCGCCTAAGGGCGGGAGGACCAA<br>CTGGGTCATGCACGAGTACCGCC<br>TCCTCGACGCAGAC<br>GGGCCTCAGGATCTGCATGTGGT<br>GTGTAGAATCTTCCAGAAGCATG<br>GATCTGGACCACAG	MSLSPLDSSSSPPAAAEVPL<br>APGFRFHPTDEELVSYYLRR<br>RVLGRRLRVDAIAEVDLYR<br>L<br>EPWDLPSLSRIRSRDAQWYF<br>FARLDRKVAGAGAGGRGG<br>PGNRTNRATPRGYWKTTG<br>KDRE<br>VHHRGKPVGMKKTLVFHA<br>GRAPKGGRTNWVMHEYRL<br>LDADGPQDLHVVCRIFQKH<br>GSGPQ<br>NGAQYGAPYMEEEWEEDD<br>DAIENGPTSGASAQMAAITC<br>AVDEESNEDDENAYCETNR<br>PAR<br>VEPSHLPLVHEMLNPPEMA<br>PLQGQDSKETSDGSCADGAI<br>SLEEILQEPLSNISVENIGRL<br>EGQNATDDSINVDDLLSAY<br>PRKDNGYVGQDNTMNGSG<br>PADGDHTSWPLRAYSNQNY<br>VNGP<br>LADEFFDTGNDTNGVAYSG<br>HQQADGFPAPRQVDDSMVF<br>YDAPSDYNLVDGNDDFVYL<br>NDL

Sspon.04G0003490-1A	*SsNAC053*	ATGGATGAGATCAGAAGTGATGA CATAGAGAAGCAAGATGAAGTTA TGCTACCTGGATTC AGGTTTCATCCAACAGATGAAGA GCTTGTCAGGTTTTACCTTAAAAG AAAAATCCAGAAG AAGTCTCTCCCCATTGAGCTCATC AGGCAGCTAGACATCTACAAGTA TGATCCATGGGAT CTCCCAAAACTAGCGAGTACTGG AGAGAAGGAATGGTATTTCTACT GTCCAAGGGATAGG AAGTACCGGAACAGCACAAGACC AAACAGGGTAACTGGAGCAGGCT TCTGGAAGGCCACT GGAACTGACAGGCCAATCTACTC TTCTGATGGGAGCAAGTGCATAG GCTTGAAGAAATCT CTTGTCTTCTACAAAGGTAGAGCA GCCAAAGGTGTCAAAACCGACTG GATGATGCATGAA TTCAGGTTGCCATCACTCACTGAC CCATCACTGCCGCAGAAGAAGCC ACTGGAGAAGACC ATTCCACCAAATGATTCCTGGGCG ATCTGCAGGATTTTCAAGAAAAC CAATTCAACAGCA	MDEIRSDDIEKQDEVMLPGF RFHPTDEELVRFYLKRKIQK KSLPIELIRQLDIYKYDPWD LPKLASTGEKEWYFYCPRD RKYRNSTRPNRVTGAGFW KATGTDRPIYSSDGSKCIGL KKS LVFYKGRAAKGVKTDWM MHEFRLPSLTDPSLPQKKPL EKTIPPNDSWAICRIFKKTNS TA QRALSHSWVSPPLSSTNEN YIRPSSQATQRSHHSSENTSS TMTDIISSIQFTGSSYMPSI VSSCRNPASIIDSSSRLAASL VLPSAGAEHHTMSVLSAIPL DLPAGVDIASMVLNASPTT LQNLDRIPPNIEFGQPHHPSN SNSMANRCTVDLPDIGNSV TAAPRSINFPFNLQGALLDD WRMTLPWDSLPCTTEVSTN YQSTKCYT

Sspon.04G0003500-4D	*SsNAC054*	ATGGATGGAACCCATCAACAGGT<br>GGTCTCGGAGGTGGTGTCCACGTT<br>CTTGCTGGTGTTC<br>GTGACGTGCGGGGCGGCGGGGAT<br>CTACGGCAGCGACAAGGACCGCA<br>TATCGCAGCTGGGG<br>CAGTCGGTCGCCGGCGGGCTCAT<br>CGTCACGGTGATGATCTACGCCGT<br>CGGCCACATCTCC<br>GGCGCGCACATGAACCCCGCCGT<br>CACGCTCGCGTTCGCCGTCTTCCG<br>CCATTTCCCCTGG<br>ATTCAGGTCCCGTTCTACTGGGCG<br>GCGCAGTTCACCGGCGCCATCTG<br>CGCGTCGTTCGTG<br>CTCAAGGCCGTGCTGCACCCCATC<br>TCCGTGCTGGGCTGCACCACGCCC<br>ACGGGGCCGCAC<br>TGGCACTCGCTCATCATCGAGATC<br>ATCGTCACCTTCAACATGATGTTC<br>GTCACCCTCGCC<br>GTCGCCACGGACACGAGAGCGGT<br>GGGTGAGTTGGCGGGGTTGGCAG<br>TTGGTTCCTCGGTT<br>TGCATTACGTCCATCTTCGCAGGG<br>GCAGTGTCGGGCGGATCGATGAA<br>CCCGGCGAGGACG	MDGTHQQVVSEVVSTFLLV<br>FVTCGAAGIYGSDKDRISQL<br>GQSVAGGLIVTVMIYAVGH<br>IS<br>GAHMNPAVTLAFAVFRHFP<br>WIQVPFYWAAQFTGAICAS<br>FVLKAVLHPISVLGCTTPTG<br>PH<br>WHSLIIEIIVTFNMMFVTLA<br>VATDTRAVGELAGLAVGSS<br>VCITSIFAGAVSGGSMNPAR<br>T<br>LGPALASNLYTGLWIYFLGP<br>VLGTLSGAWTYTFIRFDEPP<br>SGHKDMSHSQKLSSFKLRR<br>L<br>QSQSVAADDEELDHIQLEV<br>GDHCLRYTASSQWFVLLCK<br>HMDEIRSDDIEKQDEVMLP<br>GFR<br>FHPTDEELVRFYLKRKIQKK<br>SLPIELIRQLDIYKYDPWDLP<br>KLASTGEKEWYFYCPRDRK<br>YRNSTRPNRVTGAGFWKAT<br>GTDRPIYSSDGSKCIGLKKS<br>LVFYKGRAAKGVKTDWM<br>MHEF

Sspon.04G0004240-3D	*SsNAC055*	ATGAGCAGCTCCAAGCACGCGGC<br>GGCCGGCGCCGCCGCCGTGGGCG<br>GCAAGGCCGCGCGC<br>GCCTGCGACAGCTGCCTGCGGCG<br>GCGCGCGCGGTGGTACTGCGCGG<br>CCGACGACGCCTTC<br>CTGTGCCAGGGCTGCGACGCGTC<br>GGTGCACTCGGCGAACCCGCTCG<br>CCAGGCGGCACGAG<br>CGCCTACGCCTGCGCCCGACGAC<br>GTCGCGGCCGGACCCGCCGCACT<br>CGACGCTCGAGGCT<br>GGCGTCGGCGTGGCGGCGTCCAC<br>GTCGACGTGGAAGAAGCGCCAGC<br>AGCAGCAGGTCGCG<br>CCGGCGGCGTGGTCCAAGCGCAA<br>GGCCCGCACGCGGAGGCCGCACG<br>TCAAGAGCGTCGGG<br>CAGCTGCTGTCGAGGAAGCTCGT<br>CGTCGTGCCTGAGGTGGCCAGCG<br>TCGAGTCGTCGGAG<br>GAGCGGAAGGCCGAGGAAGAGG<br>AAGAGGAGGAGGAGGAGCAGCT<br>GCTGTACTGCGTGCCG<br>ACCTTTGACCGCGCCCTGGCCGA<br>GCTCTGCACGCCGCCGCCGCCTCC<br>GGTGGATGACCCG	MSSSKHAAAGAAAVGGKA<br>ARACDSCLRRRARWYCAA<br>DDAFLCQGCDASVHSANPL<br>ARRHE<br>RLRLRPTTSRPDPPHSTLEA<br>GVGVAASTSTWKKRQQQQ<br>VAPAAWSKRKARTRRPHV<br>KSVG<br>QLLSRKLVVVPEVASVESSE<br>ERKAEEEEEEEEEQLLYCVP<br>TFDRALAELCTPPPPPVDDP<br>TATASSSCCRDDVDVAVEN<br>TKAPASPAVVVAESPVQQL<br>PDSFAGFGPTDAELREFAAD<br>ME<br>ALLGQGLGDSNELDESFYM<br>ESLGLMTTTQQAEDVGRVK<br>MEPNGSVISRSRSESAPGFC<br>PA<br>ELMKPEASSAEVLDIDFNCS<br>SPTVMMDNEDEDSFEQKAS<br>ASNGGDAAAGTQFLKRSLD<br>LS<br>LNYEAIIESWGSSPWTDGQR<br>PNVQLDDFWPHAHLTVRDL<br>RIHTPLFLVLDSIDRARIYIC<br>FNLQLAIASTIGFMQGLLRG

Sspon.04G0007420-1A	*SsNAC056*	ATGGACACTTTCTCTCATGTCCCC<br>CCTGGATTCCGTTTCCACCCCACC<br>GACGAGGAACTC<br>GTCGACTACTACCTCAGGAAGAA<br>GGTGTCGTCCAACAAGATCGACC<br>TTGACGTCATAAAA<br>GACGTCGATCTGTACAAAATTGA<br>GCCTTGGGATCTCCAAGAGAAGT<br>GCAAGATTGGGATG<br>GAGGAGCAGAACGAGTGGTACTT<br>CTTCAGCCACAAGGACAAGAAGT<br>ACCCGACGGGCTCT<br>CGCACCAACCGGGCGACCACCGC<br>CGGCTTCTGGAAGGCGACGGGGC<br>GCGACAAGCCCATC<br>TACACCAAGAGCTGCCTCGTCGG<br>GATGAGGAAGACGCTCGTCTTCT<br>ACAGGGGCCGCGCG<br>CCCAACGGCCAGAAGTCCGACTG<br>GATCATGCACGAGTACCGCCTGG<br>AGACCACCGAGAAC<br>GGGACCGCCCCTGAGGAAGGATG<br>GGTGGTCTGCAGGGTGTTCAAGA<br>AGCGAGTGGCGACC<br>GTGCGGAGGATGGCCGACGGCGC<br>GCCGTGGTTCGATGACCATGTCGC<br>CGGCGGGTTCATG	MDTFSHVPPGFRFHPTDEEL<br>VDYYLRKKVSSNKIDLDVI<br>KDVDLYKIEPWDLQEKCKI<br>GM<br>EEQNEWYFFSHKDKKYPTG<br>SRTNRATTAGFWKATGRD<br>KPIYTKSCLVGMRKTLVFY<br>RGRA<br>PNGQKSDWIMHEYRLETTE<br>NGTAPEEGWVVCRVFKKR<br>VATVRRMADGAPWFDDHV<br>AGGFM<br>PDLIGSPNQLMHHPNAAAA<br>VYSGGQQQLYHCKPELEYH<br>HLLPSQDAFLQQLPQLESPK<br>PP<br>PAYIAQGSCSLQSPDEASGY<br>AAQQPPLTEAAYMDDSVTD<br>WRVLDKFVASQLFSHGDGT<br>AK<br>EAGYPNPAPAFQAENKQPE<br>GALGYASTSASGGGEAS

Sspon.04G0009100-1A	*SsNAC057*	TCATGGCTGATAACTGGTAGGGG<br>AATTGCTAAGAAAATAAGATACG<br>CAGCTCCTAGCGCC<br>AATCACCAAATAAGTGAACTGAT<br>TGCAGAAGCACGGAGGGAATGCC<br>CAAACTGCAGTTAT<br>GTTATTGATAACAGCGATGTTGCC<br>ATGCAGTGGCCTGGGCTGCCTGCT<br>GGAGTCAAGTTT<br>GATCCTTCTGAATTGGAGTTGCTT<br>GAACATTTAGAACAAAAAGTTGG<br>CCTGGGAGGTTCA<br>AGACCACATGTGCTCATTGATGA<br>ATTTATTCCAACTATAGACAATGA<br>TGAGGGAATCTGC<br>TATTCACATCCTGAAAATCTTCCT<br>GGTATGAAAACAGATGGTAGCAA<br>TGCTCATTTCTTC<br>CATAGAGCTTCAAACGCGTATGG<br>TTGTGGACAGCGCAAGCGTCGAA<br>GGATCATCAACTGC<br>AGCGATCACACTGTTCCTGATGA<br>GCATGTGAGATGGCACAAGACAG<br>GGAGATCCAAACCC<br>ATATATGACAATGGTGTTATAAA<br>AGGTTGGAAGAAGATACTGGTGC<br>TGTACAAAACTTCG	SWLITGRGIAKKIRYAAPSA<br>NHQISELIAEARRECPNCSY<br>VIDNSDVAMQWPGLPAGV<br>KF<br>DPSELELLEHLEQKVGLGGS<br>RPHVLIDEFIPTIDNDEGICY<br>SHPENLPGMKTDGSNAHFF<br>HRASNAYGCGQRKRRRIIN<br>CSDHTVPDEHVRWHKTGRS<br>KPIYDNGVIKGWKKILVLY<br>KTS<br>QRGGKPDRANWYHLGEEE<br>NEKDGELVVSKIFCQLPNKS<br>MEISETETAYEEPDAPASVI<br>GP<br>KTPKTITPKPRHPKNSPCET<br>EQNIPILQD

Sspon.04G0010300-1A	*SsNAC058*	ATGCGCCGCCTTCTCCCGCCGCTC GCCGTCAACCCGCCGCCATGCCTC CCAGCACCTCCT CCAACCGCCGCCGGCGGAAGCGC CCTCGCCTTCCCGGAGTTGATTCC GCTCGTGGAGCGG GCCATCTCAACCGGGGACGTGCT CCGTCTCGGCCGCGCAGTGCACG CGCACCTCGTCAAG ACGGCGCTCACCAGCCATACGCT CCTGTCCAATCGCCTCGTCGCGCT CTACTCCCGGCTC CCGTCGCCGGCCGCTGCGGTCTCG GCTTTCGAAGACCTTCCACACAA GAACGCGCACTCC TACAACACGCTTCTCGCCGCGCTC TTGTGCAGGCCGGACACCTTCCCC GACGCCCTCCAC CTGTTCGACACAATGCCGGCCGA CTCACGCAACCTCGTCTCGTACAA CACCCTCATGTCG TCCCTCGCGCACCACGGCAGGCA AGTGGAGGCGCTCCGCGTGGTTG CCCGGCTGGCCAGG GACAGGTTCTTGGGGCCAGGGTT AGCCATGGATCGGTTCACAGTGG TCAGCGTGGCAACT	MRRLLPPLAVNPPPCLPAPP PTAAGGSALAFPELIPLVER AISTGDVLRLGRAVHAHLV K TALTSHTLLSNRLVALYSRL PSPAAAVSAFEDLPHKNAH SYNTLLAALLCRPDTFPDAL H LFDTMPADSRNLVSYNTLM SSLAHHGRQVEALRVVARL ARDRFLGPGLAMDRFTVVS VAT ACAGIRAERPLREMHCAVV VSGMELTVIMANAMVNAY SKGGRVEDARHLFNQVSIR DNVT WTSMISGYCQVKKLDEAV QVFDMMPDKDRVAWTALI SGHEQNGEEDTALELFEGM LAEGV SPTPFALVSALGACAKLGL VTRGKELHCFILRRSIGFDPF NIFIHNALVDMYSKCGDMT A TMAVFRRMPEKDFISWNSM VTGFSHNGLGKQSLAIFEEM LVAGVRPTHVTFLAVLTAC

Sspon.04G0014100-1A	*SsNAC059*	ATGTATCCGAGCTGCTCTCCACTG<br>GCAGTGCCTCCGGGCTTCCGGTTC<br>CACCCGACCGAC<br>GAGGAGCTACTCTACTACTACCTG<br>AGGAAGAAGGTTGCTTATGAGCC<br>CATAGATCTTGAT<br>GTCATAAGGGAGGTCGACCTCAA<br>CAAGCTTGAACCATGGGACCTCA<br>AAGTGGTATTTCTT<br>CAGCCACAAGGACAAGAAGTATC<br>CTACGGGGACACGAACCAACCGG<br>GCAACAACGGCTGG<br>GCCATCTTCCTCGGCAACGGTGG<br>GAGGAGGATCGGCCTGAGGAAGA<br>CGCTGGTGTTTTAC<br>ACCGGCAGGGCACCCCATGGCAA<br>GAAGACGGACTGGATCATGCACG<br>AGTATCGCCTCAAC<br>GATGACAACGTCGAGGAGGATGG<br>ATGGGTGGTGTGTAGGGTTTTTAA<br>GAAAAAGAGCATC<br>CAGAGGGGATTATTCGACGACCA<br>GCTGGGCATGGCGCCGGCGGCTG<br>GAGATGACGACGAA<br>CTCCATTCCTTCCACTCCCCGGGC<br>GGCATGACTCCGGTGGAGCAGAA<br>GTACGGCCTCCAC	MYPSCSPLAVPPGFRFHPTD<br>EELLYYYLRKKVAYEPIDL<br>DVIREVDLNKLEPWDLKVV<br>FL<br>QPQGQEVSYGDTNQPGNNG<br>WAIFLGNGGRRIGLRKTLVF<br>YTGRAPHGKKTDWIMHEY<br>RLN<br>DDNVEEDGWVVCRVFKKK<br>SIQRGLFDDQLGMAPAAGD<br>DDELHSFHSPGGMTPVEQK<br>YGLH<br>LHQLMHGGVPAFDPSMHLP<br>HLTTAEGTLGAPSFTSGTSA<br>VAMNLLDMGCSPHNMVK<br>MTKS<br>CGSASDMPLNSGERFGAAA<br>DWSILDKLLASHQNLDQLF<br>HCKFGGTTLAAPHHYQQQQ<br>MQQ<br>QHIEMSASSLHRLPLH

Sspon.04G0022660-1B	*SsNAC060*	ATGATTGGGAGACGGTTTAATTC<br>ATTTTTACCGTACACAGAGGGCA<br>GCATAGCTGGAGGA<br>ACTGCTGGTGTTGTGGTGGAAAC<br>GGCACTGTACCCGATTGATACTAT<br>AAAAACCAGGCTT<br>CAGGCTGCTCGAGGTGGAAGCCG<br>GATTGAATGGAAAGGCTTGTATT<br>CTGGATTAGCTGGA<br>AATCTTGCTGGTGTCCTTCCGGCC<br>TCTGCAATATTTGTCGGAGTGTAT<br>GAGCCAACTAAA<br>AGAAAATTACTGGAGACACTTCC<br>TGAAAATCTGAGTGCAGTTGCCC<br>ATTTTACTGCAGGG<br>GCCATTGGAGGCATTGCAGCTTCC<br>CTTGTCCGTGTCCCTACAGAGGGT<br>TATGGTTCCTTT<br>CTACTTCGAGATCTTCCATTCGAT<br>GCCATTCAATTCTGCATTTATGAG<br>CAACTTCGAATT<br>GGCTACAAAGTTGTGGCAAGAAG<br>GGAGTTGAATGATCCAGAGAATG<br>CACTTATCGGTGCT<br>TTTGCTGGCGCCATCACTGGAGCT<br>ATAACAACTCCCCTTGATGTCATG<br>AAGACAAGATTG	MIGRRFNSFLPYTEGSIAGG<br>TAGVVVETALYPIDTIKTRL<br>QAARGGSRIEWKGLYSGLA<br>G<br>NLAGVLPASAIFVGVYEPTK<br>RKLLETLPENLSAVAHFTA<br>GAIGGIAASLVRVPTEGYGS<br>F<br>LLRDLPFDAIQFCIYEQLRIG<br>YKVVARRELNDPENALIGA<br>FAGAITGAITTPLDVMKTRL<br>MVQGQGNQYTGIVSCAQTI<br>LREEGPKAFLKWDAVENKS<br>TVLTYAGGAVVALWLTFVI<br>VGA<br>INSVPLLPKIMELVGLGYTG<br>WFVYRYLLFKESRKELADD<br>IESLKKKIAGTETYPIRNPCD<br>SDFLNRPGLPAKLGKLDVN<br>DSTRVAYHVLDGMLMSSW<br>LIDCKRMASKIKNASGPTHH<br>ISN<br>TWKSNPTKECPKCNHIIDNS<br>DFVDEWPGLPKGVKFDPSD<br>QELLWHLLAKIGKVGLKPH<br>AF<br>IDEFIPTIDSNEGLYYTYPQK

Sspon.04G0024580-1B	*SsNAC061*	ATGTCGGCGGCGGCGCAGATCCC<br>ATCCCCACCCACCTACATCCCATC<br>CATGTCGTCGTCG<br>TCGGCGGCGGCGGAGCAGATCCC<br>ATCCCCACCCACCTACAGGTTCCG<br>GCCTAAGAAGCGC<br>GAGCTCGTCGAGTTCTACCTCCTC<br>CCGCGCGCCCGTGGCCAGGACCC<br>ATTCCCCGGCGTC<br>ATCTTCGAGGACGACGCGGCGGG<br>GAGCTCGCTGCCCTGGGATCTCTT<br>CGAGCGCCACGGC<br>GTGGGGAGCGAGGACGAGGCCTA<br>CTTCATCGTGCGCTCCAGTGAAGC<br>CAAGAAACCCGGC<br>GCCCGCCAGGACCGCGCCTGCGA<br>TGGCGGCGTCGGGACGTGGAAGA<br>CGCAGAGCAGCGTC<br>GAGAAGTCCCTGCGCGTCCGCGG<br>CGAGAGGATCAGGGTCCATGTCA<br>GCAACCTCAACCTG<br>CACATGGGCAAAGGCAAGAACAG<br>CGGCAGCGTGGGGTGGGTGATGC<br>ACGAGTACACCATC<br>GCCGCGCCGCCCTGCTCGTCGCTC<br>GTCAAGATCTGCCACATCACCTTC<br>AGCGGCCACGGC	MSAAAQIPSPPTYIPSMSSSS<br>AAAEQIPSPPTYRFRPKKRE<br>LVEFYLLPRARGQDPFPGV<br>IFEDDAAGSSLPWDLFERHG<br>VGSEDEAYFIVRSSEAKKPG<br>ARQDRACDGGVGTWKTQS<br>SV<br>EKSLRVRGERIRVHVSNLNL<br>HMGKGKNSGSVGWVMHE<br>YTIAAPPCSSLVKICHITFSG<br>HG<br>RMRMRVLDGQQVRQSSDH<br>ARVGATVVSGSSALPCSGA<br>MLDHGSSGVAYASSALPCS<br>GAML<br>NHGSSGVAYASGDEEPSHL<br>VLIDDDNFRQSGFSSAASEQ<br>YTELEVQVPTTKQQDIVVP<br>QL<br>MGQQSTMPVAQQLSAGEL<br>EFWSSIGVDVQSNNSFDQE<br>QSTGVQSSWVVPNNSAMA<br>TGAQS<br>SWVAPNTGAMATGVHSSW<br>VVPNNGAMATGAQSSRVV<br>PNTGAMATGVQSSWVAVD<br>LDDFCR

Sspon.04G0031350-2D	*SsNAC062*	ATGGATGAGATCAGAAGTGATGA<br>CATAGAGAAGCAAGATGAAGTTA<br>TGCTACCTGGATTC<br>AGGTTTCATCCAACAGATGAAGA<br>GCTTGTCAGGTTTTACCTTAAAAG<br>AAAAATCCAGAAG<br>AAGTCTCTCCCCATTGAGCTCATC<br>AGGCAGCTAGACATCTACAAGTA<br>TGATCCATGGGAT<br>CTCCCAAAACTAGCGAGTACTGG<br>AGAGAAGGAATGGTATTTCTACT<br>GTCCAAGGGATAGG<br>AAGTACCGGAACAGCACAAGACC<br>AAACAGGGTAACTGGAGCAGGCT<br>TCTGGAAGGCCACT<br>GGAACTGACAGGCCAATCTACTC<br>TTCTGATGGGAGCAAGTGCATAG<br>GCTTGAAGAAATCT<br>CTTGTCTTCTACAAAGGTAGAGCA<br>GCCAAAGGTGTCAAAACCGACTG<br>GATGATGCATGAA<br>TTCAGGTTGCCATCACTCACTGAC<br>CCATCACTGCCGCAGAAGAAGCC<br>ACTGGAGAAGACC<br>ATTCCACCAAATGATTCCTGGGCG<br>ATCTGCAGGATTTTCAAGAAAAC<br>CAATTCAACAGCA	MDEIRSDDIEKQDEVMLPGF<br>RFHPTDEELVRFYLKRKIQK<br>KSLPIELIRQLDIYKYDPWD<br>LPKLASTGEKEWYFYCPRD<br>RKYRNSTRPNRVTGAGFW<br>KATGTDRPIYSSDGSKCIGL<br>KKS<br>LVFYKGRAAKGVKTDWM<br>MHEFRLPSLTDPSLPQKKPL<br>EKTIPPNDSWAICRIFKKTNS<br>TA<br>QRALSHSWVSPPLSSTNEN<br>YIRPFSQATQRSHHSSENTSS<br>TMTDIISSIQFTGSSYMPSI<br>VSSCRNPASIIDSSSRLAASL<br>VLPSAGAEHHTMSVLSAIPL<br>DLPAGMDIASMVLNASPTT<br>LQNLDRIPPNIEFGQPHHPSN<br>SNSMANRCTVDLPDIGNSV<br>TAAPRSINFPFNLQGALPDD<br>WRMTLPWDSLPCTTEVSTN<br>YQSTNIARHFLVPALLNSHD<br>ALAMAGARASARRRAVLA<br>AVI<br>TLFLLASVSFLLSATATSSAS<br>AAANSPASRLAIVQRHAED<br>HAAVLAAYTAHARHLSAAS

Sspon.04G0035420-1D	*SsNAC063*	ATGTCGTCGGCGGCGGCGGAGCA<br>GATCCCATCCCCACCCACCTACAG<br>GTTCCGGCCTAAG<br>AAGCGCGAGCTCGTCGAGTTCTA<br>CCTCCTCCCGCGCGCCCGTGGCCA<br>GGACCCATTCCCC<br>GGCGTCATATTCGAGGACGACGC<br>GGCGGGGAGCACGCTGCCCTGGG<br>ATCTCTTCGAACGC<br>CACGGCGTGGGGAGCGAGGATGA<br>GGCCTACTTCATCGTGCGCACCAA<br>CGAAGCCAAGAAA<br>CCCGGCGCCCGCCAGGATCGCGC<br>CTGCGATGGCGGCGTCGGGACAT<br>GGAAGAAACAGAGC<br>AGCGTCGAGAAGTCCCTGCGCGT<br>CCGCGGCGAGAGGATCAGGGTCC<br>ACGTCAGCAACCTC<br>AACCTGCACATGGGCAAGGGCAA<br>GAACAGCGGCAGCGTGGGGTGGG<br>TGATGCACGAGTAC<br>AACATCGCCGTGCCGCCCTGCCC<br>GTCGCTCGTCAAGATCTGCCACAT<br>CGCCTTCAGCGGC<br>CACGGTCGGATGCGCATGCGTGT<br>GCCAGATGGCCAACAAGTCCGCC<br>AGAGTAGTGGTCAT	MSSAAAEQIPSPPTYRFRPK<br>KRELVEFYLLPRARGQDPFP<br>GVIFEDDAAGSTLPWDLFER<br>HGVGSEDEAYFIVRTNEAK<br>KPGARQDRACDGGVGTWK<br>KQSSVEKSLRVRGERIRVH<br>VSNL<br>NLHMGKGKNSGSVGWVM<br>HEYNIAVPPCPSLVKICHIAF<br>SGHGRMRMRVPDGQQVRQ<br>SSGH<br>ARCATAASGSSTLPCSGAM<br>LDHGSSGVAYASGDEEPSH<br>LVLIDDDNFRQSPLLGSSDF<br>QG<br>FPFAASEQYTELEAQVRTTE<br>QQDIAVPQLMVQQSTMQV<br>AQQLSAGELEFWSSIGVDV<br>QSN<br>NGFDLEQSTGVQSSWVVPN<br>NNAMATGAQSGWVAPNTG<br>AMATGVQSSWVVPNNGAM<br>ATGAQ<br>SSWVVPNTGAMATGVQSS<br>WVVPNTGAMATGVQSSWV<br>AGDLDDFCRNHSEPRSMEQ<br>QFLVS

Sspon.04G0035430-1D	*SsNAC064*	ATGTCGTCGGCGGCGGCGGAGCA<br>GATCCCATCCCCACCCACCTACAG<br>GTTCCGGCCTAAG<br>AAGCGCGAGCTCGTCGAGTTCTA<br>CCTCCTCCCGCGCGCCCGTGGCCA<br>GGACCCATTCCCC<br>GGCGTCATATTCGAGGACGACGC<br>GGCGGGGAGCACGCTGCCCTGGG<br>ATCTCTTCGAACGC<br>CACGGCGTGGGGAGCGAGGATGA<br>GGCCTACTTCATCGTGCGCACCAA<br>CGAAGCCAAGAAA<br>CCCGGCGCCCGCCAGGATCGCGC<br>CTGCGATGGCGGCGTCGGGACAT<br>GGAAGAAACAGAGC<br>AGCGTCGAGAAGTCCCTGCGCGT<br>CCGCGGCGAGAGGATCAGGGTCC<br>ACGTCAGCAACCTC<br>AACCTGCACATGGGCAAGGGCAA<br>GAACAGCGGCAGCGTGGGGTGGG<br>TGATGCACGAGTAC<br>AACATCGCCGTGCCGCCCTGCCC<br>GTCGCTCGTCAAGATCTGCCACAT<br>CGCCTTCAGCGGC<br>CACGGTCGGATGCGCATGCGTGT<br>GCCAGATGGCCAACAAGTCCGCC<br>AGAGTAGTGGTCAT	MSSAAAEQIPSPPTYRFRPK<br>KRELVEFYLLPRARGQDPFP<br>GVIFEDDAAGSTLPWDLFER<br>HGVGSEDEAYFIVRTNEAK<br>KPGARQDRACDGGVGTWK<br>KQSSVEKSLRVRGERIRVH<br>VSNL<br>NLHMGKGKNSGSVGWVM<br>HEYNIAVPPCPSLVKICHIAF<br>SGHGRMRMRVPDGQQVRQ<br>SSGH<br>ARCATAASGSSTLPCSGAM<br>LDHGSSGVAYASGDEEPSH<br>LVLIDDDNFRQSPLLGSSDF<br>QG<br>FPFAASEQYTELEAQVRTTE<br>QQDIAVPQLMVQQSTMQV<br>AQQLSAGELEFWSSIGVDV<br>QSN<br>NGFDQEQSTGVQSSWVVPN<br>NNAMATGAQSGWVAPNTG<br>AMATGVQSSWVVPNNGAM<br>ATGAQ<br>SSWVVPNTGAMATGVQSS<br>WVVPNTGAMATGVQSSWV<br>AGDLDDFCRSILTNVQTNC<br>AAPDF

Sspon.05G0006290-1A	*SsNAC065*	ATGGACGCTTTCACACATGTTCCT<br>CCTGGCTTTCGTTTCCACCCTACC<br>GATGAGGAACTC<br>GTTGATTACTACCTTAGGAAAAA<br>GGTAGCACTGAAGAAGATAGACT<br>TGGATGTTATAAAA<br>GATGTGGATTTGTACAAAATTGA<br>GCCTTGGGATCTGCAAGAAAAGT<br>GCAGGATTGGATCT<br>GAAGAGCAGAATGAGTGGTACTT<br>CTTCAGCCATAAGGACAAGAAGT<br>ACCCAACCGGCACT<br>CGCACCAATAGAGCGACGACGGC<br>CGGTTTCTGGAAGGCCACAGGGA<br>GAGACAAGCCGATC<br>TATGTGAAGAACTGCCTAGTAGG<br>GATGAGGAAGACATTGGTTTTCT<br>ACAAAGGTCGGGCG<br>CCTAACGGACAGAAGTCAGACTG<br>GATCATGCACGAGTATCGCCTGG<br>AGACCAACGAAAAT<br>GTAATTCCACACGAAGAAGGATG<br>GGTTGTCTGCAGGGTGTTCAGGA<br>AGCGACTCGTGACT<br>GTCCAAAGAATGGTTGGGGACTC<br>GCCTTATTGGTTCAACGACCAGGC<br>AGGGTTCATGGCG	MDAFTHVPPGFRFHPTDEE<br>LVDYYLRKKVALKKIDLDV<br>IKDVDLYKIEPWDLQEKCRI<br>GS<br>EEQNEWYFFSHKDKKYPTG<br>TRTNRATTAGFWKATGRD<br>KPIYVKNCLVGMRKTLVFY<br>KGRA<br>PNGQKSDWIMHEYRLETNE<br>NVIPHEEGWVVCRVFRKRL<br>VTVQRMVGDSPYWFNDQA<br>GFMA<br>SDLGSSRQAMHHQQSSMM<br>YHRQQSSYSYPCKVELEYH<br>HLLPQEHFLQQLPQLESPKL<br>PDP<br>IGQVDTTLQPCGLTQEHDAP<br>RYTIQELQAEPLYLTVGDAS<br>GTDWRALDKFVASQLSNG<br>DT<br>TPKESASYSNPAHMFQQSE<br>EKEEALDYVS

Sspon.05G0007380-2C	*SsNAC066*	ATGCCACCGAAGAAACCGGCGCC<br>GGGAGCCACTGATCCGGCCGCGT<br>CCGCCAGCCTCCGC<br>GAGGAGGAGACCGACGACGGCTG<br>GGTCCTCCTCGGCGGCGGCGGCG<br>GGGTCAAGCCGCCG<br>AGGCCACCGCCGCGGCGCGGCAC<br>CAGTAGGCCTTCGGCGGGCGGTG<br>GCCATCAGCCGCCG<br>TTCGACCCGGCCCCGGACGACCT<br>CTTCGGGCGGTACCTGCCGGCGC<br>GCCGCGCCCTGCGG<br>TGCGGCGAGCTGCAGCCGCAGAT<br>CCACGACACCGACGTCTATGGCG<br>CGCACCCGGCGTTC<br>CTCGCCAAGGTCCACCCGCCTGCC<br>AGCGCCAGCGGCGGCGGCGAAGG<br>CGACCGGCTCGAG<br>TGGCTCTTCTTCGTCTGCCGCGGG<br>CGGGGCCTCGGCGGCAAGCGGAG<br>GGCGGGGCGCGGC<br>GCGTACCGCCTCGTCGGCGAGGC<br>CAACTCCCGCGGCGGCGGTGCGT<br>GGTACTGCCACTCC<br>TTCCGCTACCACGAGGACGCGGC<br>GGGGGCCAGCGCGTCCAGGGAGA<br>CCGAGTGGCGCATG	MPPKKPAPGATDPAASASL<br>REEETDDGWVLLGGGGGV<br>KPPRPPPRRGTSRPSAGGGH<br>QPP<br>FDPAPDDLFGRYLPARRAL<br>RCGELQPQIHDTDVYGAHP<br>AFLAKVHPPASASGGGEGD<br>RLE<br>WLFFVCRGRGLGGKRRAG<br>RGAYRLVGEANSRGGGAW<br>YCHSFRYHEDAAGASASRE<br>TEWRM<br>DEYGDRGCDSGGAGAGAF<br>DMVVCKVYPARGGALHER<br>LVQQHSTSTPPAAKAKRAA<br>GAADV<br>RPQVLVQLYLASRSVGDPL<br>RCRMHHAADVCAAHPAVL<br>TGVLPAANDRLEWFFAHGG<br>GDDG<br>VIARPRRAGPGQYVPAARY<br>WGVRDDGEGRDVGYRRVF<br>LYREDDEAVRRLSRTEWW<br>MEEYG<br>FGPDFPQRRGAGGVQGVPQ<br>NGPAVGHTPLGLSPKARRL<br>TIAATNMLPIDEDIWDSPLSS

Sspon.05G0008880-2C	*SsNAC067*	ATGGATCATAATGGGACACAATG<br>TGGCAATGATTGGGGAAATTCGT<br>GGATTATGAATGTG<br>TTGGGTTTTGCTGAGAAAATAAG<br>AAGCGCAACTCAGTCGTTCACCCT<br>CCGACTTGGTGAA<br>CTTTTTGCAAAACCACATATCAAA<br>TGTACCAACTGCGACTGCGACATT<br>GACATTAGTAAT<br>GTTTCTTTGGTGTGGCCATCACTT<br>CCTGCTGGTGTCAAATTTGATCCC<br>ACGGACCTTGAA<br>CTTCTGCAGCATCTACAAGAAAA<br>ATCCAGCCCACCGAATTCAGTGTC<br>CCATTCGCTTATT<br>GATGAATTTATACCTACTATAAA<br>AGAGGAGGAGGGAATTTGCTATA<br>CACATCCTAAAAAT<br>CTCCCTGGTATGAAGACGGATGG<br>TAGTTGCCTCCATTTCTTTAATAG<br>AGTATTAAACGCA<br>TATGAAACTGGTAAGCGCAAGCG<br>TCGAAGGATTATTGCCACCGGCG<br>ACATTTGTGACGGT<br>GTTGCTAATGGGAACAGCAGGTG<br>GCATAAGACCGGATCATCCAAAC<br>CTGTATTTGACGAG	MDHNGTQCGNDWGNSWI<br>MNVLGFAEKIRSATQSFTLR<br>LGELFAKPHIKCTNCDCDID<br>ISN<br>VSLVWPSLPAGVKFDPTDL<br>ELLQHLQEKSSPPNSVSHSLI<br>DEFIPTIKEEEGICYTHPKN<br>LPGMKTDGSCLHFFNRVLN<br>AYETGKRKRRRIIATGDICD<br>GVANGNSRWHKTGSSKPVF<br>DE<br>NGVRKGWKKILVLYKAPK<br>KVGGKPERKNWVMHQYHL<br>GVNEDETNGELVVVKVFY<br>QSPLKK<br>NDNSKTDVAVESDASVAKI<br>NPRTPMTNPPQPRRLNNSPC<br>NTEQYTPIQEDQGEEECGTS<br>K<br>MKVEAAECSACFAELSPAM<br>PTSHELQMTPMTDPPQPHR<br>LNNSPCNTEQYTHIQVDQG<br>EEE<br>CSTSKVKVEAAECSACVAE<br>LPPAIPTSDEPMQSADAMD<br>GGLDASLPVDGPSMELFDG<br>LPD

Sspon.05G0008900-2D	*SsNAC068*	ATGGGCCGCGTCCGGCTGCGGTTT<br>CCCGGCCAAAAGGCAAGACATTT<br>TGCAGCCTGCGGA<br>GGTGCAACAGGAAGCGTCGTCCG<br>CTGTGAGGAAGCAGAGGAAGGCT<br>CTGTTTGCTGCAGG<br>CTGAAGTCTGGAGATGGATCGTG<br>CACGCCGGCTGAGCTCCAACGAT<br>CTGTGATCGTTGTG<br>AGGAACAGAGGGGATTTGGCATC<br>TAAAATAAGAAGCGCAACTCAGT<br>CCTTCACCCTCCGA<br>TTTGGTGAACTTTTTGCAAAACCA<br>CATATCAAATGCACCAACTGCGA<br>CTGTGACATTGAC<br>ATTAGTAATGTTTCTTTGGTCTGG<br>CCAGCACTTCCTGCTGGTGTTAAA<br>TTTGATCCCACG<br>GACCTAGAGCTTCTGCAGCATCTA<br>CAAGCAAAATCCAGCCTGCCGAA<br>TTCAGTGTCCCAT<br>ACGCTTATTGATGAATTTATACCT<br>ACTATAAACGAGAAAGAGGGAAT<br>TTGCTATACACAT<br>CCCAAAAATCTCCCTGGTATGAA<br>GATGGATGGTAGCTGCCTCCATTT<br>CTTTAATAGAGTA	MGRVRLRFPGQKARHFAAC<br>GGATGSVVRCEEAEEGSVC<br>CRLKSGDGSCTPAELQRSVI<br>VV<br>RNRGDLASKIRSATQSFTLR<br>FGELFAKPHIKCTNCDCDIDI<br>SNVSLVWPALPAGVKFDPT<br>DLELLQHLQAKSSLPNSVSH<br>TLIDEFIPTINEKEGICYTHPK<br>NLPGMKMDGSCLHFFNRV<br>LNAYDTGKRKRRRIIATSDI<br>CDGVANGNSRWHKTGSSK<br>PVFDENGVRKGWKKILVLY<br>KAP<br>KKVGGKPERENWVMHQYH<br>LGVKEDETNGEFVVCKIFY<br>QSPLKKNDKSKTDVAVESD<br>ASVA<br>KINPRTPVTNPPHPRRLNNS<br>PCNTEQYTLIQEDQGEEECG<br>PSKMKVEAAECSACFAELS<br>P<br>AMPTSHELQRTPMTDPPQP<br>HRLNNSPYNTEQHTHIQVD<br>QGEEECSTSKVMVEAAECS<br>ACV<br>AELSPAIPTSDEPMQPADAL

Sspon.05G0009100-1A	*SsNAC069*	ATGGCGCAGACGCCGGAAGACAC<br>CACTCCTCCCGTCCAGCAGGCTCC<br>GGACAGGGTGCCG<br>CGGCCCATCTCGGAGCTGCCCGC<br>CGGCTACGTCTTCCATCCCAAGGG<br>CGGCAGCCTCCTC<br>AAGAGCTACCTCATCGCCAGGGC<br>GCGCGGTGGCGCCATGAAGGACC<br>CTTACGGGAACACT<br>GTCGCCGACGGCGTCGACGTCTA<br>CGCCGTGCGGCCGGAGGCGCTCC<br>CGTTCCCGCGCCGC<br>AACAGGGGCCTGCACGGCCATGT<br>CTGGGCCTACTTCTTCGCCACGCG<br>GCCCGCGGGCGCG<br>GGAGGAGGAATCGGCGCGGCGGA<br>GGGCGGCGACGAGGACGAGGACG<br>ACGACGTCCGGGAC<br>GTGGCGGCGGGCGGGTGCTGGCG<br>GCGGTACGGCGGCGGCGACAAGG<br>AGTACATCGGCCGC<br>GACGGGGAGGTCTACGCGTTCCG<br>CCGCAGGTTCGCGTTCCACGAGG<br>CCGGGGCCCGCAGC<br>AAGAAGACGGTGTGGAGGATGAA<br>GGAGTTCCGCCTCAAGGAGACCG<br>CGCCCCGCTTCCGC	MAQTPEDTTPPVQQAPDRV<br>PRPISELPAGYVFHPKGGSL<br>LKSYLIARARGGAMKDPYG<br>NT<br>VADGVDVYAVRPEALPFPR<br>RNRGLHGHVWAYFFATRP<br>AGAGGGIGAAEGGDEDEDD<br>DVRD<br>VAAGGCWRRYGGGDKEYI<br>GRDGEVYAFRRRFAFHEAG<br>ARSKKTVWRMKEFRLKET<br>APRFR<br>GVVFHPDAKGLVIWKVWN<br>QVIPEEEPAVDYYGNGGMN<br>DDEEEEEEIGPVVIT

Sspon.05G0010800-1A	*SsNAC070*	ATGGCGGCCTTCTCATCCAGCAAC<br>GGCGTGCCGCCGGGCTTCCGGTTC<br>CACCCGACCGAC<br>GAGGAGCTGCTGCTGTACTACCTC<br>AAGAAGAAGGTCGGATTTGAGAA<br>GTTCGACCTCGAG<br>GTCATCAGGGAGGTCGACCTCAA<br>CAAGATCGAGCCGTGGGAGCTGC<br>AAGAGAGGTGCCGG<br>ATCGGGTCGGCGCCGCAGAACGA<br>GTGGTACTTCTTCAGCCACAAGG<br>ACCGCAAGTACCCG<br>ACGGGGTCGAGGACGAACCGGGC<br>CACGACGGTCGGCTTCTGGAAGG<br>CCACGGGCCGGGAC<br>AAGTGCATCCGCACCAGCTACCG<br>CAAGATCGGCATGCGAAAGACGC<br>TCGTCTTCTACCGC<br>GGCCGCGCCCCTCACGGCCAGAA<br>GACCGACTGGATCATGCATGAGT<br>ACCGCCTCGAGGAC<br>GCCGACGACGCCCAGGGCGGCAC<br>CAGCGAGGACGGATGGGTGGTGT<br>GCCGCGTGTTCAAG<br>AAGAAGTGCTTCTTCAAGATCGG<br>TGGCGGCGAAGGGAGCACCAGCC<br>AGGGCGCGGATGCT	MAAFSSSNGVPPGFRFHPTD<br>EELLLYYLKKKVGFEKFDL<br>EVIREVDLNKIEPWELQERC<br>R<br>IGSAPQNEWYFFSHKDRKY<br>PTGSRTNRATTVGFWKATG<br>RDKCIRTSYRKIGMRKTLVF<br>YR<br>GRAPHGQKTDWIMHEYRLE<br>DADDAQGGTSEDGWVVCR<br>VFKKKCFFKIGGGEGSTSQG<br>ADA<br>GDHLAVSPPLGGHHDQARA<br>ALAYMHPHPYYHHASSYYS<br>QMQAPAPHAAAYSHHVQV<br>QDLL<br>TNHRPTDDGAGTGYDFSGL<br>PVEHHPGGGLDVGSSDGVA<br>TDGGQLAGEGRDQTTGTA<br>AEQW<br>QAMDGFSNGGSAAVQQMT<br>GGMSSGGAQRGGEMDLWG<br>YG

Sspon.05G0013150-1A	*SsNAC071*	ATGGGGCTGAGGGAGATCGAGTC<br>CACATTGCCGCCGGGGTTCAGGTT<br>CTATCCCAGCGAC<br>GAGGAGCTGGTGTGCCACTACCT<br>CTATAAGAAGGTGGCCAACGAGC<br>GCGCCGCGCAGGGG<br>ACGCTGGTGGAGGTCGACCTGCA<br>CGCGCGCGAGCCATGGGAGCTTC<br>CAGACGCGGCGAAG<br>CTGACGGCGAGCGAGTGGTACTT<br>CTTCAGCTTCAGGGACCGCAAGT<br>ACGCGACGGGGTCG<br>CGCACGAACCGCGCCACCAAGAG<br>CGGCTACTGGAAGGCCACCGGCA<br>AGGACCGCGAGGTG<br>CGCAGCCCGGCCACCCGCGCCGT<br>CGTCGGCATGAGGAAGACGCTCG<br>TCTTCTACCAGGGC<br>CGCGCCCCCAACGGCGTCAAGTC<br>CTGCTGGGTCATGCACGAGTTCCG<br>CCTCGACTCGCCG<br>CATACGCCACCAAAGGAGGACTG<br>GGTGCTTTGCAGGGTGTTCCAGA<br>AGCGGAAAGACAGC<br>GAGCAAGACAACGGCGGCTCCTC<br>GTCGCCGACCTTTGCCGGCGCATC<br>GCAGTCGCAGGGG	MGLREIESTLPPGFRFYPSDE<br>ELVCHYLYKKVANERAAQ<br>GTLVEVDLHAREPWELPDA<br>AK<br>LTASEWYFFSFRDRKYATG<br>SRTNRATKSGYWKATGKD<br>REVRSPATRAVVGMRKTLV<br>FYQG<br>RAPNGVKSCWVMHEFRLD<br>SPHTPPKEDWVLCRVFQKR<br>KDSEQDNGGSSSPTFAGAS<br>QSQG<br>VLPDQPSMMDASYVVDQP<br>GSSVGFAPPPQENLNLGGSL<br>DALLMNGAMWQYSSSSVF<br>DHFP<br>QQDVISSPMMGLGSRGGGG<br>DGCSFFYDSGFEDMANIGG<br>M

Sspon.05G0015240-1A	*SsNAC072*	ATGGGAAAAGGAGGAGATGCGAT<br>GAGGGTGGAGGTGGAGAGTGTTG<br>CGAGGGAGGTCCTT<br>TTGGACGCTGCCGTAGGCCGCCG<br>CCCTGCTAGCCGGGGAGCGACGG<br>CGATGGCTGGAGAG<br>CATGTGCGGCCCGCCTCGGCCTTC<br>AGCCCGTCACTGCTTAACCGTGA<br>ACTGGAGAAACAG<br>CTGCTTGCTTGGGGTGGTGTTCTC<br>CGACCTCACTCCTCGCTCGATCCC<br>CTCTCCTCCGGC<br>CCTCAACCGCAGCGATCGGATCG<br>GAGCTGCATCGCGAGGAGCACCG<br>CCGACGCCATCCTG<br>CTGTTTTTACCTGTAAATTCTTAT<br>TTCAATCTTCTCCTCATCCATCTT<br>TTCTTGCTCAGG<br>GGAGATTCAGGCGCCATGATCAG<br>CAGCAACGGCACCGACGCCACAG<br>CTTGCCTCGCCTGC<br>CCTAACTGCAATCTGAACTTGGCT<br>GGATTTCCAGCCGGTCTCAGGTTT<br>GCTCCGTCAGAC<br>CAGCAGCTCATAGAGCACCTAGA<br>GTCCATGGTGTTGGTGAAGGAAG<br>GAGCAGGCTCCAGA	MGKGGDAMRVEVESVARE<br>VLLDAAVGRRPASRGATA<br>MAGEHVRPASAFSPSLLNR<br>ELEKQ<br>LLAWGGVLRPHSSLDPLSS<br>GPQPQRSDRSCIARSTADAI<br>LLFLPVNSYFNLLLIHLFLLR<br>GDSGAMISSNGTDATACLA<br>CPNCNLNLAGFPAGLRFAPS<br>DQQLIEHLESMVLVKEGAG<br>SR<br>AHALLHHFIPTIEGGNDICYT<br>HPENLPGVTRDGLSKHFFH<br>RTSKAYPTGTRKRRKILSES<br>GQRSNEDVGEAHWHKTGK<br>THPVIVGGRQKGCKKILVL<br>HNIKQGMREKTNWVMHQY<br>HLGMS<br>EEENDGELVLSKVFYRCPD<br>ATMVEQNDEKVEVTSEATP<br>NILPVSGAAAVTAATVTMV<br>QQQ<br>QHQLQRQAHGHDQCKFAPP<br>NVFQEAGVGDLVSGDQGQ<br>VHGNNHHIPSQHLVQSVRM<br>EPDT<br>TLSLQVGVGVSGDQGQVH

Sspon.05G0019180-1A	*SsNAC073*	ATGATGGAAAGCAGTGGTGGTAG TGGCGGCGGCAGTGATCCGCTGC TGGCGCCGGGATTC CGATTCTACCCGACGGAGGAGGA GCTGCTGACGTTCTACCTCCGGCA CCGGCTGGCCGGC ACCAGGCCCGTCGTGGAGCACCT GATCCCCGTCGTCGACATCTACGG CTACCACCCATCC GAGCTCCAGGCGCTGGCCGGCGC GGCGAACGTGGCCGACACGGAGC AGTGGTTCTTCTTC TGCCCGCGCGCGGAGCGGGAGCT CCACGGCGGCCGGCCCGTGCGCA CCACGCCGTCCGGC TACTGGAAGGCCACGGGGTCGCC GTCCTACGTCTACTCCTCGCCCAC CGCCAATAACCGG GTCATCGGGGAGAAGCGCACCAT GGTCTTCTACCAGGGCCGCGCGC CCACCGGCAACAAG ACCCGCTGGAAGATGAACGAGTA CAAGGCCCAAGTATTAATGATGC AACTGCTAGTGATC AGTAGTATCTGCCATGTCTGCATG CAGCTGCGGAACGAGTTCAGCGT CTGCCGGGTGTAC	MMESSGGSGGGSDPLLAPG FRFYPTEEELLTFYLRHRLA GTRPVVEHLIPVVDIYGYHP S ELQALAGAANVADTEQWF FFCPRAERELHGGRPVRTTP SGYWKATGSPSYVYSSPTA NNR VIGEKRTMVFYQGRAPTGN KTRWKMNEYKAQVLMMQ LLVISSICHVCMQLRNEFSV CRVY ISTGTLRSFDRRPLNPTGGL AVAAVHCGYQQQQQQLQP LPPPAAVAETTTSHHHQQM LAT VVVDDSAADNNGRRHAAA AAEKSQDSSSSGSRGGGGV DGAEDAATVIDWDSLIPPV DELA FGGVDDLTRVIWPHN

Sspon.05G0019300-1A	*SsNAC074*	ATGATTCCCATCGTGGACATCGGC<br>TGCCTCCTCGCCGCGCACGCGCCT<br>CGCTCGCAGCTC<br>GTCCAGAGCCGCGTGGACCGCGC<br>CGGGCAAGGCTCAGTGGGCATCA<br>CGGTCACCGCGATC<br>CCGTTCCCGGGCGCGGAAGCCGG<br>CCTGCCGGATGGCTGCGAGAGGA<br>CCGACCACATCCCT<br>TCGGCTGACCTCGTGCCCAACTTC<br>TTCGTCGCCACCACCAACACTGCC<br>GCCTCCTCACGG<br>CGACGCACCGGCCGAGCTGCGTC<br>GTCGCCGGGATGGGCCACACGTG<br>GGCGCACGGCGTGG<br>CGTGAACTCGGTGCGCCTTGCTTC<br>ATCTTCCATGGCTTCTCTGCGTTC<br>GCTCTGCTGTGC<br>TGCGAGTATCTGAACACGCACAA<br>ACCGCACGAGGCAGTCGAGTCGC<br>CGGACGAGCTCTTT<br>GACGTCCCGTTCGCCAGGAGGCA<br>GCTGCCGATACACTTCCAACCTTC<br>TTCCTCCATTCCG<br>CAAGACCACTGTCGAGAGCTCCG<br>GGAATTCGAGCTGGCCCTTCGAG<br>GAGCTGGAACACGA	MIPIVDIGCLLAAHAPRSQL<br>VQSRVDRAGQGSVGITVTAI<br>PFPGAEAGLPDGCERTDHIP<br>SADLVPNFFVATTNTAASSR<br>RRTGRAASSPGWATRGRTA<br>WRELGAPCFIFHGFSAFALL<br>C<br>CEYLNTHKPHEAVESPDELF<br>DVPFARRQLPIHFQPSSSIPQ<br>DHCRELREFELALRGAGTR<br>LGRALAAATGKVVLTVGPV<br>SLCSAPPSLLDLRASSDDAR<br>RCMAWLDAKKAESVLYVS<br>FGS<br>AGRMLPAQLMQLGLALVS<br>CPWPVLWVIKGADTLPDDV<br>SEWLQHNTDGDGIPESQCL<br>AVAI<br>LEHPAVGGFLTHCGVASGV<br>PMATWPFFAEQFLNEKLIV<br>DVLGIGVSVGVTKPTASVL<br>TGG<br>KDGGGGDKAKADQVLDML<br>MGGGVDGEARKTKAKELK<br>AKAKTALEHGGSHLPFWV<br>VGDGGG<br>PCAEILSRGFRFLPTPQEAVT

Sspon.05G0020040-1A	*SsNAC075*	ATGGAGGAGGCGCCGCCGCGGTG GCCGAACGTTAACGCGCCCCCAG GGTACCGCTTCAAG CCGACCCCGCGGGAGCTGATCCA GTGCTACCTCGAGCCCTGGGTGG CCACCGAGCCCGGC CGCCAGTCGCCCGGCGAGTTCTA CGGCATCATGGCCGCCGCGGACG TGTACGGCGAGGAC CCCGGCGCGCTGGCGTCGCGCTTC CAGCACATCGCCCACGACGACGG CAACTGGTACTTC CTCTCCGTCGCCCGCTGGAAGGA CGGCAACGCCAGCAGCAAGCGGA TGAACCGCGCCGTC GGGGCACTCGGCACGTGGCACGG GTCCGGCAAGCGGATCCCCGTCC GCGGCGCCGGGTAC CGCCAGTCCTTCGAGTTCCGCCCC GCCGGCGGCGGCAAGGCCACGTG GCTCATGGAGGAG TTCGGCACCGTCCGGAACGACGC CACCGGCGAGGACGGCGTCAGGG TGCTCTGCAGGCTG CACCTCAGGCCCAAGGCGGCGGG CGCGGCGGACGGCGACCAGCACC AGCAGCAGCTCGAG	MEEAPPRWPNVNAPPGYRF KPTPRELIQCYLEPWVATEP GRQSPGEFYGIMAAADVYG ED PGALASRFQHIAHDDGNWY FLSVARWKDGNASSKRMN RAVGALGTWHGSGKRIPVR GAGY RQSFEFRPAGGGKATWLME EFGTVRNDATGEDGVRVLC RLHLRPKAAGAADGDQHQ QQLE ANDVPAPCNKRQRQRAAA RQVEYHFDADYWTAAPDV GLSSSYATATAPAPPDVGCS SYAT TSSHAAAVPVTELTTEALV TWQHQPMMEQAGDGGYQ YHCAGVHGGVYIRVDDEPQ RLEMV TEDVEFTTQDLKLEDSDFVF TVEHLLQLDDGWIMDSNSN AFSVLHPMCDGVQENIDPK PE PSD

Sspon.05G0020640-1A	*SsNAC076*	ATGGTGGAGCGTAGTGTGAAATC AGAGCATGGAGTCGACCTGTTCC TGCCTCCTGGCTTC AGGTTTCATCCCACAGACGAAGA GGTCATCACCAGCTACCTCCTGCA GAAGTTCTTGAAC CCTAGCTTCGCGCCGCACGCCATC GGGGAGGTGGACCTCAACAAGAT CGAGCCATGGGAT CTCCCAACGAAGATGGGGGAGAA GGAGTGGTACTTCTTCTGCCACAA GGACATGAAATAC CCGACGGGCACGCGCACGAACCG CGCCACCAAGGAGGGCTACTGGA AGGCCACCGGCAAG GACAGGGAGATCTTCAAGCAGCC TGGCCGTGAGCTGGTGGGGATGA AGAAGACGCTGGTG TTCTACATGGGCAGGGCTCCGCG GGGCACCAAGACCAACTGGGTGA TGCACGAGTTCCGC CTCGACGGCAAGTCCAGGCACAC CAACGACTCCAACCTACGCTTCA ATCCCAAGGATGAA TGGGTCGTGTGCAAGGTGCACCA CAAAGGGGAAGAAGAGGCCAGC AGTAAGAAGACCGCC	MVERSVKSEHGVDLFLPPG FRFHPTDEEVITSYLLQKFL NPSFAPHAIGEVDLNKIEPW D LPTKMGEKEWYFFCHKDM KYPTGTRTNRATKEGYWK ATGKDREIFKQPGRELVGM KKTLV FYMGRAPRGTKTNWVMHE FRLDGKSRHTNDSNLRFNP KDEWVVCKVHHKGEEEAS SKKTA AGEEQHSSAAGTPNVSSVE VGEGGDEFLVDSLLDYSSY FNSAAPPYNADCCYPVHTT AAT GMTTTTPTTTTSSCFVGLPT DASNSQHAAAVANSAAAA ATNNNDSSSWNMLRHAPD QQAM GTNYSLQHQAMVAKALGG GGVATPNFGAGLPAGSSVA AAAGIAQHSSQNVMLQQRL AGYY GGNYAGGYHTSK

Sspon.05G0020650-1A	*SsNAC077*	ATGGCCATGCCGAGCCCCAGCCC CAGCCCCAGCCTTAGCAGCGGTA GCTGCAGAGTGGCT CCAAACCTAAACCTCCCAGCCGG GTTCCGTTTCCACCCCACGGACGA GGAGCTCATCGTC CACTACCTCATGAACCAGGCCGC CTCCATCCCCTGCCCCGTCCCCAT CATCGCCGAGGTC AACATCTACCAGTGCAACCCTTG GGATCTCCCCGCCAAGGCGGTGT TCGGCGAGAGCGAG TGGTACTTCTTCAGCCCGCGGGAC CGCAAGTACCCCAACGGCGTGCG ACCCAACCGCGCC GCCGGCACGGGCTACTGGAAGGC CACCGGCACCGACAAGGCCATCA TGTCCACGCCCACC GGCCAGCATATCGGCGTCAAGAA GGCCCTCGTCTTCTACGGCGGTCG GCCGCCCAAGGGC ATCAAGACCGACTGGATCATGCA CGAGTACCGCCTCGTCACCGCTGC CGACGGCAAGACG GCCGCGGCAGCCAAGCGCAGAGG AGGATCCATGAGGCTGGACGACT GGGTGCTGTGCAGG	MAMPSPSPSPSLSSGSCRVA PNLNLPAGFRFHPTDEELIV HYLMNQAASIPCPVPIIAEV NIYQCNPWDLPAKAVFGES EWYFFSPRDRKYPNGVRPN RAAGTGYWKATGTDKAIM STPT GQHIGVKKALVFYGGRPPK GIKTDWIMHEYRLVTAADG KTAAAAKRRGGSMRLDDW VLCR IHKKSNDFQLSSDHQEQEQE QEGSSTVEQESPKPEPLEHD QFQFHQTTLTKSCSLTDLLN NFDYSALSQILDGPVDGSEA LPQQNPPLPNYPTTTHETHQ ALNYNNNNNNLNNNNNHV IS LPHTNATACSADSIVANNC NNGLNKRKRMTTDAMNDG VESFDYGRNGFSRKPKVLP TDSR NSSHLGSTSSSSYCNQQVV DTSGLFHYSSLLSYPFVANN AGTS

Sspon.05G0023210-1B	*SsNAC078*	ATGAGCTTGATCAGCATGATGGA<br>GGCGCGGCTGCCGCCGGGGTTCC<br>GGTTCCACCCCAGG<br>GACGACGAGCTCGTGCTGGACTA<br>CCTCTGCCGCAAGCTCTCGGGCG<br>GTGGTGGCGGCGGT<br>GCGGCGTACAGCGGCATCGCCAT<br>GGTGGACGTCGACCTCAACAAGT<br>GCGAGCCGTGGGAT<br>CTTCCAGACGAGGCGTGCGTGGG<br>CGGGCGCGAGTGGTACTTCTTCA<br>GCCTCCACGACCGC<br>AAGTACGCGACGGGGCAGCGGAC<br>CAACCGCGCCACGCGCTCCGGGT<br>ACTGGAAGGCCACG<br>GGCAAGGACCGCCCCATCTCCAT<br>CTCCGGCCGCCGCGGAGGAGGCA<br>CGGGCGCCGGCGCG<br>CTGGTCGGGATGCGCAAGACGCT<br>GGTGTTCTACCAGGGCAGGGCGC<br>CCCGCGGCACCAAG<br>ACCGAGTGGGTCATGCACGAGTT<br>CCGCGTGGACGGCCCGGCCGTTG<br>CCGACCGCCCCGGC<br>TCACCTCTCCTGCAGGAGGATTGG<br>GTCCTGTGCAGGGTGTTCTACAAG<br>AGCCAAACTACC	MSLISMMEARLPPGFRFHPR<br>DDELVLDYLCRKLSGGGGG<br>GAAYSGIAMVDVDLNKCEP<br>WD<br>LPDEACVGGREWYFFSLHD<br>RKYATGQRTNRATRSGYW<br>KATGKDRPISISGRRGGGTG<br>AGA<br>LVGMRKTLVFYQGRAPRGT<br>KTEWVMHEFRVDGPAVAD<br>RPGSPLLQEDWVLCRVFYK<br>SQTT<br>TTRPAAGPDEAGSLSTSSELI<br>GLPMPQMAPADDAYLSFDN<br>TPAAGGYYDYHQDPGLAD<br>AH<br>HLPLPAATQPFSQSSSLSSFR<br>DLLSSMVEGSDAAVRETTE<br>LHLQGWTEAAYAQQQGGV<br>MS<br>SRSQLTWN

Sspon.05G0024050-1B	*SsNAC079*	ATGGCAGAAGGAGAGGAGAGACC<br>GAGCGGAGGGGGTGACGACGAGA<br>ACGGCAAGTACCCG<br>ATCGGGTTCCGCTTCAAGCCGAC<br>GACCGAGGAGCTCGTGGAGTTCT<br>ACCTCCTCCCCAAA<br>CTGTTGCACGAACCGACCGTGCC<br>AAACGAATTCGTCATCGAGGCGG<br>ACGCGTACGGGTGT<br>GACCCGGAGATATTGACTGAAAA<br>ATACAAGGAGAGCGGCGTGGACG<br>ACACCTGGTACTTC<br>CTGTCGCCGAGGTACCGCAAGTA<br>TCGGGGAGGGGATAGGCCGGTGC<br>GGCGCACGGCCGAC<br>GACCGTGGCCGGTGGAAGCCGTC<br>GACGGGCCAGTCGAAACCACCGG<br>AGGAGGAGAACGAC<br>GCCTCGACGAGCCATTCCAAGGC<br>GAAGAAGGCCGCGTTCAGCGAGA<br>ACACCCTCGCCTAC<br>TACGTAGGCCCCACCAAGGACGA<br>GACCAAGACCAAGTGGCTCATGC<br>ACGAGCTCGTCGTC<br>CCCGAGATGCCCGACAACGGCTT<br>[illegible]	MAEGEERPSGGGDDENGK<br>YPIGFRFKPTTEELVEFYLLP<br>KLLHEPTVPNEFVIEADAYG<br>C<br>DPEILTEKYKESGVDDTWY<br>FLSPRYRKYRGGDRPVRRT<br>ADDRGRWKPSTGQSKPPEE<br>END<br>ASTSHSKAKKAAFSENTLA<br>YYVGPTKDETKTKWLMHE<br>LVVPEMPDNGFHSKSAAAE<br>PRDD<br>MM

Sspon.05G0025100-2C	*SsNAC080*	ATGACACCGACATTTGTTTCGCTC<br>TCACAAAAAGAAGGCGGCGCCAG<br>CGGCGGCGGCACC<br>TCGGACGATCGCGTCGGGCGCTT<br>GGTTGATGGGTACCTCGAGACGG<br>AGACGGGGAAGAAG<br>GCGATGCGGCGGCCGTCTCCGGC<br>GACAGCTTCAGCTTCCCCTGTTCG<br>GGTGACTCCGAAG<br>ATGGCCGAACATAGATTTATCGTT<br>CTTGAAGAACTTCCCGCACTCGG<br>GACATTCGACATC<br>CCCGCCGCCGCTACCGGAGCTGC<br>TGCCCTTGGCTCCACCGCCGCCGT<br>TCTCGTCAGGCAA<br>CCACCCCTCTTCCTCCTCCACACG<br>CACGCCTCCACCGTCGCTCGCATA<br>CCCTTCACCCAA<br>TCAGAGCTGTGGCCGTGGAGCTC<br>CTTGCGGCCATGGTTTGACATCAA<br>CCGCGGCGGCCTC<br>CCGATCGGATCTTGGTGGCACAA<br>AAACTTGCCCCCAGCCTGGCCTGC<br>GGTCAGCTGCCAG<br>CTCGAGATCAGGCACAGCTGGAC<br>GATGCAGACTTGCACCAGCACCA<br>GCGAGCTCAGCAGC	MTPTFVSLSQKEGGASGGG<br>TSDDRVGRLVDGYLETETG<br>KKAMRRPSPATASASPVRV<br>TPK<br>MAEHRFIVLEELPALGTFDI<br>PAAATGAAALGSTAAVLVR<br>QPPLFLLHTHASTVARIPFT<br>Q<br>SELWPWSSLRPWFDINRGG<br>LPIGSWWHKNLPPAWPAVS<br>CQLEIRHSWTMQTCTSTSEL<br>SS<br>KLPLLIRRSRAARSATPERW<br>ISGGSSRAERGGLTFYLRLR<br>LRLRLLLWKAQHRYPAGIG<br>T<br>PPKQRWKLKGRGESVILVG<br>TQNLAATPEASIFRFSVSDPS<br>MAQTPEDATAPVQAPRPLP<br>E<br>LPFGCVFHPDGPCLLKRYLL<br>PMALGRDSVADGVDVYAV<br>RPEAPPFPRCNRGLHDKVW<br>DYY<br>FTTTQPAAAGGSGVVGGSE<br>DDVRDVAAGGCWRRYGAE<br>KEYVGDDGEVYGFRRRCAF

Sspon.05G0027840-1B	*SsNAC081*	ATGGGGGCGAGGCCACCTAGCCG<br>CCCGGCGTTCTGCAGCCAGCCGC<br>CGCCTGGCTCTGCC<br>AATGGCCATAGTGCACGTTCACCT<br>ACCGTGCACTGTCACCTAGTAGCC<br>AAATCTAGTGCT<br>GGGATCGCCGGATCCGGTCCCGG<br>GGGCACAGATTTGGCGCGTTGCC<br>TGCCACCCTACTCG<br>CCACTGGCCGTCCTTGCCGCTAAT<br>GTGCCAGAGTTCGACATGGGAAA<br>AGCAGAGGCGATG<br>AGGGTGGAGGTGGAGAGTGCTAC<br>GAGGGAGGGCCTTTTGGACGCCG<br>CCGCGGGCCGCCGC<br>CCTGCTAGCCGGGGAGCGACGGC<br>GATGGCCGAAGAGCATGCACGGC<br>CCGCCTCGGCCTTC<br>AGCCCGTCACTGCTTCACCGTGAA<br>CTGGAGAAACAGCTGCTTGCTTG<br>GGGTGGTGTTCTC<br>CGACCTCACTCCTCGCTCGATCCC<br>CTCCCCTCCGGCCGTCAACCGCAG<br>CGATCGGATCGG<br>AGCTGCATCGCGAGGAGCACCGC<br>CGACGTCATGGGAGATTCAGGCG<br>CCATGATCAGCAGC	MGARPPSRPAFCSQPPPGSA<br>NGHSARSPTVHCHLVAKSS<br>AGIAGSGPGGTDLARCLPPY<br>S<br>PLAVLAANVPEFDMGKAEA<br>MRVEVESATREGLLDAAAG<br>RRPASRGATAMAEEHARPA<br>SAF<br>SPSLLHRELEKQLLAWGGV<br>LRPHSSLDPLPSGRQPQRSD<br>RSCIARSTADVMGDSGAMI<br>SS<br>NGTDATASLACPNCNYLRL<br>NLSSVLSYLKSFDCNKNLA<br>GFPAGLRFAPSDQQLIEHLE<br>SM<br>VLAKEGAGSRAHALLNHFI<br>PTIEGGNDICYTHPENLPGV<br>TRNGLSKHFFHRTSKAYPT<br>GT<br>RKRRKILSESGQRSNEDVGE<br>AHWHKTGKTHPVIVGGRQ<br>KGCKKILVLHNIKQGMREK<br>TNW<br>VMHQYHLGMSEEENDGEL<br>VLSKVFYRCPDATMVEQND<br>EKVEVTSEATPNILPVSGAA

Sspon.05G0029190-1B	*SsNAC082*	ATGGACTGCGGTGGCGCGCTGCA<br>GCTCCCGCCCGGGTTCCGGTTCCA<br>CCCCACCGACGAC<br>GAGCTGGTGATGTACTACCTCCTC<br>CGCAAGTGCGGCGGCCTGCCGCT<br>CGCCGCGCCGGTC<br>ATCGCCGAGGTCGACCTCTACAA<br>GTTCGACCCATGGCAGCTCCCAG<br>AGAAGGCGTACGGT<br>GGGGAGAAGGAGTGGTACTTCTT<br>CTCGCCGCGGGACCGCAAGTACC<br>CGAACGGGTCGAGG<br>CCGAACCGGGCCGCCGGGACGGG<br>GTACTGGAAGGCCACCGGCGCCG<br>ACAAGCCGGTGGGG<br>TCGCCGCGCGCCGTGGCCATCAA<br>GAAGGCGCTCGTCTTCTACGCCG<br>GGAAGCCGCCCAAG<br>GGCGTCAAGACCAACTGGATCAT<br>GCACGAGTACCGCCTCGCCGACG<br>TCGACCGATCCGCC<br>GCCGCACGCAAGAAGACCAACAA<br>CGCGCTCAGGGAGTGGTATTTGTT	MDCGGALQLPPGFRFHPTD<br>DELVMYYLLRKCGGLPLAA<br>PVIAEVDLYKFDPWQLPEK<br>AYG<br>GEKEWYFFSPRDRKYPNGS<br>RPNRAAGTGYWKATGADK<br>PVGSPRAVAIKKALVFYAG<br>KPPK<br>GVKTNWIMHEYRLADVDR<br>SAAARKKTNNALREWYLF<br>VSQMKY

Sspon.05G0029330-1B	*SsNAC083*	ATGGAGGCGTGGCGGTTCGGCTT CGACTCGTCGTTGTTCCCGCCGGC GTACAAGTTCGAC CCCACCGACACCGACATCGTGGT GCACTACCTCCTCCCACGCGCCCT GGGCGTCCGCAAC CCATACGAGCACGCCGTGATCGA CGCCGACCCCTGCAGCTGCCCGCC GTGGGAGTTCATG CGCCGCAACGGCCACGGCGACAG CGACCACGCCTTCTTCTTCGCGCC CCCGCGAGCGCGC GGGAAGAGGGCCGTCCGCGTAGT CTCCCCAGCCCCAGTGGAGGAGG ACGGCAGCGGCGTC GGCGGCAGGTGGGACGCGCAGAA GAGCACGGAGACCTCCATCGTCC TCGTGCGCGGCGGC CCGGGCGGCGGCGCCGAGGTGCC CATCAAGTACAAGCGCTGCAACC TGTCGTACTACCAC GGCGACGAGCCCAGCACCAGCGG GTGGGTCATGCACGAGTACCAGA TCACCGTGCCGCCG CGGCTCTCCCGCACCGTCCTGTCG CGCGTCAAGGTCACCGATAGGGG CAAGCAGCGACTG	MEAWRFGFDSSLFPPAYKF DPTDTDIVVHYLLPRALGV RNPYEHAVIDADPCSCPPW EFM RRNGHGDSDHAFFFAPPRA RGKRAVRVVSPAPVEEDGS GVGGRWDAQKSTETSIVLV RGG PGGGAEVPIKYKRCNLSYY HGDEPSTSGWVMHEYQITV PPRLSRTVLSRVKVTDRGK QRL KDAGGPQQVVVPDPDQPGP SNNDDFVHGHDHDDDDGN ALGLSGGEQSEVVYLGDGN GSVS GEMAAYFVDDGNSYLNDG SGYYHQYQDDVSGGDYGF TVGDNNVNHEYLDRINSPE RFYSS VSFEVTQIKAPEYYIFQGNV VPDSDM

Sspon.05G0032850-1C	*SsNAC084*	ATGGGGGGAGCTTCTAATTCTAA<br>CCTTCCTCCTGGCTTCCACTTCTT<br>CCCATCAGACGAA<br>GAGCTCGTCGACCATTTCCTCCGC<br>CGCAAAGCCTCGCTCCTCCCATGC<br>CAGCCAGACATC<br>GTCCCTACAGTGTGTATGAATCAC<br>TACGATCCATGGGAACTGAATGG<br>CAAAGCACTTGAG<br>GCAGGTAACCAGTGGTACTTCTTC<br>AGCCATGCGAAGCACAGCAGGGT<br>CACACCAAACGGG<br>TACTGGAGCTCTGTTTGTGCAGAC<br>GAAACGGTGAGCAGTGGTGGCTG<br>TAGTGTCGGCGTG<br>AAGAAGACACTCGTCTTCTCCACT<br>GGAGAGCCCTCTGAGGGGACCGA<br>GACCAACTGGATC<br>ATGCATGAGTATCACTTACTGGAT<br>GCCAGGAAGGGGATTAGCAGTAG<br>CACTTCCTCAACT<br>AATAGTTCAAGCAAGAAGTTGCA<br>TCATCCAAACACAGTAAGAGCTA<br>CATGCATATATATG	MGGASNSNLPPGFHFFPSDE<br>ELVDHFLRRKASLLPCQPDI<br>VPTVCMNHYDPWELNGKA<br>LE<br>AGNQWYFFSHAKHSRVTPN<br>GYWSSVCADETVSSGGCSV<br>GVKKTLVFSTGEPSEGTETN<br>WI<br>MHEYHLLDARKGISSSTSST<br>NSSSKKLHHPNTVRATCIY<br>MFAYLLASSS

Sspon.06G0000010-1A	*SsNAC085*	ATGGCTTCCAACCAAGATCGCAG CCCCACCGACAATAAGGCTTCCA TCTCCCTCCTGCGC CGCCTCCGCGCCGGAGACGACGA CGACGTCCACTTCGTCCACCGCGT CGACGTCTGCGCC GCTCCTCCAGAGGTTCTCGTCGCT GATCTGGAGCCGGTGCCGGGAAC CGGTCTCGAAGAC GACGGCTACAGCAGGATCTGGTA CTTCTACCACGCCAAGAAATACA AGAACACCCGAGGC GACACCAGCGGGCACAGGCAGCG CGCGGTCACCGGAGGCGACGGCA CGAGCTGGCACTCG GAGATAAGCCGCAGGGACGTCCA GGGATCCGGTGGCGGCACGTTCT GCACCTTCTCCTAC GGCCGCAAGACGGAGCCCTCCTC TCGATCGATCGACAGGATGGGAT GGTGCATGGCGGAA TACGACTTGGTCGCCGCGGACAG CAGCAACTACGTGCTTTGCAAGG TCTACCGCTCGCCT CGCGCGAAAGGGAAGTCGGCGTT GGCGTCATCCTCCTCTAGCAAGA ACGCCTCGTCCAAG	MASNQDRSPTDNKASISLLR RLRAGDDDDVHFVHRVDV CAAPPEVLVADLEPVPGTG LED DGYSRIWYFYHAKKYKNTR GDTSGHRQRAVTGGDGTS WHSEISRRDVQGSGGGTFC TFSY GRKTEPSSRSIDRMGWCMA EYDLVAADSSNYVLCKVYR SPRAKGKSALASSSSSKNAS SK QTAKKRKAGGGEHPEAPPA KSIQRQEQVQQETAYYPMP NYRYQPEADVQEVAVQQP AGPG GDFNFNIEEINLYVDDECML RADESEQGRRRGLQQSISEP ERHEVEFIRLPCGPVVPVVA EATFEDMLGLPGPETMYC GEWSGGMATARIPLPWSSY APTSSSYTACSGLLQGSDPL LQ LPPYLM

Sspon.06G0001130-1A	*SsNAC086*	ATGGCGGCAGAGAGAGCACCGGT GCTGGTGAGGCACGCCGGAGGCG GCTTGGAGGAGCTC CGGCAGCTCCCGCCGGGGTTCCG GTTCCGCCCCACGGACGAGGAGC TCGTGGTGCAGTAC CTCCGGCGCAAGGCCTTCGGCGT GCCGCTCCCCGCCGCCGTCATCCC CGTCGTCCGCGAC CTCTACAACCTCGACCCCTGGGAC GTCGTCGTCCCTGCCCCTGCCCCT GATGCGAGCAGC GAGGGGGAGAAATACTTCTTCGC AGTCCGGCCGGCGGGCGCCGGCA AGAGCGGCGGTGCT AGGGCGACGGCGAGCGGGCGGTG GAAGCCGTCGGGGAAGGAGAAGC CGGTGGTCCTACCC CGCCCGTGCGGCGGAGGGAGACT CCTGGTGGGCGTGAAGAGGGCGA TGACGTTCGTGCCC CGGCGGAAGAAGAAGGCGTCGCC GTCGGCGTCGGCGGCCCTGGCGA TCGGCTGGGTCATG CACGAGTACCGCCTCGCCGCGCC GCTGCACAAGAACGGCTGCAGCC TGGCTCAAGGAGAG	MAAERAPVLVRHAGGGLE ELRQLPPGFRFRPTDEELVV QYLRRKAFGVPLPAAVIPV VRD LYNLDPWDVVVPAPAPDAS SEGEKYFFAVRPAGAGKSG GARATASGRWKPSGKEKPV VLP RPCGGGRLLVGVKRAMTF VPRRKKKASPSASAALAIG WVMHEYRLAAPLHKNGCS LAQGE WVVCRVFQKGSSRPTRRRR AVPAAHPAAAASPSPSPSSA SSCVTDGSNSDLDEVSG

Sspon.06G0001780-1A	*SsNAC087*	ATGCACCACCACCAGGCCATGAG CGACGCGCTGTGGGACCTGCTCG GGGAGGAGATGGCG GCGGCGGGCGGCGAGCACGGCCT TCCCCCGGGGTTCCGGTTCCACCC CACCGACGAGGAG CTGGTCACCTTCTACCTGGCCGCC AAGGTGTTCAACGGCGCCTGCTG CGGCATCGACATC GCCGAGGTGGACCTCAACCGGTG CGAGCCGTGGGAGCTCCCCGACG CGGCGCGCATGGGG GAGCGCGAGTGGTACTTCTTCAG CCTCCGCGACCGCAAGTACCCCA CGGGCCTCCGCACC AACCGCGCCACCGGCGCCGGCTA CTGGAAGGCCACCGGCAAGGACC GCGAGGTGCTCAAC GCCGCCACCGGCGCGCTCCTCGG CATGAAGAAGACGCTCGTCTTCT ACAAGGGCCGTGCG CCGCGCGGCGAGAAGACCAAGTG GGTCCTCCACGAGTACCGCCTCG ACGGCGACTTCGCC GCCGCTCGCCGCCCCTGCAAGGA GGAATGGGTGATCTGCAGGATAC TGCACAAAGCAGGC	MHHHQAMSDALWDLLGEE MAAAGGEHGLPPGFRFHPT DEELVTFYLAAKVFNGACC GIDI AEVDLNRCEPWELPDAAR MGEREWYFFSLRDRKYPTG LRTNRATGAGYWKATGKD REVLN AATGALLGMKKTLVFYKG RAPRGEKTKWVLHEYRLD GDFAAARRPCKEEWVICRIL HKAG DQYSKLMMVKSPYYLPMA MDPSSFCFQQDPTAPPLQNP SGCIPFQHGHPSMQPPPLPPS N HGKVVFTGAAAPCMQQEP ANGSNSAVLPMPPLPHFTPI VAGKPAPAPPPQVGVNAGP QEP PPPPPTWLEAYLQHGGGFL YEMGPAAAPRGA

Sspon.06G0005910-1A	*SsNAC088*	ATGCAAGGGGAGGAGCAGCAGCA GCGGATTGAGCTTGCCCTGCCGCC GGGCTTCCGCTTC TTCCCGACGGACGAGGAGCTCAT CACCTGCTACCTCGCCAGGAAGG CCATGGACGGCAGC TTCACCACGGCAGCCATCCGCGA CGTGGACCTCTACAAGACAGAGC CATGGGACCTGCCA TGCGAGCAGCAGGCGGCGGCGGT CGGAGGAGACCTGCAGGAGGGCT ACTTCTTCTGCACG AGGGGCAGCAAGTCCCCATCCGG CGTTCGCGCCCGCCGCGCAACAC AGCTGGGCTACTGG AAGTCCACGGGGAAGGACAAGCC CGTGCACAGCAGGTCCGGCCGCC TCGTCGTCGGGACG AGGAAGACGCTCGTGTTCTACCG CGGCAGGGCCCCGAGAGGGGAGA AGACCGACTGGGTG ATGCACGAGTACGCCATGGGCGA	MQGEEQQQRIELALPPGFRF FPTDEELITCYLARKAMDGS FTTAAIRDVDLYKTEPWDL P CEQQAAAVGGDLQEGYFFC TRGSKSPSGVRARRATQLG YWKSTGKDKPVHSRSGRLV VGT RKTLVFYRGRAPRGEKTDW VMHEYAMGERRSSALLRG AQ

Sspon.06G0009610-1A	*SsNAC089*	ATGCCTCCGGGGTTCAGGTTCCAC<br>CCCAGGGACGACGAGCTCGTGCT<br>GGACTACCTCCTC<br>CACAAGCTCTCCGGCCGTGGCCA<br>TCGCGGCGGCGCAGCCATCGTCG<br>ACGTCGACCTCAAC<br>AAGTGCGAGCCATGGGACCTTCC<br>AGAAATTAACAATACAGAGTGGT<br>ACTTCTTCAACCTG<br>CGCGACCGCAAGTACGCGACGGG<br>GCAGCGCACCAACCGCGCCACGC<br>GCTCCGGCTACTGG<br>AAGGCCACCGGGAAGGACCGCAC<br>CGTCGTCTCCGGCGGTGGCGAGG<br>ATGCGGCGGCGGCG<br>GCGGTGGTGGGGATGCGCAAGAC<br>GCTGGTGTTCTACCGGGGCCGAG<br>CACCCAAGGGGAGG<br>AAGACGGAGTGGGTCATGCACGA<br>GTTCCGCCTCCTCCCACACGCCGC<br>GCCGTGCCTACAA<br>CCTGCGGCAACGAAGGAGGACTG<br>GGTGCTATGCAGGGTGTTCTACA<br>AGAGCAGAACCACC<br>CCCCCAAGGCCAGCATCTGAAGA<br>TGCCCAGGACGGCACCCCATCAG<br>CTGAGCCACAACTG	MPPGFRFHPRDDELVLDYL<br>LHKLSGRGHRGGAAIVDVD<br>LNKCEPWDLPEINNTEWYF<br>FNL<br>RDRKYATGQRTNRATRSGY<br>WKATGKDRTVVSGGGEDA<br>AAAAVVGMRKTLVFYRGR<br>APKGR<br>KTEWVMHEFRLLPHAAPCL<br>QPAATKEDWVLCRVFYKSR<br>TTPPRPASEDAQDGTPSAEP<br>QL<br>MAALPLAPLADTYTVYGA<br>APTVSEQVSCFSGLPALPFK<br>RPVSLGDLLTFDTSEKESVR<br>TV<br>MSGVSNNSKFHPLNDTCW<br>KAQDMTIQARRFSRGKGSQ<br>CGRVCIAWSLIVVSIEIIVHK<br>DP<br>MRPFGTATNQVS

Sspon.06G0012880-1A	*SsNAC090*	ATGACACAAGCAGGCCGCCGTCG<br>ATCGAGCTCGACCATGGGGGGCG<br>GAGGATCCGGAGAC<br>CACCAGCAGCAGCATGGCGGAGG<br>CAGTGGCGACGATGGGCAGCTGC<br>AGCAAGGCGGAGGC<br>GACATGGTGATGCCGGGGTTCCG<br>ATTCCACCCCACGGAGGAAGAGC<br>TCATCGACTTCTAC<br>CTCCGCCGTAGGGTGGAGGGCAA<br>GCGCTTCAACATCGAGCTCATCA<br>ACCTCGTCGACCTC<br>TACCGCTACGACCCATGGGATCTC<br>CCTGCTTTGGCTTCGATTGGGGAC<br>AAGGAGTGGTAC<br>TTCTATGTGCCGAGAGACCGCAA<br>GTACCGCAACGGCGACCGGCCCA<br>ACCGGGTCACGCCA<br>TCCGGCTACTGGAAGGCCACGGG<br>AGCCGACCGCACGGTGTACGTCG<br>AGGTCAAGCGGCCG<br>ATCGGGCTCAAGAAGACGCTGGT<br>GTTCTATGTGGGGAAGGCGCCCA<br>AGGGACTCAGGAGC<br>AGCTGGATCATGAACGAGTACCG<br>CCTTCCTTCAGGCGAGGCCGACC<br>GCTACCAAAAGGAA	MTQAGRRRSSSTMGGGGS<br>GDHQQQHGGGSGDDGQLQ<br>QGGGDMVMPGFRFHPTEEE<br>LIDFY<br>LRRRVEGKRFNIELINLVDL<br>YRYDPWDLPALASIGDKEW<br>YFYVPRDRKYRNGDRPNRV<br>TP<br>SGYWKATGADRTVYVEVK<br>RPIGLKKTLVFYVGKAPKG<br>LRSSWIMNEYRLPSGEADR<br>YQKE<br>ISLCKVYKRPGIEDNFHLST<br>TTTRSSSSKAAATMEKKHH<br>RTSASPRLAPMFDGGYSTA<br>HM<br>NKPYSGANTTIAMTSSAAA<br>RAATMAPQTSMFLSTPSLSS<br>TTSTEEDGTSLYHMKVSSIY<br>K<br>GERTRMI

Sspon.06G0013480-1A	*SsNAC091*	ATGGATGAGCAGCAGCAGCAGCA GGCACCATGCGTGCCTCCAGGGT TCAGGTTCCACCCG ACGGAGGAGGAGCTGGTGGGGTA CTACCTGGCCAGGAAGGTGACCT CCCAGAAGATCGAC CTCGACATCATCCGGGAGGTCGA TCTCTACAGGATCGAGCCATGGG ATCTCCAGGAGAGG TGCGGCTACTACGGCGGCGGCGG GGCCGGCGGCCAGGACCAGGAGG AGCCGACGGAGTAC TACTTCTTCAGCTACAAGGACCGC AAGTACCCGAGCGGCACGCGCAC CAACCGCGCCACC GCCGCGGGCTTCTGGAAGGCCAC CGGCAGGGACAAGCCGGTGCTCT CCTCCACCACCACC TCCCGGTCCTCCGCCGGATCCATC AGCGTCGTCATCGGCATGCGGAA GACGCTCGTCTTC TACCGCGGCCGCGCGCCCAACGG CCGCAAGACGGACTGGATCATCC ACGAGTACCGGCTG CAGAGCAACGAGCACGCCCCCAC GCAGGAGGAAGGATGGGTCGTCT GCCGCGCCTTCCAG	MDEQQQQAPCVPPGFRFH PTEEELVGYYLARKVTSQKI DLDIIREVDLYRIEPWDLQE R CGYYGGGGAGGQDQEEPT EYYFFSYKDRKYPSGTRTN RATAAGFWKATGRDKPVL SSTTT SRSSAGSISVVIGMRKTLVF YRGRAPNGRKTDWIIHEYR LQSNEHAPTQEEGWVVCRA FQ KPMPNQQHIRHACYAAAG GGYLPGSYGSTVPATYYYD GPVPAPRLLMGGGAAAAPP PAPH DRGGLAAESKLQVQLLAD MPPLQSPSVDGVVQHHSYN ADHVAAAIAA

Sspon.06G0024580-1B	*SsNAC092*	ATGAGCATCTCGGTGAACGGGCA<br>GTCGTGCGTGCCGCCGGGGTTCC<br>GGTTCCACCCGACG<br>GAGGAGGAGCTGCTCAACTACTA<br>CCTCCGCAAGAAGGTGGCCTCCC<br>AGGAGATCGACCTC<br>GATGTCATCCGCGACGTCGACCTC<br>AACAAGCTCGAGCCATGGGACAT<br>CCAAGAGAAATGC<br>AAGATCGGTTCGGGCCCCCAGAA<br>CGACTGGTACTTCTTCAGCCACAA<br>GGACAAGAAGTAC<br>CCAACGGGGACGCGCACGAACCG<br>CGCCACCGCCGCCGGGTTCTGGA<br>AGGCCACCGGCCGC<br>GACAAGGCCATCTACAACGCCGT<br>CAAGCGCATCGGCATGCGCAAGA<br>CGCTCGTCTTCTAC<br>AAGGGCCGCGCGCCGCACGGCCA<br>GAAGTCCGACTGGATCATGCATG<br>AGTACCGCCTCGAC<br>GACCCTGCTGCTGCTGGCTCCGGC<br>GATGCTGCCGCCGCCGCTGCCGC<br>CACGGTCGCCGCT<br>GCTGCTGCTGCGGCTGCGTCGTCG<br>GACGGTGGGCAGGAGGACGGCTG<br>GGTGGTGTGCAGG	MSISVNGQSCVPPGFRFHPT<br>EEELLNYYLRKKVASQEIDL<br>DVIRDVDLNKLEPWDIQEK<br>C<br>KIGSGPQNDWYFFSHKDKK<br>YPTGTRTNRATAAGFWKAT<br>GRDKAIYNAVKRIGMRKTL<br>VFY<br>KGRAPHGQKSDWIMHEYR<br>LDDPAAAGSGDAAAAAAA<br>TVAAAAAAAASSDGGQED<br>GWVVCR<br>VFKKKHHHKESSGGGGGSK<br>HGSNNEHGHGGGKAAAAA<br>AAAAHQHQHHGGLQYSSS<br>DDTLD<br>QILQYMGRSCKQEHELLSPP<br>PAASAAGPGRAASRYLRPIE<br>TVLGGHGFMKLPPLESPSA<br>A<br>AAALTTPTPHAVSGDAAAA<br>EVVFDDLLGLHRAGGIGITD<br>WAMMDRLVASHLNGQEAP<br>DVA<br>PAADHLGSCFDDATGADDA<br>DAAGLAFYSAAANRLLGSA<br>GSSGAGSDDDLWSFTRSSA

Sspon.06G0028920-1C	*SsNAC093*	ATGACCCACCCTTCCTCGTCGTCG<br>TCCTCCGCCCCGCCGCCACCGCCA<br>CCGCCCGCGGCG<br>GCGGCGGGGGCGGCTGAGGCCAC<br>CTCCCTCGCGCCGGGCTTCCGCTT<br>CCACCCCACCGAC<br>GAGGAGCTCGTCTCCTACTACCTC<br>AAGCGCAAGGTCCTCGGCCGCCC<br>GCTCAAGGTCGAC<br>GCCATCGCCGAGGTCGACCTCTA<br>CAAGCTCGAGCCCTGGGACCTGC<br>CCGCCCGCTCCCGC<br>CTCCGCTCCCGCGACTCCCAGTGG<br>TACTTCTTCAGCCGCCTCGACCGC<br>AAGCACGCCAAC<br>CGCGCCCGCACCAACCGCGCCAC<br>GTCGGGAGGGTACTGGAAGACCA<br>CCGGCAAGGACAGG<br>GAGGTGCGCCATGGGCCCAGGGT<br>CGTCGGGATGAAGAAGACGCTCG<br>TCTTCCACGCCGGA<br>CGCGCCCCCAAGGGCGAGCGCAC<br>CAACTGGGTCATGCACGAGTACC<br>GCCTCGAGGGCGAC<br>GACGCCGCGGGGGTACCGCAGGA<br>CTCGTTTGTGGTGTGCCGGATCTT<br>CCAGAAAGCTGGC	MTHPSSSSSSAPPPPPPPAAA<br>AGAAEATSLAPGFRFHPTDE<br>ELVSYYLKRKVLGRPLKVD<br>AIAEVDLYKLEPWDLPARS<br>RLRSRDSQWYFFSRLDRKH<br>ANRARTNRATSGGYWKTT<br>GKDR<br>EVRHGPRVVGMKKTLVFH<br>AGRAPKGERTNWVMHEYR<br>LEGDDAAGVPQDSFVVCRI<br>FQKAG<br>PGPQNGAQYGAPFVEEEWE<br>EDDADVGLLPVEGDAAVDP<br>EVPRAPVQIPGALERGYLQ<br>MSD<br>LIQGFGDQNGNGTPSLPVSD<br>TSNNSNHSEDVDGNSGDILS<br>DPNLGSNLPQYVEPGEQNT<br>L<br>MLNGNIISNANAGDFFNNSS<br>PSDGFLELKDFADAANLDFP<br>FGNGSTIWPTDGWAWKTP<br>DS<br>AEVVNGANDEIPPLPDDQTF<br>QPDELEQLLQSIQEDSRLGS<br>SIIDPPHSSVTNSVLPEDDS<br>LMFYDAPFDSTMCDDGFG[illegible]

Sspon.07G0001320-2B	*SsNAC094*	CTGCAGTTCCCTCCTGGCTACCGC<br>TTCGTGCCGACCGAGGAGGAGCT<br>CGTCGACGTCTAC<br>CTCCGCGCCAAGATCGAGGGCCA<br>CAAGCTGCCTCTCAACGTCATCAA<br>CGACGTCTCCATC<br>CTCGAGTGGCAGCCCGGCAGACT<br>CGTCGAGGAGTACAAGGGGTATG<br>GCGAGAGCAGGTGG<br>TTCTTCTTCACGGTGCGGGAGCAG<br>TCGTCGTCGAACAAGGAGAAGGA<br>GCCCAGCCGGAAG<br>GTGAGGGTGGACGGCGTCACGGC<br>GACATGGAAGGCCACGGGGAGCG<br>TGCAGCTCATCCGG<br>CGGGCGAGATCCAAGGTGGTCGT<br>GGGCAGCAAGAGGGTGCTCATCT<br>ACAACTCCAGCGAC<br>ACCGCCGAGAACGGCAAGTGGAG<br>CATGCACGAGTACATCCTCAAAG<br>ACCACGCCCAGATT<br>GGGCAATATGCCCTGTACTCGATC<br>CAAAGGAAGCAGCACTCGGACAC<br>CGAGGGCAATGCC<br>GGCAATGACGACATGGATCCAGA<br>GAAGAAGAAGAAGAAGGAGACT<br>AGAAAGAGGAAGAGG	LQFPPGYRFVPTEEELVDVY<br>LRAKIEGHKLPLNVINDVSI<br>LEWQPGRLVEEYKGYGESR<br>W<br>FFFTVREQSSSNKEKEPSRK<br>VRVDGVTATWKATGSVQLI<br>RRARSKVVVGSKRVLIYNS<br>SD<br>TAENGKWSMHEYILKDHA<br>QIGQYALYSIQRKQHSDTEG<br>NAGNDDMDPEKKKKKETR<br>KRKR<br>TETETETPTELEGVQLPHPG<br>TTTLLAEPPLAKAQRKKKPS<br>MQVGVEGEQQQQAQSPME<br>PV<br>TPPKKEELAPRQQEGRQQPP<br>PPAVVLSAVPLQAVRVCPPS<br>EPDGTLGASAPKPEEDTPDD<br>MAQLLALLGYPAMFCNNQ<br>GQEQWPPSLTGTTTLLAAP<br>DSNNIVDHAPFQPFQQEEDS<br>FAM<br>QNQHLSLGENQSVYMEQW<br>WQHADLFLPNNTTQPMNSS<br>GGWEHGNDQRQIHQQQEE<br>NNGGG

Sspon.07G0007590-1A	*SsNAC095*	ATGCCCGCCAGTGTCCCGGTCCA<br>ACTCCAGTCCCCCGCGCCTTTAAA<br>AGAGCCTTTCCCC<br>AGCCACGCGATCCCTCACCCCCCA<br>CACAAACCGATCTCGCATCCATTG<br>ACCAGACGGCGG<br>CGCTCATCTCCTCCCCCACGGCTC<br>TCCCCATCGGCCTGCTGGCTTCCC<br>TTCGTCTTCCCC<br>GATACTGATCCTCATCACCCATGT<br>CTGCTGAAGTCGCCGGAGGACAC<br>CCTTATTTCCTCT<br>CGTGCTACTTCTGGTCTTTCTATT<br>GGTGTTGAAGGCAAGAGAAGCTT<br>TAGGGTGATGGCG<br>CAAACTAGCCTGCCTCCTGGTTTT<br>CGTTTCCACCCAACTGATGTTGAG<br>CTTGTTTCCTAC<br>TACTTGAAGAGGAAGATCATGGG<br>AAAGAAACTTATCGTCGATGCTA<br>TATCAGTAGTTGAC<br>TTGTACAAGTTTCCTCCCTGGGAT<br>CTACCTGACAAATCCTCCCTTAGA<br>AGCAAAGATCTT<br>GAATGGTTCTTCTTTTGTCCTCGT<br>GACAAGAAGTATCCTAATGGGTC<br>TAGGACAAACCGT	MPASVPVQLQSPAPLKEPFP<br>SHAIPHPPHKPISHPLTRRRR<br>SSPPPRLSPSACWLPFVFP<br>DTDPHHPCLLKSPEDTLISSR<br>ATSGLSIGVEGKRSFRVMA<br>QTSLPPGFRFHPTDVELVSY<br>YLKRKIMGKKLIVDAISVVD<br>LYKFPPWDLPDKSSLRSKD<br>LEWFFFCPRDKKYPNGSRT<br>NR<br>ATPNGYWKTSGKDRIITLNS<br>RTVGMKKTLIFHEGKAPKG<br>DRTDWVMYEYKMEDEDLV<br>SAG<br>FSKDAYVLCKIFKKSGLGPR<br>IGEQYGAPFNEAEWDNAEA<br>ESSMFPLMTSSEVVNPTEGP<br>R<br>AQPAAPAGALQEPPLHNSS<br>ATCAGEESSFDHATANTCA<br>EDVTFGYTVASSAIQDIPAQ<br>MS<br>GDGVVSVNNISNEANDMYS<br>PRDCDGFLLEELSRFLNDSP<br>VRNTPFGECSGLPPMPEAEA<br>H<br>AFEVNSFGLYNELSGLVGF

Sspon.07G0011330-1A	*SsNAC096*	ATGAGCAGCAGCAACAGCACCTC<br>CGACCTCAGCGGCGAGAAGCTTA<br>AGGACCAACGACGC<br>TCGACACCAGCTTCACGGCGCTG<br>CCCTAGCTGCGACCATGAGCTCG<br>ACTGGAATGACATG<br>GTTGGGTTTCCAGCCGGTGTCAAG<br>TTTGATCCGTCAGACCAGGAGCTC<br>ATAGAGCACCTG<br>GAGTCCATGGTGAAGGAAGGAGG<br>CTCCGGAGCACACCCCCTCATTGG<br>TGATTTTATACCC<br>ACACTTGAAGGAAACGATGGCAT<br>TTGCTACACCCATCCTGAGTATCT<br>CCCAGGTGTGACA<br>AGAGATGGCGTCAGCAAGCACTT<br>CTTCCACAGGCCATCCAAAGCCT<br>ACACCTCTGGCACA<br>AGGAAGAGGAGAAAGATCCAGTC<br>AAAACGCGGTGACGATGTTGGCG<br>AGGCGCGGTGGCAC<br>AAGACTGGCAAGACACGTCCAGT<br>AATCGTTGGTGGTCGGCAGAAGG<br>GGTGCAAGAAGATC<br>CTGGTGTTGTACAGTAACTATGG<br>AAAGCAAGGGAACAAGCCGGAG<br>AAGACCAACTGGGTG	MSSSNSTSDLSGEKLKDQR<br>RSTPASRRCPSCDHELDWN<br>DMVGFPAGVKFDPSDQELI<br>EHL<br>ESMVKEGGSGAHPLIGDFIP<br>TLEGNDGICYTHPEYLPGVT<br>RDGVSKHFFHRPSKAYTSG<br>T<br>RKRRKIQSKRGDDVGEARW<br>HKTGKTRPVIVGGRQKGCK<br>KILVLYSNYGKQGNKPEKT<br>NWV<br>MHQYHLGRDEEKDGELVV<br>SKVFYQQTQLRSTATMMEQ<br>NDDEKVEVTSEAMKDMLP<br>GCGAE<br>ATETADAMKQRQQHQQQR<br>QADGHSRFTSAKMSRELKF<br>YMENNQQVGVGDPLAGSA<br>EQKKV<br>HCRRPCSSQEAMIACQTTST<br>EEVRMCRKLQLSTRRYNGL<br>MNTGLLITTKMDSTSSYTIK<br>S<br>S

Sspon.07G0017230-1A	*SsNAC097*	ATGTGTAATATCTCACTCTCATCC<br>TGCCGGAGTCGAAGAGGGAGGTG<br>TCGACAGGAAAGG<br>GATGCTATGGGTGGAGAGGGAGG<br>AGCCGCCGGCGGCCTAGTCGTCA<br>CGAGCCAAGCGGCA<br>GGGCCGAAGGAGTCGCAGTGCCG<br>GTTCAGATGGTTCTCCCCCGACCC<br>TGAGCGTAGCGTT<br>GATGTGAAAAAAAGGCACCCGAG<br>AGTTGAACCGTGGCCATCAGGGG<br>AGAGGGAGGAGTCA<br>CGCCATATCCCGCAGTCCCCTTCT<br>CGTTCCCTCGTCGCAGGGATGAC<br>GCCGCCGGCCCTA<br>GGCCAGGAGATCTACAGCCGCGG<br>CTTCCGCTTCAGCCCGACTCCGCA<br>GGAGGCGGTCACG<br>TACTACCTGCCCCGCCTCATCTCC<br>GGCGAGCCGCTGCACGAGGTTGT<br>CCGCTCCGTCATC<br>CACCACGCCGACGTCTACGCGTG<br>CGAGCCCGCCGAGCTCGCCCGCC<br>GGTTCCGGCCCATG<br>CCGAGGACCGACGACCGCTTCTT<br>CTTCGCGCCCTGCAAGAACAAGG<br>CCGTGCGCGCCGCC	MCNISLSSCRSRRGRCRQER<br>DAMGGEGGAAGGLVVTSQ<br>AAGPKESQCRFRWFSPDPE<br>RSV<br>DVKKRHPRVEPWPSGEREE<br>SRHIPQSPSRSLVAGMTPPA<br>LGQEIYSRGFRFSPTPQEAV<br>T<br>YYLPRLISGEPLHEVVRSVI<br>HHADVYACEPAELARRFRP<br>MPRTDDRFFFAPCKNKAVR<br>AA<br>SCPSSWAAQTTAEIKENDV<br>RIGELRKLRYKKRGVLTDW<br>LMDEYSSCDDNDKQQQFVF<br>CKV<br>YVSPRAAPSSAARHESAAFF<br>ALPPATPAAVIAQAAAMRT<br>APPLPQDAMPPCAKRTAPL<br>LQ<br>QAAMPPCAKRTAPPPHPAM<br>PPCAKRMRGPLQLAPPAPT<br>RTPVTAVRKLYFARIAPPQP<br>CA<br>PIRDTAISGSNSGSGAASPDP<br>FCGGAASLANASGFCCAAS<br>GAASSDQIIAACCSEVVLRT

Sspon.07G0019480-2C	*SsNAC098*	ATGGCGGCGCCATTCCGGGCGCT<br>TCCGCCGGGCGTCTACTTCAACCC<br>GACGGCGGAGGAG<br>TGCGTGCGCGACTACATCAAGCC<br>GTGGGCCGCGGGCGTGCCGCCGC<br>CGACGGACCGGGTC<br>ATCTGCGACGTGGACGTCTACAG<br>CGACAGCCCCGGCGCGCTGCTGC<br>TGGGGCGCGAGCCG<br>GGCTTCTCGCGGGGGTTCGACCA<br>CAAGTGGCTCATGCTCAGCCATTG<br>CGGCGGCCGCGGC<br>AAAACGGCGGCGGGCCGCGGGAA<br>GCGCGTAGTCGCCACGGGCGGAA<br>GCTGGCAGTCGGAG<br>CAGACGCCCAAGGGCGTCGTCGG<br>TGCCTCGGACGGCGAGGACGAGG<br>ACGAGCAGCCCCAC<br>GGCGGCAGGAGGCGCTCCTTCGG<br>CTTCTACGTCGGCAAAAATGGGA<br>GGAAGAGGGGAGAG<br>AAGACGCCGTGGATCATGGAGGA<br>GTTCACCGCCTTGGAGGACGGAC<br>ACGGCGGAGGCGAC<br>GGGACCTTCGTGGTGTTCTGCAGG<br>ATCTACCTCACGCCGCGCCTGGA<br>AAAGGAGGACAAG	MAAPFRALPPGVYFNPTAE<br>ECVRDYIKPWAAGVPPPTD<br>RVICDVDVYSDSPGALLLG<br>REP<br>GFSRGFDHKWLMLSHCGG<br>RGKTAAGRGKRVVATGGS<br>WQSEQTPKGVVGASDGED<br>EDEQPH<br>GGRRRSFGFYVGKNGRKRG<br>EKTPWIMEEFTALEDGHGG<br>GDGTFVVFCRIYLTPRLEKE<br>DK<br>EKKRQILGDDMVAFDRNGK<br>LKPVRVVVSPELFDAAAQG<br>QVPAPPRVLGFQQAQPARR<br>VLG<br>HQQGESPTPSPSPRFLGYQQ<br>GQPAAPPLLRFLGHQHGQA<br>AATPAVLGDHHGRAMLPW<br>GVP<br>AHHHGHAAAAPHQRFLGY<br>RQGQAAVQGGPDEYCGTA<br>VQPQLLHVLDPCYNQEASP<br>SVRLV<br>DPQQSKVMTHTEKKPRLAY<br>GSPPPPPQSSESGPSSCVVHI<br>SPSQEQGVVIQIHDDNQCSA

Sspon.07G0020380-1A	*SsNAC099*	ATGGAGCGGTTCGGCGTGCTGGG<br>CACGCGGCTGGGCCTGGACGGCG<br>TCGTCGGCGGCGGC<br>GGAGGCGAGCTGCCGCCGGGGTT<br>CCGGTTCCACCCGACGGACGAGG<br>AGCTCATCACCTAC<br>TACCTCCTCCGCAAGGCCGTGGA<br>CGGCAGCTTCTGCGGCCGCGCCA<br>TCGCCGAGATCGAC<br>CTCAACAAGTGCGAGCCATGGGA<br>GCTCCCGGACAAGGCGAAGATGG<br>GGGAGAGGGAGTGG<br>TACTTCTACAGCCTCCGCGACCGC<br>AAGTACCCGACGGGCCTGCGCAC<br>CAACCGCGCCACG<br>CTGGCCGGCTACTGGAAGGCCAC<br>CGGCAAGGACCGCGAGATCCGCA<br>GCGCCCGCTCCGGC<br>GCGCTGGTGGGCATGAAGAAGAC<br>GCTCGTCTTCTACCGCGGCCGCGC<br>CCCGAAGGGACAG<br>AAGACGCACTGGGTCATGCACGA<br>GTACCGCCTCGAGGGCACCTACG<br>CCTACCATTTTCTC<br>CACAGCTCCACAAGGGATGAGTG<br>GGTGATCGCCAGGGTGTTCCAGA<br>AGCCCGGCGAGGTC	MERFGVLGTRLGLDGVVG<br>GGGGELPPGFRFHPTDEELI<br>TYYLLRKAVDGSFCGRAIA<br>EID<br>LNKCEPWELPDKAKMGER<br>EWYFYSLRDRKYPTGLRTN<br>RATLAGYWKATGKDREIRS<br>ARSG<br>ALVGMKKTLVFYRGRAPK<br>GQKTHWVMHEYRLEGTYA<br>YHFLHSSTRDEWVIARVFQ<br>KPGEV<br>PPARKHHRLGGLSSAGGGE<br>SCFSDSTSASIGGGGGGASA<br>SSAPRPLPLTVTDASSLSLFA<br>SAAAANAADGDSSSYCGGA<br>ANNANNGNNLVTGRELVPC<br>FSTSTTTGAGGLDAAALGIG<br>QP<br>YNAAVPLPLAFEPPPPTPAF<br>FPNLRSSLQLQVQQDNNLE<br>LPLFLSAAGGLSAATLGMG<br>SM<br>GGGALHHWPLAGMEVKVE<br>GRSAPPQMAVGPGQLDGAF<br>GWGY

Sspon.07G0024330-1B	*SsNAC100*	ATGCCTCCTCCGCCGGGCCATTGC<br>TCTTTCACCCTGTCGGAGCTCCTC<br>TCCTTCCTCCGT<br>CTTAAGATCGCCGGCGAGCCCCT<br>CCCTGCCACCGCCGCGGCACATTT<br>CCACGACGCCGAC<br>ATCTACACCGCCGACCCGGCCCT<br>GCTCGCGACGCTCTTCGATCCGGC<br>TCCGGAGAAGAAA<br>GGGGAGAGCGGCTCGTGGTTCTT<br>CTTCACCCACGTGAGGCCCAAGA<br>GCAGCACCGACAGC<br>CGCAAGTCGCGACAGGTTGCCGG<br>TGGAGTGGGCACGTGGCACTCCG<br>AGCGCGCCCCCCGC<br>CGCGTGCTCGACGACGAGGGCAA<br>TTGCGCTGGACACAGCCAGTATTT<br>CTCCTACAAGCTC<br>AAGATCGGGAAGAACTGCAGCGA<br>GAGGACCGAGTGGTACATGTTGG<br>AGTTCAGCGACGGC<br>CAAGAAGCCGATCACGAGCGCGT<br>CCACGGCGGCGAGCCCCAGCTGG<br>TCCTTTGCAACATC<br>TACAAGGCGCATACCCATTCGCG<br>CAGCAGCAACGGATCCACGTCGA<br>CACCGTCATACTCT	MPPPPGHCSFTLSELLSFLRL<br>KIAGEPLPATAAAHFHDADI<br>YTADPALLATLFDPAPEKK<br>GESGSWFFFTHVRPKSSTDS<br>RKSRQVAGGVGTWHSERA<br>PRRVLDDEGNCAGHSQYFS<br>YKL<br>KIGKNCSERTEWYMLEFSD<br>GQEADHERVHGGEPQLVLC<br>NIYKAHTHSRSSNGSTSTPS<br>YS<br>ASARKRKAVGEASAPHVK<br>AKRQLFDSSAPASAARSQE<br>QVRSTTPSNLRKIGDTSDCI<br>ALM<br>TKAGGEATTSQLKIGDTSVF<br>SRFWPEPEKSASDCIALMSK<br>ADGEATTSQLKIGDTSDFSR<br>FWPEPEKSFDLGYTTTCGA<br>LPTLENCSRTNVRDVGDVF<br>CGQDAWPSAFHSSNDTTTF<br>VCG<br>ETNPLSWAMRMRAALYVA<br>QTLECCSSKGRALNHDLHA<br>YRVVFDVDGNPRLSCFGLM<br>KNSR<br>DGKSYSTNLAFMPPEYLKT

Sspon.07G0034490-1C	*SsNAC101*	CCGGCCGACCACGACGCGGACAC<br>AGCCCACCATATAAGCTGCCAAA<br>AGCACATTGGCTCC<br>GGCGTAACGACTCCGCCACGATT<br>ACGACTCCACGCCGTCGAAATTG<br>CTCTGCTCTCCGTC<br>TACAACACCGCCGGGGCCGGGGG<br>CCGGAAGCCGGCGAGCGCGATGG<br>CGGCGCCATTCCGG<br>GCGCTTCCGCCGGGCGTCTACTTC<br>AACCCGACGGCGGAGGAGTGCGT<br>GCGCGACTACATC<br>AAGCCGTGGGCCGCGGGCGTGCC<br>GCCGCCGACGGACCGGGTCATCT<br>GCGACGTGGACGTC<br>TACAGCGACAGCCCCGGCGCGCT<br>GCTGCTGGGGCGCGAGCCGGGCT<br>TCTCGCGGGGGTTC<br>GACCACAAGTGGCTCATGCTCAG<br>CCATTGCGGCGGCCGCGGCAAAA<br>CGGCGGCGGGCCGC<br>GGGAAGCGCGTAGTCGCCACGGG<br>CGGAAGCTGGCAGTCGGAGCAGA<br>CGCCCAAGGGCGTC<br>GTCGGTGCCTCGGACGGCGAGGA<br>CGAGGACGAGCAGCCCCACGGCG<br>GCAGGAGGCGCTCC	PADHDADTAHHISCQKHIGS<br>GVTTPPRLRLHAVEIALLSV<br>YNTAGAGGRKPASAMAAP<br>FR<br>ALPPGVYFNPTAEECVRDYI<br>KPWAAGVPPPTDRVICDVD<br>VYSDSPGALLLGREPGFSRG<br>F<br>DHKWLMLSHCGGRGKTAA<br>GRGKRVVATGGSWQSEQT<br>PKGVVGASDGEDEDEQPHG<br>GRRRS<br>FGFYVGKNGRKRGEKTPWI<br>MEEFTALEDGHGGGDGTFV<br>VFCRIYLTPRLEKEDKEKKR<br>QI<br>LGDDMVAFDRNGKLKPVR<br>VVVSPELFDAAAQGQVPAP<br>PRVLGFQQAQPARRVLGHQ<br>QGES<br>PTPSPSPRFLGYQQGQPAAP<br>PLLRFLGHQHGQAAATPAV<br>LGDHHGRAMLPWGVPAHH<br>HGH<br>AAAAPHQRFLGYRQGQAA<br>VQGGPDEYCGTAVQPQLLH<br>VLDPCYNOEASPSVRLVDP

Sspon.07G0034910-1C	*SsNAC102*	ATGTCGGGCACCCAGCAGCTGCA<br>GCGCACCGCCTCCGATCCGGCTCC<br>GGCTCTCTTCCAG<br>CAGCCTTCTCTCACCGTCACCGCA<br>CAACACAATCCCACGGAGATGGC<br>GCCACCGGCCCTA<br>GGCCCGGAGATCTACGGCCTTGG<br>CTTCCGCTTCAACCCGACGCCGCA<br>GGAGGCGGTCACC<br>TACTACCTGCCCCGCCTCGTCTCC<br>GGCGACCCGCGGGCGCTGCACGA<br>GGCCGTTCGCCCC<br>GTCATCCACCACGCCGAGGTCTA<br>CGCCTGCGACCCCGCCGACCTCGC<br>CCGCCGGTTCCGC<br>CCCATGCCGAGGACCCACAACCG<br>CTTCTTCTTCACCGTCGTCAAGAA<br>GGGCGTGCGCGCC<br>GCCGGCCCCGGCTCCTGGGCCGG<br>GCAGAGCACGGCGGAGATCAAGG<br>ACAAGCAGGGTGTC<br>CAGATCGGCGAGCTCAGGAAGTT<br>CCGGTACAAGAAGCGTGGCGTCT<br>TGACGGACTGGCTC<br>ATGGAGGAGTACTCGTCCTGCGG<br>TCTGGACGGGGCCGGCGATGGCG<br>GCATGCAGTTTGCG	MSGTQQLQRTASDPAPALF<br>QQPSLTVTAQHNPTEMAPP<br>ALGPEIYGLGFRFNPTPQEA<br>VT<br>YYLPRLVSGDPRALHEAVR<br>PVIHHAEVYACDPADLARR<br>FRPMPRTHNRFFFTVVKKG<br>VRA<br>AGPGSWAGQSTAEIKDKQG<br>VQIGELRKFRYKKRGVLTD<br>WLMEEYSSCGLDGAGDGG<br>MQFA<br>LCKVYVSPKAAPNSDAYKE<br>SAACFATPPPATPAVITQPA<br>AAKRPAPPQDVMPPCAKR<br>MRV<br>PAVQPAPPPPQPPAPPAPTLS<br>ALAAVAKMHFAPPQPSAPR<br>RGSTPQPSPALAPPPAPPAP<br>TRSHLAAIRRMSFAPPQPCV<br>PRRGLPADSVTLPSPAPIQAP<br>ALPVPIRPAVEARRLPLPM<br>PAPPRPLSQPKEQVPLPTPQ<br>VMRVSLPKQRRILDPFEAS<br>MLRDEAEEQTVAAAPHPPE<br>SP<br>AAAPVLQDDDDDDLVKAL

Sspon.07G0036500-1D	*SsNAC103*	ATGGCGGACCAGCAGCAACCGCA<br>GCCAAAGGAGATGAACATTCTCC<br>ATGCCAGTGGCCTC<br>GACCTGCATCTAGGGTTCCGCTTC<br>CATCCAAGTGACTATGAGATTGTC<br>AATGACTACTTC<br>ACGAACAAGGTGCACAACAGGGA<br>CTTCATTTGTGTCCTCGGGGAGGT<br>CGACCTAAATAAG<br>ACTGAGCCATGGGACCTCCCGAG<br>GGAGGCAAAAATAACCGAGAAAG<br>AGTGGTACTTCTTC<br>TCACAGAAGGACCGCAAGTACCC<br>GACGGGGCTAAGGGTGAATAGGG<br>TCACAAAAGCTGGT<br>TATTGGAAGGCGACCGGCAAGGA<br>CAAGGAGGTATACAAGCCCACCA<br>AAGGGGAAGGGGTG<br>GTGCTGCTCATCGGCATGAAGAA<br>GACGCTTGTCTTCTACGAAGGCA<br>GGGCTCCCAGGGGC<br>AACAAAACGAATTGGGTGATGCA<br>TGAGTATAGGCTCGAAGGTAGCG<br>GCAGGCTCCCTGGC<br>CTCGCATCCGCATCCAGCTCAGCC<br>ACTAACGCCACCATGGCTATAGA<br>AGCTTCAGCTTCT	MADQQQPQPKEMNILHASG<br>LDLHLGFRFHPSDYEIVNDY<br>FTNKVHNRDFICVLGEVDL<br>NK<br>TEPWDLPREAKITEKEWYF<br>FSQKDRKYPTGLRVNRVTK<br>AGYWKATGKDKEVYKPTK<br>GEGV<br>VLLIGMKKTLVFYEGRAPR<br>GNKTNWVMHEYRLEGSGR<br>LPGLASASSSATNATMAIEA<br>SAS<br>ASKGCGSGNNTGMMYDEW<br>VVCHVFHKTTKIRKTTVPA<br>YQVAMLDAEIDQNQNNIPA<br>MPNP<br>MPLQLPQPVPMPMQFPVLP<br>DFAMDPVPPYYPNPNASVG<br>MPPMAGIGGADGLQINGAL<br>FGN<br>PMVVPPPMNFYHQMGMGE<br>PVG

Sspon.08G0000770-1A	*SsNAC104*	ATGGCGGAGCAGCAGCGGCGTGG<br>CGCGTCACCGCCGTCATCCGGCG<br>GCGCGGGGGCGGCG<br>GAGCTGGAGCTGCCGGGGTTCCG<br>GTTCCACCCGACGGAGGAGGAGC<br>TGCTGGAGTTCTAC<br>CTGACGCAGGTAGCGCACGGGCG<br>TAAGCTCAAGTTCGACATCATCCC<br>CACGGTGCACCTG<br>TACCGGCACGACCCCTGGGAGCT<br>CCCGGGCCTGGCGCGCATCGGCG<br>AGCGGGAGTGGTAC<br>TTCTTCGTGCCCCGCGACGGCGGC<br>GGCCGGGCGGCCAAGCAGGCCGG<br>TTCCGGTTCCGGC<br>TCCGGCTCCGGCGGCGGTCGGCC<br>TAGCCGCACGACGGAGCGCGGGT<br>TCTGGAAGGCCACG<br>GGGTCCGACCGCGCCGTGCGGTG<br>CGCCGCTGACCCCAAGCGCCTCA<br>TCGGGCTCAAGAAG<br>ACGCTCGTCTACTACGAGGGGCG<br>CGCGCCACGTGGCACCAAGACCG<br>ATTGGGTCATGAAC<br>GAGTATCGCCTCCCCGACGCCAC<br>CACCTGCTGCAATGCCGCCGGCG<br>ACTCATCGGCGGCC	MAEQQRRGASPPSSGGAGA<br>AELELPGFRFHPTEEELLEF<br>YLTQVAHGRKLKFDIIPTVH<br>L<br>YRHDPWELPGLARIGEREW<br>YFFVPRDGGGRAAKQAGSG<br>SGSGSGGGRPSRTTERGFW<br>KAT<br>GSDRAVRCAADPKRLIGLK<br>KTLVYYEGRAPRGTKTDW<br>VMNEYRLPDATTCCNAAG<br>DSSAA<br>ASTNKLPKILRTVNMY

Sspon.08G0003810-1A	*SsNAC105*	ATGGCCATCAACTCATCGCTGAG<br>CATGGTGGAGGCCAGGCTGCCTC<br>CGGGGTTCAGGTTC<br>CACCCGCGGGACGACGAGCTCGT<br>GCTCGACTACCTGGCCAAGAAGC<br>TTGGCAGCGCCAGC<br>GGCGGCGGAGGCCCGGTGGTGGT<br>GAGCATCTACGGTTGCCCCACCAT<br>GGTCGACGTCGAT<br>CTCAACAAGTGCGAGCCGTGGGA<br>TCTTCCTGATATCGCGTGCATTGG<br>TGGAAAAGAGTGG<br>TATTTCTACAGCCTTAGGGATAGA<br>AAGTATGCCACCGGCCAACGTAC<br>AAACAGAGCAACT<br>GATTCGGGATATTGGAAGGCTAC<br>GGGGAAAGACCGTCCGATAAGCA<br>GGAAAGGGTTACTT<br>GTTGGTATGCGCAAAACTCTTGTG<br>TTTTATCAAGGTAGAGCCCCAAA<br>GGGAAAGAAGACT<br>GAGTGGGTTATGCATGAATTTCG<br>CATGGAAGCGCAAGGTGATCCCA<br>TGAAATTACCTTTC<br>AAGGAGGACTGGGTCTTGTGTAG<br>AGTTTTCTACAAGACTAGGGCGA<br>CAGTTGCAAAGCCA	MAINSSLSMVEARLPPGFRF<br>HPRDDELVLDYLAKKLGSA<br>SGGGGPVVVSIYGCPTMVD<br>VD<br>LNKCEPWDLPDIACIGGKE<br>WYFYSLRDRKYATGQRTN<br>RATDSGYWKATGKDRPISR<br>KGLL<br>VGMRKTLVFYQGRAPKGK<br>KTEWVMHEFRMEAQGDPM<br>KLPFKEDWVLCRVFYKTRA<br>TVAKP<br>PTESSSSFNIDAATTSLPPLIN<br>NYNISFDQPGSSVQNLEGYE<br>QVPCFSSNPSQPSPSMNA<br>PLTSAAMADPEQHMGKSIIK<br>DVLMSQFSRFEGSVKREAP<br>QSNFSQDGFEYLAESGFTQ<br>MW<br>NSFN

Sspon.08G0007270-2C	*SsNAC106*	ATGGATGGTGCAGCGGCGGCGGC<br>AACAGGCGGAAGCAGTGGCGGCC<br>ACCGCCACATGGAC<br>TCAAGGATCGAGGAGCATGGGAA<br>GTACCTGTCGGAGTCGAGCTGCT<br>GCCCACAGTGCGGC<br>CACAAGACCGACCGCAAGCTGGA<br>CTGGGTGGGGCTGCCGGCGGGGG<br>TGAAGTTCGACCCG<br>ACGGATCAGGAGCTGATTGAGCA<br>CCTGCAGGCCAAGCACAGTTCCA<br>TTCCATGGGTTATT<br>GATCTCAGTATGTTTGACAAAGG<br>ACGGCCGGAGCAGGCACTTCTTC<br>CACCGGCCATCCAA<br>GGCTTACACCACCGGCACTCGCA<br>AGCGCCGCAAGATTCATCAGCCG<br>CCGGCCGCCGAGGG<br>CTCATCCTCCTCCGCCGTGTCACC<br>GCGCAGCAGCAGCAGCAGCAGCG<br>GAGCGAGACGCGG<br>TGGCACAAGACCGGCAAGACGCG<br>GCCGGTGGTGGTCGCCGGCCAGC<br>AGCGTGGGTGCAAG<br>AAAATCCTGGTGCTCTACACCAA<br>CTTCGGCAAGAACCGCAAGCCGG<br>ATAAGACCAACTGG	MDGAAAAATGGSSGGHRH<br>MDSRIEEHGKYLSESSCCPQ<br>CGHKTDRKLDWVGLPAGV<br>KFDP<br>TDQELIEHLQAKHSSIPWVI<br>DLSMFDKGRPEQALLPPAIQ<br>GLHHRHSQAPQDSSAAGRR<br>G<br>LILLRRVTAQQQQQQRSETR<br>WHKTGKTRPVVVAGQQRG<br>CKKILVLYTNFGKNRKPDK<br>TNW<br>VMHQYHLGDKEEEREGEL<br>VVSKIFYQTQPRQCGGAAE<br>PATAAASSDTVEEAAADPP<br>VPEG<br>MVAPPPDVVAGAFHGAAGI<br>DEFNFAQFRSSFEEVDVGAS<br>VQVSMRDDEEVHAGHPNL<br>HQE<br>HNLHQHQFGNQEQRQRMA<br>AAAAAFHINKPAEPITTMIT<br>SSPVVHHGSAVLQHPDAYG<br>HGT<br>TYRHHQQVEDELLHQQPQN<br>FDGRSTAGLEAVIMGCTSR<br>RSKRGSGSGGNKESTGWPY

Sspon.08G0010220-1A	*SsNAC107*	ATGACGACAACTTGGATAATTGA<br>CAGCCAAAGATTTGCCACCAAAA<br>TAAAAAATGCTTCT<br>GGGTCTTCAGATCCCAGTAAACA<br>GAAATGGATCAGCAACCCAAGTA<br>AAGAGTGCCCAAAG<br>TGCAGCCATGTCATCGATAACAG<br>TGATGTTGTTCACCAGTGGCCCGG<br>TTTGCCTAAAGGT<br>GTAAAATTTGATCCGTCTGACCAG<br>GAATTAATCTGGCACTTACGGGC<br>AAAACATGGAAAT<br>TCTGGTATAAAACCTCATCCGTTC<br>ATTGACGAATTTATTCCAACAGTT<br>GAGGAAGATGAA<br>GGCATCTGCTACACTCATCCACAA<br>AAACTTCCAGGTGTTAAGCAAAA<br>TGGAAGTGTATCA<br>CATTTCTTCCACAGAACATTTAAG<br>GCCTACAACACTGGGACTAGGAA<br>GCGTCGAAAGATA<br>AACACTGATGATGCTGATGTCCGT<br>TGGCACAAGACAGGCAAGACAAA<br>GCCAGTATTAGTT<br>GATGGAAAACAGCTTGGCTGTAA<br>GAAAATAATGGTGCTTTATATGA<br>GCCTGGCAAAGGGT	MTTTWIIDSQRFATKIKNAS<br>GSSDPSKQKWISNPSKECPK<br>CSHVIDNSDVVHQWPGLPK<br>G<br>VKFDPSDQELIWHLRAKHG<br>NSGIKPHPFIDEFIPTVEEDE<br>GICYTHPQKLPGVKQNGSV<br>S<br>HFFHRTFKAYNTGTRKRRK<br>INTDDADVRWHKTGKTKP<br>VLVDGKQLGCKKIMVLYM<br>SLAKG<br>GKAEKTNWVMHQYHLGTG<br>EDERDGEYVVSKLFFQQQF<br>KPGDKNAQELTTSDDLESM<br>AAEA<br>DLPDFTTLPADRHVGTIQVA<br>HNSEHNLCQATTSDDLESM<br>AAEADLPDFTTLPADKHVG<br>TI<br>QVVHNSEQNLYQVNRNCEI<br>NIEETVVLPPSEKTEDGDNP<br>QSQDPKLWEGDSQFELLDS<br>QQ<br>LAEGLALCDEFLLSQSQTSC<br>GGGDEPRVTKPCLAAYAHL<br>SAEDFKKDLEECQQLEPTD

Sspon.08G0010230-4D	*SsNAC108*	ATGGATCCCAAATTCAAGGGAGA<br>GTGGAGTGCCTCTGAAATCGAGA<br>TGGTGAAATCTCTC<br>ATTGCAAAGGACAACGCCAACAA<br>CAATGGTGCTAGTGATATGAACA<br>AGAAGCACAATCAA<br>ATTGTGGATGAGCTCCAGGCAAT<br>GTTCCCTAGCAAGGAGAAGCATC<br>AGAGTGGCAACCAA<br>CATGTGGAAGCAAGTAGCAACCT<br>CATGAATCAACCCTTTGGGGTGTT<br>TGTGGGGGATCCA<br>TCCATGGGCAACAAGGAAGCATT<br>TAATGTTATCAACAAGCAATTCCT<br>CCGTGGTCTACAT<br>GTGTACGGTCGTGGAAATTGGAA<br>GAACATCTCCAGGCACTTTGTTAC<br>CACCAAGACCCCA<br>GTGCAAGTTTCCAGCCATGCTCAA<br>AAGTACTTCCTTAGGAAAGAAAA<br>TAGCACCAAGAAG<br>CAGCGTTATAGCATCAATGACATT<br>GAACTTCATGATTTTGAGCCCTTG<br>TCGCAGACAAAT<br>GCTTCTGCCTGGGAGGGGCCCAC<br>CTTTGGTGGAGGTGTCTACAAAA<br>CAAATCACTATAGC	MDPKFKGEWSASEIEMVKS<br>LIAKDNANNNGASDMNKK<br>HNQIVDELQAMFPSKEKHQ<br>SGNQ<br>HVEASSNLMNQPFGVFVGD<br>PSMGNKEAFNVINKQFLRG<br>LHVYGRGNWKNISRHFVTT<br>KTP<br>VQVSSHAQKYFLRKENSTK<br>KQRYSINDIELHDFEPLSQT<br>NASAWEGPTFGGGVYKTN<br>HYS<br>FGGHPTSMNNAQAWSPFLY<br>HTSHGSSSNSQMVTLAIGQ<br>QQEQMGASSSLVAPTMEAD<br>GGH<br>LDWTSDKTTWIIDSQRFAT<br>KIKNASGSSDPSKQKWISNP<br>SKECPKCSHVIDNSDVVHQ<br>WP<br>GLPKGVKFDPSDQELIWHL<br>RAKHGNSGIKPHPFIDEFIPT<br>VEEDEGICYTHPQKLPGVK<br>Q<br>NGSVSHFFHRTFKAYNTGT<br>RKRRKINTDDADVRWHKT<br>GKTKPVLVDGKQLGCKKIM

Sspon.08G0010260-1A	*SsNAC109*	ATGACAAGGACAACTTGGATAAT<br>TGACAGCCAAAGATTTGCCACCA<br>AAATAAAAAATGCT<br>TCTGGGTCTGCAGATCCCAGTAA<br>ACAGAAATGGATCAGCAACCCAA<br>GTAAAGAGTGCCCA<br>AAGTTGTTCACCAGTGGCCCGGTT<br>TGCCTAAAGGCTAAAGGTGTAAA<br>ATTTGATCCGTCT<br>GACCAGGAATTAATCTGGCACTT<br>ACGGGCAAAACATGGAAAATCTG<br>GTATAAAACCTCAT<br>CCGTCCATTGACGAATTTATTCCA<br>ACAGTTGAGGAAGATGAAGGCAT<br>CTGCTACACTCAT<br>CCACAGAAACTTCCAGGTGTTAA<br>GCAAAATGGAAGTGTATCACATT<br>TCTTCCACAGAACA<br>TTTAAGGCCTACAACACTGGGAC<br>TAGGAAGCGTCGAAAGATAAACA<br>CTGATGATGCTGAT<br>GTCCGTTGGCACAAGACAGGCAA<br>GACAAAGCCAGTATTAGTTGATG<br>GAAAACAGCTTGGC<br>TGTAAGAAAATAATGGTGCTTTA<br>TATGAGCCTGGCAAAGGGTGGGA<br>AGGTTGAGAAGACC	MTRTTWIIDSQRFATKIKNA<br>SGSADPSKQKWISNPSKECP<br>KLFTSGPVCLKAKGVKFDP<br>S<br>DQELIWHLRAKHGKSGIKP<br>HPSIDEFIPTVEEDEGICYTH<br>PQKLPGVKQNGSVSHFFHR<br>T<br>FKAYNTGTRKRRKINTDDA<br>DVRWHKTGKTKPVLVDGK<br>QLGCKKIMVLYMSLAKGG<br>KVEKT<br>NWVMHQYHLGTGEDERDG<br>EYVVSKLFFQQQFKPGDKN<br>AQELTTSDDLESMAAEADL<br>PDFT<br>TLPADKHVTTSDDLESLAA<br>EADLPDFTTLPADKHVGTIQ<br>VVHNSEQNLYQVNRNCEIN<br>IE<br>ETVVLPPSEKTEDGDNPQSQ<br>DPKLWEGDSQFELLDTQQL<br>AEGLALCDEFLLSQSQTCG<br>GG<br>DEPTVTKPCLAAYAHLSAE<br>DFKKDLEECQRLAPTDNTN<br>LELENTNEFRLSQIDFSQDSF

Sspon.08G0010270-1A	*SsNAC110*	ATGACAAGGACAACTTGGATAAT<br>TGACAGCCAAAGATTTGCCACCA<br>AAATAAAAAATGCT<br>TCTGGGTCTGCAGATCCCAGTAA<br>ACAGAAATGGATCAGCAACCCAA<br>GTAAAGAGTGCCCA<br>AAGTGCAGCCATGTCATCGATAA<br>CAGTGATGTTGTTCACCAGTGGCC<br>CGGTTTGCCTAAA<br>GGTGTAAAATTTGATCCGTCTGAC<br>CAGGAATTAATCTGGCACTTACG<br>GGCAAAACATGGA<br>AAATCTGGTATAAAACCTCATCC<br>GTCCATTGACGAATTTATTCCAAC<br>AGTTGAGGAAGAT<br>GAAGGCATCTGCTACACTCATCC<br>ACAGAAACTTCCAGGTGTTAAGC<br>AAAATGGAAGTGTA<br>TCACATTTCTTCCACAGAACATTT<br>AAGGCCTACAACACTGGGACTAG<br>GAAGCGTCGAAAG<br>ATAAACACTGATGATGCTGATGT<br>CCGTTGGCACAAGACAGGCAAGA<br>CAAAGCCAGTATTA<br>GTTGATGGAAAACAGCTTGGCTG<br>TAAGAAAATAATGGTGCTTTATA<br>TGAGCCTGGCAAAG	MTRTTWIIDSQRFATKIKNA<br>SGSADPSKQKWISNPSKECP<br>KCSHVIDNSDVVHQWPGLP<br>K<br>GVKFDPSDQELIWHLRAKH<br>GKSGIKPHPSIDEFIPTVEED<br>EGICYTHPQKLPGVKQNGS<br>V<br>SHFFHRTFKAYNTGTRKRR<br>KINTDDADVRWHKTGKTK<br>PVLVDGKQLGCKKIMVLY<br>MSLAK<br>GGKVEKTNWVMHQYHLGT<br>GEDERDGEYVVSKLFFQQQ<br>FKPGDKNAQELTTSDDLES<br>MAAE<br>ADLPDFTTLPADKHVTTSD<br>DLESLAAEADLPDFTTLPAD<br>KHVGTIQVVHNSEQNLYQV<br>NR<br>NCEINIEETVVLPPSEKTEDG<br>DNPQSQDPKLWEGDSQFEL<br>LDTQQLAEGLALCDEFLLS<br>Q<br>SQTCGGGDEPTVTKPCLAA<br>YAHLSAEDFKKDLEECQRL<br>APTDNTNLELENTNEFRLSQ

Sspon.08G0016300-1A	*SsNAC111*	ATGAGCATCTCGGTGAACGGGCA<br>GTCGGTGGTGCCGCCGGGGTTCC<br>GGTTCCACCCGACG<br>GAGGAGGAGCTGCTGACCTACTA<br>CCTGAAGAAGAAGGTGGCGTCGG<br>AGCGCATCGACCTG<br>GACGTCATCCGCGACGTCGACCT<br>CAACAAGCTCGAGCCATGGGACA<br>TCCAAGAGAAGTGC<br>CGCATCGGTTCTGGCCCCCAGAA<br>CGACTGGTACTTCTTCAGCCACAA<br>GGACAAGAAGTAC<br>CCGACGGGGACGCGCACGAACCG<br>CGCCACCGCCGCCGGGTTCTGGA<br>AGGCCACGGGCCGC<br>GACAAGGCCATCTACGCGTCGGG<br>CGCCCGCCGCATCGGCATGCGCA<br>AGACGCTCGTCTTC<br>TACAAGGGCCGCGCTCCGCACGG<br>CCAGAAGTCCGACTGGATCATGC<br>ACGAGTACCGCCTC<br>GAGGCGGCGCTCGACGCCGACGC<br>CGGCCACAACCCCGCCGCCGGGG<br>CCGTCGACCATCCC<br>TACTACACCTCGCCGACGCCGCCT<br>CCTTCTACCGCAATCCGTGGCGCG<br>GCGGGAGACCAA	MSISVNGQSVVPPGFRFHPT<br>EEELLTYYLKKKVASERIDL<br>DVIRDVDLNKLEPWDIQEK<br>C<br>RIGSGPQNDWYFFSHKDKK<br>YPTGTRTNRATAAGFWKAT<br>GRDKAIYASGARRIGMRKT<br>LVF<br>YKGRAPHGQKSDWIMHEY<br>RLEAALDADAGHNPAAGA<br>VDHPYYTSPTPPPSTAIRGA<br>AGDQ<br>AAQEQEGWVICRVFKKKNL<br>VHHGQSSAGVTAAGSKMA<br>AAAVPMESSPSHCSSVTVIS<br>DHA<br>NKQQAQAMLQHSASDDAL<br>DHILQYMGGGKQPDTKPAL<br>LDQQQQHHHHHHLAAATT<br>TTAAC<br>PAGGGGLYGKFMKLPPLEH<br>AGGGGMLPTPPGACEYGVA<br>DASGIADWDALDRLAAYEL<br>NGL<br>SDASKNMAAFFDVEPSAAA<br>AFSSSTSSVHAAAVDGDLW<br>SLARSVSALHADLTMNNV

Sspon.08G0020420-1B	*SsNAC112*	ATGGATGGTGCAGCGGCGGCGGC<br>AACAGGCGGAAGCAGTGGCGGCC<br>ACCGCCACATGGAC<br>TCAAGGATCGAGGAGCATGGGAA<br>GTACCTGTCGGAGTCGAGCTGCT<br>GCCCACAGTGCGGC<br>CACAAGACCGACCGCAAGCTGGA<br>CTGGGTGGGGCTGCCGGCGGGGG<br>TGAAGTTCGACCCG<br>ACGGATCAGGAGCTGATTGAGCA<br>CCTGCAGGCCAAGGTCCGGGCAG<br>CTTCGGCGGCGGCG<br>CCGTCACACCCTCTGATCGACGA<br>GTTCATACCCACCATTGAGGGGG<br>AGGACGGCATATGC<br>TACACCCATCCTGAGAGACTGCC<br>AGGTTTGACAAAGGACGGCAGGA<br>GCAGGCACTTCTTC<br>CACCGGCCATCCAAGGCTTACAC<br>CACCGGCACTCGCAAGCGCCGCA<br>AGATTCATCAACCG<br>CCGGCCGCCGAGGGCTCATCCTC<br>CTCCGCCGTGCCCGCGCAGCAGC<br>AGCAGCAGCAGCGG<br>AGCGAGACGCGGTGGCACAAGAC<br>CGGCAAGACGCGGCCGGTGGTGG<br>TCGCCGGCCAGCAG	MDGAAAAATGGSSGGHRH<br>MDSRIEEHGKYLSESSCCPQ<br>CGHKTDRKLDWVGLPAGV<br>KFDP<br>TDQELIEHLQAKVRAASAA<br>APSHPLIDEFIPTIEGEDGICY<br>THPERLPGLTKDGRSRHFF<br>HRPSKAYTTGTRKRRKIHQ<br>PPAAEGSSSSAVPAQQQQQ<br>QRSETRWHKTGKTRPVVV<br>AGQQ<br>RGCKKILVLYTNFGKNRKP<br>DKTNWVMHQYHLGDKEEE<br>REGELVVSKIFYQTQPRQCG<br>GAA<br>EPATAAASSDTVEEAAADP<br>PVPEGMVAPPPDVAVAGAF<br>HGAAGIDEFNFAQFRSSFEE

Sspon.08G0021480-1B	*SsNAC113*	ATGACAGGGTGCGCCACCATCTC<br>CGATTCGACCATCCTCTCTCGTCC<br>CGCCGCGTTTCTC<br>CGTAGGAAGGACTGTCATCTGTTT<br>ATGTCGGAAAAAGGGAGCGTCTT<br>GGGCCGCGGGGCC<br>TACGGAGGATGCGTCCTTTTGGCA<br>TTTGTGTTGGGGGAATTGATATCC<br>GTTATCAATCGG<br>ACAACTTGGATAATTGACAGCCA<br>AAGATTTGCCACCAAAATAAAAA<br>ATGCTTCTGGGTCT<br>GCAGATCCCAGTAAACAGAAATG<br>GATCAGCAACCCAAGTAAAGAGT<br>GCCCAAAGTGCAGC<br>CATGTCATCGATAACAGTGATGTT<br>GTTCACCAGTGGCCCGGTTTGCCT<br>AAAGGTGTAAAA<br>TTTGATCCGTCTGACCAGGAATTA<br>ATCTGGCACTTACGGGCAAAACA<br>TGGAAAATCTGGT<br>ATAAAACCTCATCCGTCCATTGAC<br>GAATTTATTCCAACAGTTGAGGA<br>AGATGAAGGCATC<br>TGCTACACTCATCCACAGAAACTT<br>CCAGGTGTTAAGCAAAATGGAAG<br>TGTATCACATTTC	MTGCATISDSTILSRPAAFLR<br>RKDCHLFMSEKGSVLGRGA<br>YGGCVLLAFVLGELISVINR<br>TTWIIDSQRFATKIKNASGS<br>ADPSKQKWISNPSKECPKCS<br>HVIDNSDVVHQWPGLPKGV<br>K<br>FDPSDQELIWHLRAKHGKS<br>GIKPHPSIDEFIPTVEEDEGIC<br>YTHPQKLPGVKQNGSVSHF<br>FHRTFKAYNTGTRKRRKIN<br>TDDADVRWHKTGKTKPVL<br>VDGKQLGCKKIMVLYMSL<br>AKGGK<br>VEKTNWVMHQYHLGTGED<br>ERDGEYVVSKLFFQQQFKP<br>GDKNAQELTTSDDLESMAA<br>EADL<br>PDFTTLPADKHVTTSDDLES<br>LAAEADLPDFTTLPADKHV<br>GTIQVVHNSEQNLYQVNRN<br>CE<br>INIEETVVLPPSEKTEDGDNP<br>QSQDPKLWEGDSQFELLDT<br>QQLAEGLALCDEFLLSQSQT<br>CGGGDEPTVTKPCLAAYAH<br>LSAEDFKKDLEDCORLAPT

Sspon.08G0024550-1B	*SsNAC114*	ATGCAGCCGCCGCCGCAGCTGCC<br>GGCGGCTTTGCCGGTAGGGTTCC<br>GCTTCCGCCCGACG<br>GACGAGGAGCTGGTGCGGCACTA<br>CCTCAAGCCCAAGATCGCGGGTC<br>GCGCGCACGCGGAC<br>CTGCTCCTTATCCCCGATGTCGAC<br>CTCTCGGCGTGCGAGCCCTGGGA<br>GCTGCCCGCCAAG<br>GCCCTCATCAGGTCCGACGACCC<br>CGAGTGGTTCTTCTTCGCGCCCCT<br>CGACCGCAAGTAC<br>CCCGGCGGCCACCGCTCCAACCG<br>CTCCACCGCTGCCGGGTACTGGA<br>AGGCCACGGGCAAG<br>GACCGCCTCATCCGCTCGCGCCCA<br>GCAGGCACCCTCATCGGCGTCAA<br>GAAGACGCTCGTC<br>TTCCACAGAGGGAGGGCGCCCCG<br>AGGCCACCGCACCGCCTGGATCA<br>TGCACGAGTACCGC<br>ACGGCCGAGCCCCAGCTCCAGCA<br>AGGCCAAAACGGTAGCTTCGTGC<br>TCTACCGCCTATTC<br>AACAAGAACGAGGAGGAATCCGA<br>AGCCTCAGACGCCGCCGATTCAC<br>CGTCCACATCGTCT	MQPPPQLPAALPVGFRFRPT<br>DEELVRHYLKPKIAGRAHA<br>DLLLIPDVDLSACEPWELPA<br>K<br>ALIRSDDPEWFFFAPLDRKY<br>PGGHRSNRSTAAGYWKAT<br>GKDRLIRSRPAGTLIGVKKT<br>LV<br>FHRGRAPRGHRTAWIMHEY<br>RTAEPQLQQGQNGSFVLYR<br>LFNKNEEESEASDAADSPST<br>SS<br>PAIAPAVKAENLSQPASAQ<br>MSHLLATCIDSGCDWYLKQ<br>GDDHLLDVLAQLPDLQPEH<br>TFD<br>GFPTITSPMRPYTDHPFLGN<br>AGEQDLSAYIDSIIAHQDLE<br>DLLASPSLADMVEQATVNV<br>E<br>PHPTSLPVLDNNSNSKRLAE<br>NGRGKVGSETLLLIKGTDG<br>TGASACCSSATKILQADEDN<br>A<br>NHHTGAQSNLASNASAQVS<br>HFYNQYQLQSTFIPETEPPN<br>SGALCSAASSTPYPQHLFNG

Sspon.08G0029670-1D	*SsNAC115*	ATGGCAGCGACGGCATGCGTAGC<br>CAGCGGGCTCCCGCCGGGCATTC<br>TGTTTTCCCCCGAA<br>GATGAGGTGGCGGTGGGGCACTA<br>CCTCCTTCCTCGCCTCCAGGGCTG<br>GCCGCTTCCCATC<br>GAAGGCCTCATCCTCGATGACGA<br>CCCGCTGAGCGCGGCTCCGTGGG<br>AGCTCCTGGAGCGG<br>AATGGGCGCAAGGAGCAGGCTTT<br>CTTTTTCGCGGAGGGGCAGGCGA<br>GGTGTGGGAAGGGC<br>ACGCGACAGAGGCGAACCTGCGC<br>GGGCGGCGGATGGTGGGAGGGGC<br>AGAAGACGTGCGCG<br>GAAGGCCACAAGCTGCGCGTCCC<br>TGGCGGCGGCGGCGGCAAGGAGG<br>CCGCGTGGCGGAAG<br>AAAGCCTTCAACTTCCACAGCGG<br>CAGCGGCGGCGACGGGAAGCGCA<br>ACACGGGATGGGTG<br>ATGCACGAGTACGCGGTCACCGG<br>CCCGGAGGATCTGGCCTGCTCGC<br>CGCTCAGGCTGTAC<br>CACATCCGGCTCAGCAGCTACGG<br>GAGAAAGCAGTGCGGCGCCATGG<br>AGGTGCTGCCGGGG	MAATACVASGLPPGILFSPE<br>DEVAVGHYLLPRLQGWPLP<br>IEGLILDDDPLSAAPWELLE<br>R<br>NGRKEQAFFFAEGQARCGK<br>GTRQRRTCAGGGWWEGQK<br>TCAEGHKLRVPGGGGGKE<br>AAWRK<br>KAFNFHSGSGGDGKRNTG<br>WVMHEYAVTGPEDLACSP<br>LRLYHIRLSSYGRKQCGAM<br>EVLPG<br>LGFLLGFVFAPEDSGIVVHY<br>LLPRMLGQPLLLDGLILDDD<br>PLSAPPWELLERNGRRETPS<br>SSPWASQEQQGLPAEADLR<br>GRRVLERGEDARRRREAVR<br>PRQYGGGGVAEESAQLPGR<br>RRR<br>KGGQHGVGDAQVRDHRPR<br>PPGRVAAKAVSHPVHGHG<br>KKRKQRGDGVGGAGDSCG<br>DEPARN<br>EAWRRCVAEDDALLHMSSP<br>QQPISSSTVLIGHNCINGDGA<br>DHHAAPVTLLAPGIVDTNC<br>D
