# Supplementary material for: Genome-wide identification and expression analysis of the NAC transcription factor family in Saccharum spontaneum under different stresses
Source: Plant Signal Behav. 2022 Jun 22;17(1):2088665. doi: 10.1080/15592324.2022.2088665 (PMC9225438; doi:10.1080/15592324.2022.2088665)
Supplement: Supplemental Material [file KPSB_A_2088665_SM8717.zip › Supplementary Materials/Table S3.pdf]

Table S3. Protein property of SsNAC proteins in *S. spontaneum*

Gene ID	Gene name	Exon number	Number of amino acid	Molecular weight Mw/Da	Theoretical pI	Number of negatively amino acid	Number of positively amino acid	Instability index	Aliphatic index	Grand average of hydropathicity	Location prediction
Sspon.01G0001190-1A	*SsNAC001*	5	683	75812.38	5.8	90	74	43.5	71.24	-0.451	nucleus
Sspon.01G0001940-1A	*SsNAC002*	2	319	35137.69	7.85	37	38	49.49	68.37	-0.46	nucleus
Sspon.01G0007700-1A	*SsNAC003*	11	666	74739.39	6.21	97	90	55.17	77.91	-0.513	nucleus
Sspon.01G0008840-1A	*SsNAC004*	2	351	38655.63	5.67	45	37	43.84	71.2	-0.433	nucleus
Sspon.01G0008870-1A	*SsNAC005*	2	371	40365.57	9.03	31	37	42.62	60.67	-0.5	nucleus
Sspon.01G0013390-1A	*SsNAC006*	4	380	41995.21	6.19	49	44	42.65	69.92	-0.531	nucleus
Sspon.01G0015060-1A	*SsNAC007*	5	338	36291.86	5.67	36	30	48.22	69.38	-0.22	nucleus
Sspon.01G0023330-1A	*SsNAC008*	1	226	24716.00	7.72	21	22	48.39	66.06	-0.267	nucleus
Sspon.01G0023640-1A	*SsNAC009*	2	304	33324.08	8.93	28	34	37.88	60.39	-0.382	nucleus
Sspon.01G0029280-1A	*SsNAC010*	2	354	39526.77	8.73	42	46	46.97	64.27	-0.521	nucleus

Sspon.01G0030860-3D	*SsNAC011*	6	3849	425609.23	5.71	483	406	46.34	80.67	-0.286	nucleus
Sspon.01G0031460-1A	*SsNAC012*	2	719	78229.24	5.54	93	71	58.14	57.66	-0.598	nucleus
Sspon.01G0031480-3D	*SsNAC013*	6	1527	167478.62	4.89	230	136	47.35	58.61	-0.648	nucleus
Sspon.01G0032330-1A	*SsNAC014*	13	959	105747.17	8.78	110	124	51.82	64.77	-0.52	nucleus
Sspon.01G0034390-1B	*SsNAC015*	2	378	41584.5	5.28	55	45	45.38	65.77	-0.495	nucleus
Sspon.01G0042440-1B	*SsNAC016*	1	319	35429.86	6.33	44	41	34.38	61.29	-0.64	nucleus
Sspon.01G0059240-1D	*SsNAC017*	3	322	34953.53	8.12	33	35	50.97	65.5	-0.342	nucleus
Sspon.01G0063470-1D	*SsNAC018*	2	668	70985.06	8.39	67	72	53.35	52.28	-0.597	nucleus
Sspon.02G0005070-1A	*SsNAC019*	2	416	44168.36	7.73	38	39	38.95	65.87	-0.387	nucleus
Sspon.02G0008330-1A	*SsNAC020*	8	681	77634.27	6.12	101	93	40.97	64.05	-0.732	nucleus
Sspon.02G0008350-1A	*SsNAC021*	2	159	18379.04	10.21	15	30	38.31	62.58	-0.762	nucleus
Sspon.02G0010630-1A	*SsNAC022*	2	261	28341.08	10.06	24	37	65.05	66.17	-0.553	nucleus

Sspon.02G0011250-1A	*SsNAC023*	2	346	37300.05	7.36	32	32	43.68	61.07	-0.532	nucleus
Sspon.02G0011260-1A	*SsNAC024*	3	705	77043.48	4.58	99	45	48.54	70.33	-0.473	nucleus
Sspon.02G0013400-1A	*SsNAC025*	2	456	50001.27	7.90	50	52	53.12	58.22	-0.675	nucleus
Sspon.02G0014720-1T	*SsNAC026*	2	361	40424.53	8.92	38	45	46.28	66.76	-0.667	nucleus
Sspon.02G0015260-1A	*SsNAC027*	2	362	39706.20	6.02	38	35	37.97	62.29	-0.453	nucleus
Sspon.02G0020600-1A	*SsNAC028*	2	165	18611.36	9.89	19	31	30.39	63.39	-0.693	nucleus
Sspon.02G0027090-1A	*SsNAC029*	2	258	27735.37	9.85	30	43	59.74	62.21	-0.556	nucleus
Sspon.02G0031110-1A	*SsNAC030*	2	309	33652.10	8.65	31	35	36.91	63.53	-0.441	nucleus
Sspon.02G0032050-1A	*SsNAC031*	4	278	32353.87	6.65	42	39	40.09	60.68	-0.978	nucleus
Sspon.02G0036100-1B	*SsNAC032*	3	204	23909.05	8.50	28	31	49.25	59.26	-0.886	nucleus
Sspon.02G0037950-1B	*SsNAC033*	1	304	33122.10	5.89	38	30	50.45	66.51	-0.476	nucleus
Sspon.02G0038600-1B	*SsNAC034*	11	1974	214275.76	8.72	196	217	49.00	88.16	-0.216	nucleus

Sspon.03G0000160-1A	*SsNAC035*	2	260	27616.99	8.89	27	32	58.52	62.85	-0.485	nucleus
Sspon.03G0001670-1A	*SsNAC036*	1	446	47156.44	8.86	38	43	51.07	58.43	-0.479	nucleus
Sspon.03G0001860-1A	*SsNAC037*	1	295	32675.34	8.47	34	37	55.42	68.88	-0.472	nucleus
Sspon.03G0002180-1A	*SsNAC038*	2	305	32778.37	5.29	38	29	53.46	61.74	-0.572	nucleus
Sspon.03G0009960-1A	*SsNAC039*	2	314	34474.80	8.95	32	38	28.22	66.91	-0.621	nucleus
Sspon.03G0011440-1A	*SsNAC040*	0	259	27416.46	8.27	32	33	37.14	55.87	-0.710	nucleus
Sspon.03G0011450-1A	*SsNAC041*	0	363	38721.65	6.24	55	51	59.54	53.94	-0.818	nucleus
Sspon.03G0017190-1A	*SsNAC042*	4	526	57869.53	5.57	68	54	44.79	70.00	-0.568	nucleus
Sspon.03G0017800-1A	*SsNAC043*	0	257	28087.05	5.72	34	27	57.77	57.00	-0.737	nucleus
Sspon.03G0018260-1A	*SsNAC044*	2	387	41994.23	6.15	38	36	36.45	63.88	-0.282	nucleus
Sspon.03G0022860-1A	*SsNAC045*	6	499	55678.51	8.03	55	57	52.02	60.04	-0.768	nucleus
Sspon.03G0025270-1A	*SsNAC046*	2	378	40692.52	5.99	46	37	33.66	65.66	-0.376	nucleus

Sspon.03G0026820-1B	*SsNAC047*	0	212	23564.27	5.78	33	30	65.28	58.02	-0.688	nucleus
Sspon.03G0031890-1B	*SsNAC048*	0	312	34281.97	7.12	45	45	49.05	60.13	-0.713	nucleus
Sspon.03G0035360-1B	*SsNAC049*	2	358	38961.81	5.16	39	32	37.02	65.22	-0.280	nucleus
Sspon.03G0040250-1P	*SsNAC050*	6	1011	108853.95	6.04	116	101	40.54	80.20	-0.246	nucleus
Sspon.03G0040660-1C	*SsNAC051*	1	331	36021.21	5.90	48	42	63.82	68.42	-0.655	nucleus
Sspon.04G0000480-1A	*SsNAC052*	4	653	71255.57	4.56	97	55	41.90	68.01	-0.534	nucleus
Sspon.04G0003490-1A	*SsNAC053*	2	387	43439.20	8.83	39	45	50.35	71.60	-0.527	nucleus
Sspon.04G0003500-4D	*SsNAC054*	6	653	72042.37	7.94	56	58	44.61	83.35	-0.105	nucleus
Sspon.04G0004240-3D	*SsNAC055*	19	1305	143171.82	6.10	169	154	49.56	84.14	-0.306	nucleus
Sspon.04G0007420-1A	*SsNAC056*	2	336	37576.07	5.89	43	36	51.98	58.72	-0.666	nucleus
Sspon.04G0009100-1A	*SsNAC057*	3	269	30225.11	6.59	37	35	46.03	72.16	-0.737	nucleus
Sspon.04G0010300-1A	*SsNAC058*	3	775	85077.23	6.36	82	73	46.04	85.23	-0.067	nucleus

Sspon.04G0014100-1A	*SsNAC059*	3	316	35249.96	6.11	35	27	50.43	72.85	-0.434	nucleus
Sspon.04G0022660-1B	*SsNAC060*	20	764	84672.72	6.62	91	87	41.16	86.16	-0.294	nucleus
Sspon.04G0024580-1B	*SsNAC061*	4	575	61771.84	5.41	56	41	49.35	80.07	-0.095	nucleus
Sspon.04G0031350-2D	*SsNAC062*	15	1643	184094.61	8.95	176	198	53.25	78.71	-0.376	nucleus
Sspon.04G0035420-1D	*SsNAC063*	15	1296	140161.93	6.5	136	129	51.92	75.12	-0.258	nucleus
Sspon.04G0035430-1D	*SsNAC064*	1	440	46869.25	5.4	41	31	51.53	65.61	-0.305	nucleus
Sspon.05G0006290-1A	*SsNAC065*	2	329	38253.03	6.02	46	39	42.98	64.29	-0.762	nucleus
Sspon.05G0007380-2C	*SsNAC066*	3	432	46411.03	8.64	51	55	50.32	64.03	-0.541	nucleus
Sspon.05G0008880-2C	*SsNAC067*	6	452	49676.85	5.1	62	43	52.6	66.86	-0.569	nucleus
Sspon.05G0008900-2D	*SsNAC068*	4	498	54411.61	6	64	56	55.81	70.5	-0.453	nucleus
Sspon.05G0009100-1A	*SsNAC069*	0	232	25665.66	5.49	37	31	46.87	62.24	-0.629	nucleus
Sspon.05G0010800-1A	*SsNAC070*	2	338	37039.94	6.37	40	35	31.92	51.45	-0.699	nucleus

Sspon.05G 0013150-1A	*SsNAC071*	2	279	30830.49	5.78	33	29	60.36	55.23	-0.535	nucleus
Sspon.05G 0015240-1A	*SsNAC072*	7	471	51159.69	6.85	46	43	49.57	80.32	-0.39	nucleus
Sspon.05G 0019180-1A	*SsNAC073*	1	315	34359.57	6.25	31	27	56.32	72.16	-0.34	nucleus
Sspon.05G 0019300-1A	*SsNAC074*	21	1158	123248.62	6.85	122	199	52.57	75.92	-0.215	nucleus
Sspon.05G 0020040-1A	*SsNAC075*	0	363	39983.23	4.94	51	31	43.85	60.28	-0.588	nucleus
Sspon.05G 0020640-1A	*SsNAC076*	2	372	40359.90	6.73	37	35	37.2	55.16	-0.553	nucleus
Sspon.05G 0020650-1A	*SsNAC077*	2	403	44410.23	6.53	38	35	50.11	61.51	-0.674	nucleus
Sspon.05G 0023210-1B	*SsNAC078*	2	308	33539.49	5.82	35	30	49.38	63.44	-0.461	nucleus
Sspon.05G 0024050-1B	*SsNAC079*	1	182	20685.92	5.06	35	26	48.88	47.2	-1.037	nucleus
Sspon.05G 0025100-2C	*SsNAC080*	8	497	54319.69	8.1	57	59	55.96	75.6	-0.324	nucleus
Sspon.05G 0027840-1B	*SsNAC081*	7	541	58126.23	7.71	50	51	52.47	71.07	-0.484	nucleus
Sspon.05G 0029190-1B	*SsNAC082*	2	162	18396.21	9.6	16	26	29.71	65.74	-0.54	nucleus

Sspon.05G0029330-1B	*SsNAC083*	1	326	35910.24	4.76	49	28	41.21	60	-0.656	nucleus
Sspon.05G0032850-1C	*SsNAC084*	1	169	18619.81	6.48	15	13	37.77	62.31	-0.371	nucleus
Sspon.06G0000010-1A	*SsNAC085*	1	366	40208.66	5.51	48	40	70.43	59.73	-0.672	nucleus
Sspon.06G0001130-1A	*SsNAC086*	2	236	25018.76	10.31	19	35	68.36	74.87	-0.267	nucleus
Sspon.06G0001780-1A	*SsNAC087*	1	331	36215.49	6.86	32	31	42.87	61.45	-0.415	nucleus
Sspon.06G0005910-1A	*SsNAC088*	1	159	17940.33	9.4	19	25	52.01	60.19	-0.655	nucleus
Sspon.06G0009610-1A	*SsNAC089*	7	312	34629.5	9.24	32	41	41.86	70.67	-0.395	nucleus
Sspon.06G0012880-1A	*SsNAC090*	3	307	34272.55	9.68	31	44	52.08	55.02	-0.738	nucleus
Sspon.06G0013480-1A	*SsNAC091*	1	287	31520.26	7.12	29	29	57.05	63.31	-0.572	nucleus
Sspon.06G0024580-1B	*SsNAC092*	2	434	45332.15	6.3	49	42	40.38	66.15	-0.404	nucleus
Sspon.06G0028920-1C	*SsNAC093*	4	671	72353.12	4.62	88	49	39.74	72.3	-0.426	nucleus
Sspon.07G0001320-2B	*SsNAC094*	1	542	60528.14	4.89	81	52	57.76	66.01	-0.84	nucleus

Sspon.07G0007590-1A	*SsNAC095*	7	552	60720.06	4.91	70	46	55.61	64.51	-0.451	nucleus
Sspon.07G0011330-1A	*SsNAC096*	6	361	40569.6	8.91	46	54	54.43	53.46	-0.914	nucleus
Sspon.07G0017230-1A	*SsNAC097*	4	569	61001.8	5.61	70	62	67.92	65.54	-0.398	nucleus
Sspon.07G0019480-2C	*SsNAC098*	1	590	62639.76	6.02	61	50	59.12	70.64	-0.396	nucleus
Sspon.07G0020380-1A	*SsNAC099*	2	401	42251.59	8.51	35	38	38.02	70.2	-0.266	nucleus
Sspon.07G0024330-1B	*SsNAC100*	3	466	50911.04	7.91	51	53	37.16	61.2	-0.48	nucleus
Sspon.07G0034490-1C	*SsNAC101*	1	574	61326.57	5.94	65	51	54.46	63.62	-0.521	nucleus
Sspon.07G0034910-1C	*SsNAC102*	4	616	66683.88	5.58	74	61	79.64	70.65	-0.42	nucleus
Sspon.07G0036500-1D	*SsNAC103*	2	321	35634.88	7.09	31	31	39.73	65.61	-0.442	nucleus
Sspon.08G0000770-1A	*SsNAC104*	1	196	21445.19	9.57	21	29	36.44	60.87	-0.618	nucleus
Sspon.08G0003810-1A	*SsNAC105*	2	304	33926.45	8.17	33	35	45.25	61.28	-0.495	nucleus
Sspon.08G0007270-2C	*SsNAC106*	3	430	47175.22	6.41	51	43	57.52	59.05	-0.758	nucleus

Sspon.08G 0010220-1A	*SsNAC107*	5	453	50740.24	4.99	73	46	43.18	65.01	-0.758	nucleus
Sspon.08G 0010230-4D	*SsNAC108*	7	681	76080.45	5.63	92	68	40.50	62.86	-0.754	nucleus
Sspon.08G 0010260-1A	*SsNAC109*	6	430	48114.67	5.12	69	49	39.24	66.23	-0.73	nucleus
Sspon.08G 0010270-1A	*SsNAC110*	6	437	48982.5	5.07	71	48	38.72	66.27	-0.751	nucleus
Sspon.08G 0016300-1A	*SsNAC111*	2	416	44506.81	6.59	43	39	43.82	67.74	-0.411	nucleus
Sspon.08G 0020420-1B	*SsNAC112*	2	298	32473.18	6.92	38	37	56.96	55.77	-0.735	nucleus
Sspon.08G 0021480-1B	*SsNAC113*	6	502	55582.25	5.27	74	53	37.77	73.23	-0.561	nucleus
Sspon.08G 0024550-1B	*SsNAC114*	3	543	59259.23	5.32	65	44	48.59	77.18	-0.428	nucleus
Sspon.08G 0029670-1D	*SsNAC115*	0	516	55489.55	6.63	59	56	61.68	69.84	-0.446	nucleus
