# Supplementary material for: Genome-wide identification and expression analysis of the NAC transcription factor family in Saccharum spontaneum under different stresses
Source: Plant Signal Behav. 2022 Jun 22;17(1):2088665. doi: 10.1080/15592324.2022.2088665 (PMC9225438; doi:10.1080/15592324.2022.2088665)
Supplement: Supplemental Material [file KPSB_A_2088665_SM8717.zip › Supplementary Materials/Table S4.pdf]

Table S4. The structural features of 10 motif in the protein sequences of *SsNAC* .

Name	E-value	Sites	Width	LLR
Motif 1	6.0e-1131	55	31	3660
	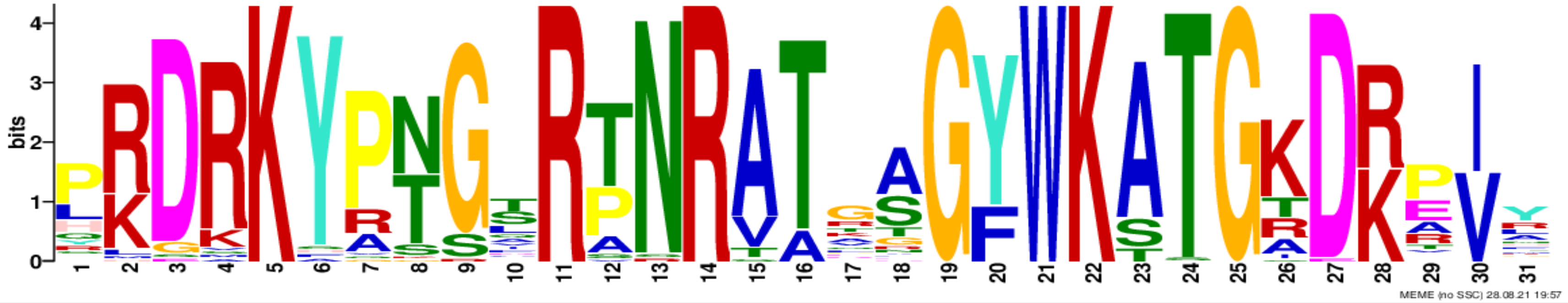 			
Motif 2	1.9e-1244	106	21	4212
	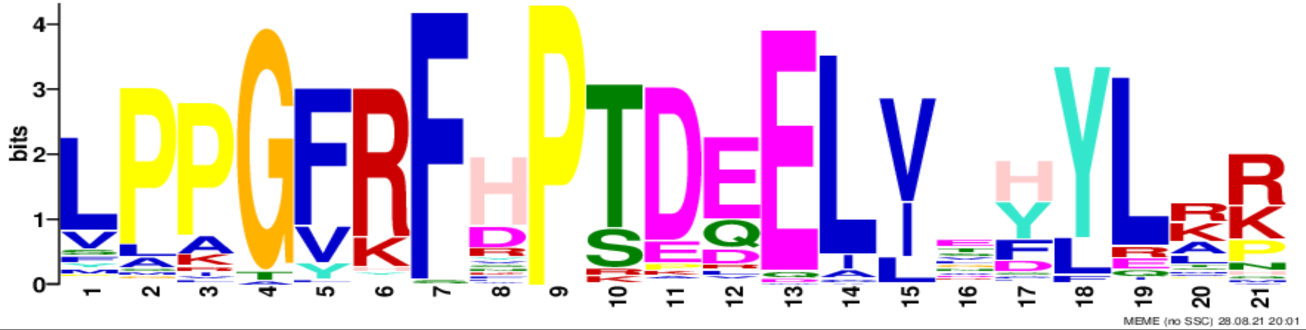 			
	6.3e-794	103	15	2970

Motif 3	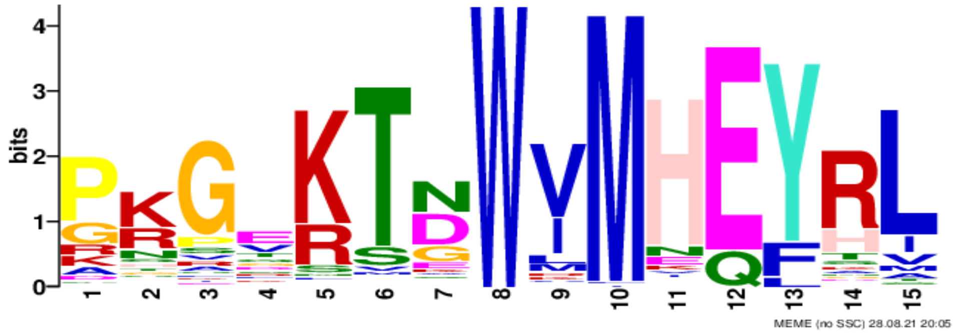			
	3.1e-641	16	65	2428
Motif 4	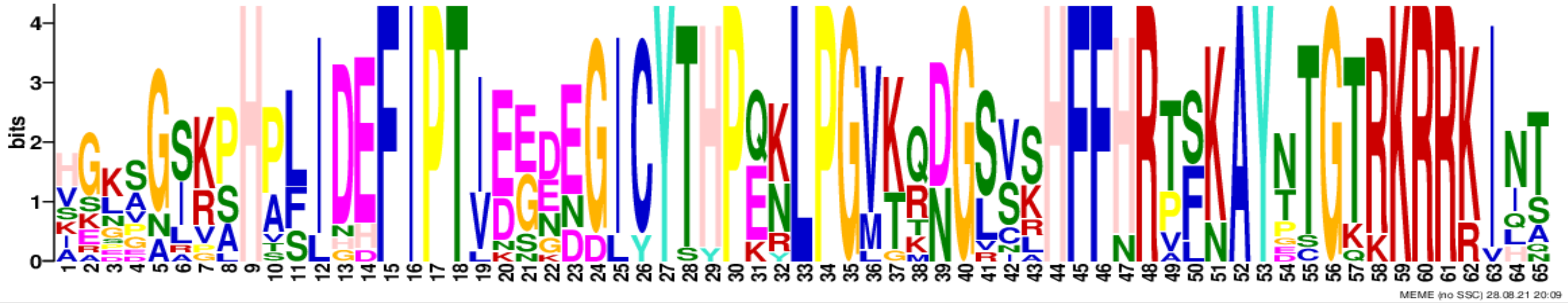			
	5.9e-623	73	21	2499
Motif 5	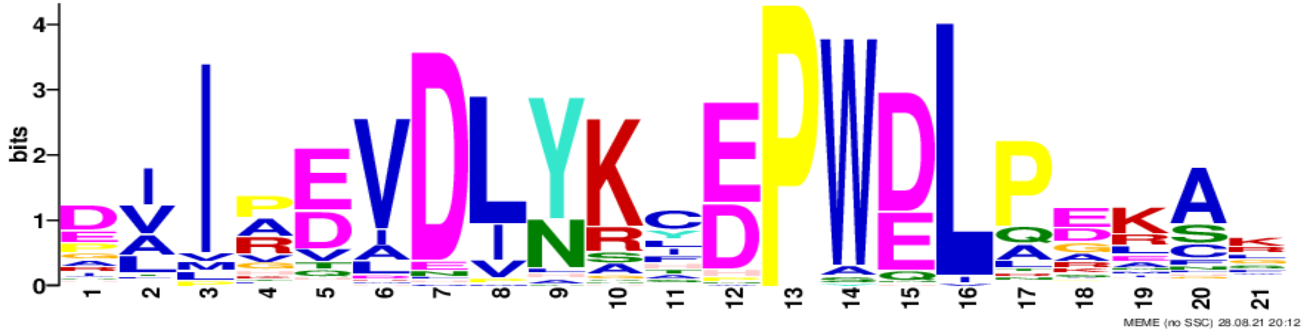			
	2.4e-471	83	11	1967

<table>
<tr><td rowspan="2">Motif 6</td><td colspan="4">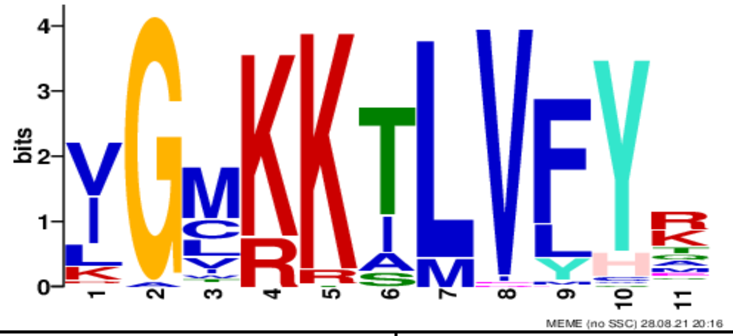</td></tr>
<tr><td>1.2e-353</td><td>97</td><td>14</td><td>1909</td></tr>
<tr><td rowspan="2">Motif 7</td><td colspan="4">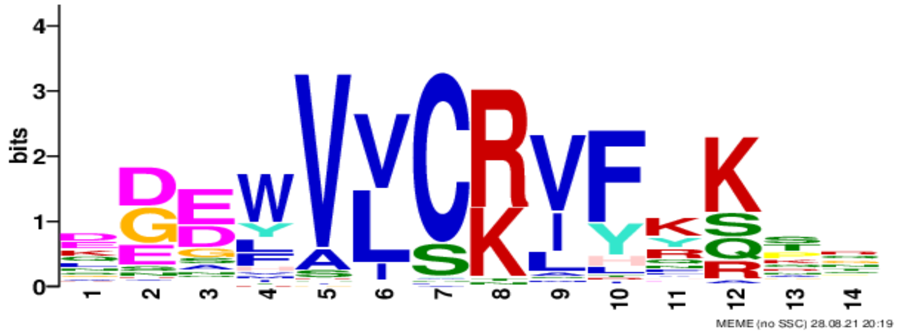</td></tr>
<tr><td>4.6e -309</td><td>5</td><td>100</td><td>1449</td></tr>
<tr><td rowspan="2">Motif 8</td><td colspan="4">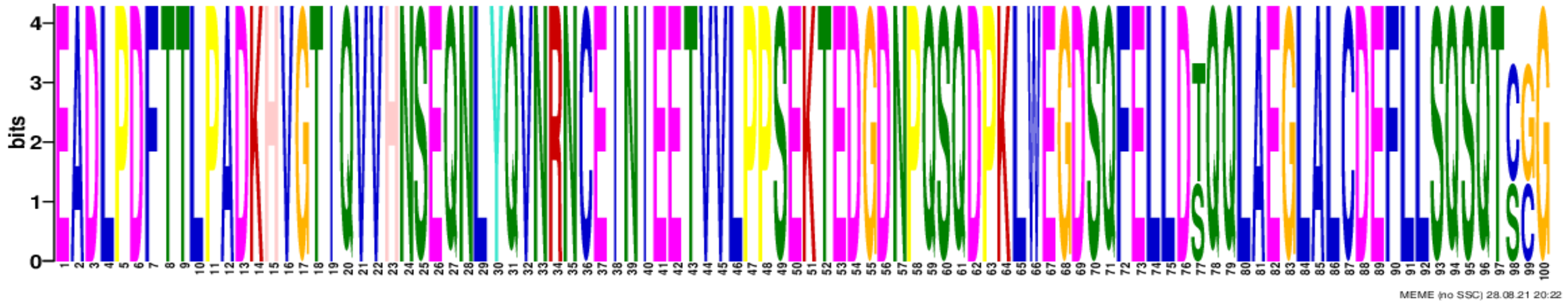</td></tr>
<tr><td>1.50E-228</td><td>73</td><td>8</td><td>1254</td></tr>
</table>

Motif 9	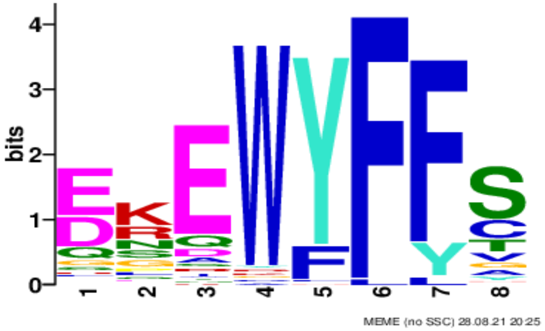			
Motif 10	4.60E-173	10	41	929
	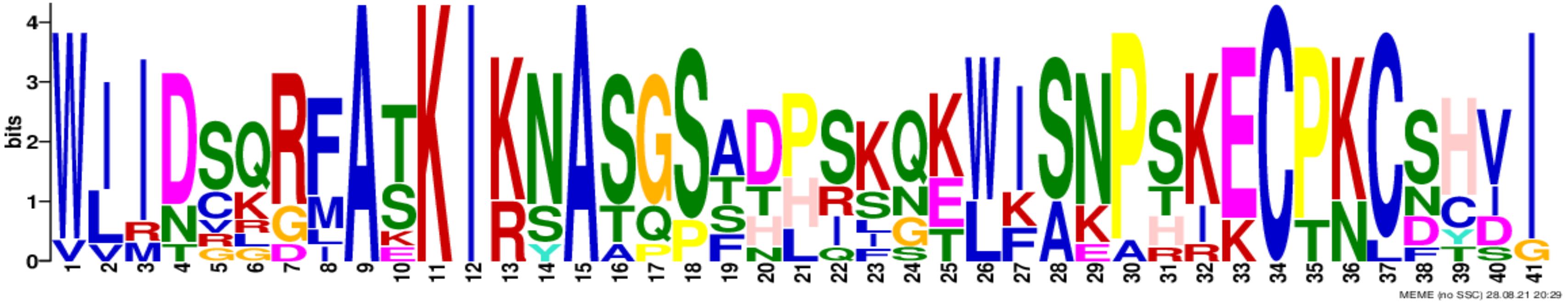			
