# Supplementary material for: Genome-wide identification and expression analysis of the NAC transcription factor family in Saccharum spontaneum under different stresses
Source: Plant Signal Behav. 2022 Jun 22;17(1):2088665. doi: 10.1080/15592324.2022.2088665 (PMC9225438; doi:10.1080/15592324.2022.2088665)
Supplement: Supplemental Material [file KPSB_A_2088665_SM8717.zip › Supplementary Materials/Table S5.pdf]

Table S5. Expression (FPKM) of SsNAC genes in different tissues.

Gene name	mature-leaf-roll	mature-leaf	mature-stem3	mature-stem6	mature-stem9	pre-leaf-roll	pre-leaf	pre-stem-3	pre-stem-6	pre-stem-9	seedling-leaf	seedling-stem
SsNAC001	0	0	1.88921	0	0	0	0	0.993014	0	0	0.045235667	1.500393333
SsNAC002	0.6584	0.672563	0.350447	0.328121	0.511111	0.120222	0	0.383745	0.165857	0.202326	0.665321667	0.527863333
SsNAC003	25.0941	23.5902	5.07169	9.28762	7.23594	16.361	16.8113	4.56544	10.9273	10.7301	3.022423333	2.027113333
SsNAC004	1.90573	3.85952	0	0	0.0417866	0	0.369229	0	0	0	0.395673667	0
SsNAC005	0	0	0.0401403	0	0	0	0	0	0	0	0	0.0771812
SsNAC006	0.137478	0.0684797	1.55778	0.991741	0.305604	0.480771	0.261958	1.28255	0.342753	0.404627	0.14892	0.265467333
SsNAC007	0	0	0.158897	0	0	0	0	0	0	0	0	0
SsNAC008	0	0	0	0	0	0	0	0	0	0	0.249043333	0.077527167
SsNAC009	0	0	0	0	0	0	0	0	0	0	0	0
SsNAC010	0	0	0	0	0	0	0	0	0	0	0	0
SsNAC011	0	0	0	0	0	0	0	0.35312	0	0	0	0.0535779
SsNAC012	8.47499	6.84334	43.4077	4.22062	0.404569	3.85267	5.19543	32.7479	11.123	7.76604	1.216230333	0.470217667
SsNAC013	113.891	119.498	56.7594	46.4546	13.6855	62.4928	86.3161	41.2688	15.0623	8.98037	7.742426667	7.963296667
SsNAC014	0.074412	0.0498875	0.146472	0.053852	0	0	0	0.294527	0.243967	0	0.0221484	2.10962
SsNAC015	0.20085	0	0.90909	0.204427	0.100635	0	0	0.448041	0	0	0.253527667	0.0271835
SsNAC016	3.30956	2.83408	3.28699	4.66563	3.58566	1.96788	1.99718	1.41233	2.91295	2.81451	2.63244	3.585703333
SsNAC017	0	0	0	0	0	0	0	0	0	0	0	0
SsNAC018	0	0	0	0	0	0	0	0	0	0	0	0
SsNAC019	0.02898	0.455071	0	0	0.509981	0	0	0	0	0	0	0
SsNAC020	0	0	0.305115	0	0	0	0	0.92891	0.0279001	0	0.003310563	0.255232667
SsNAC021	0	0	0.278001	0	0	0	0	0.602354	0	0	0	0.098244333
SsNAC022	2.21707	0.943262	5.96681	21.3928	23.2275	0	0.482247	4.27426	4.71383	0	0.944165	3.600466667
SsNAC023	0	0.0254817	0.0898712	0.347671	0.205457	0	0	0.100197	1.33035	0.786697	0.084889567	0.489149667
SsNAC024	6.098	4.67424	5.82307	4.13633	2.57017	3.43438	4.95024	6.17651	5.07135	3.40478	2.568736667	2.5513
SsNAC025	0	0	0	0	0	0	0	0	0	0	0.0218361	0
SsNAC026	0.086684	0	0	0	0	0	0	0	0.12574	0	0	0
SsNAC027	0	0	0	0	0	0	0	0	0	0	0	0
SsNAC028	15.7848	15.8926	2.25997	8.73766	7.1459	12.5236	12.5105	3.60384	2.90552	3.26423	0.877146333	2.126676667
SsNAC029	21.8779	23.1343	2.73259	24.2421	36.8553	55.5308	53.6351	12.6481	11.9939	23.572	18.5436	7.79804
SsNAC030	2.5162	4.10712	0	0.376255	0.15873	0	0	0	0.471383	0.372015	1.248591333	0.716593
SsNAC031	0	0	0	0	0	0	0	0	0	0	0.286662	0
SsNAC032	0	0	0.416091	0	0	0	0	0	0	0	0.064445667	0.546686667
SsNAC033	0	0	0.0529929	0	0	0	0	0	0	0	0	0.046579
SsNAC034	0.026034	0.0292894	0.0391317	0	0.012993	0	0	0	0	0.0809227	0.0131881	0
SsNAC035	3.42896	3.4782	11.5944	17.6823	13.7528	4.46642	5.27317	13.1102	19.8995	12.3215	7.60095	8.8077

SsNAC036	0.735992	1.98374	0.681214	1.53174	1.44133	0.40297	1.65754	0.785316	1.53407	1.22441	1.274096667	1.880236667
SsNAC037	49.4744	60.4977	4.46024	9.91364	6.25682	19.8982	40.198	5.02816	6.04727	3.02952	8.017153333	2.84073
SsNAC038	9.18362	9.23544	0.158303	1.69551	0	2.70413	1.96785	0	0.320531	0	1.470293333	0
SsNAC039	0.129208	0.615794	20.8629	33.5342	27.7293	0.790557	0	4.28587	5.7004	1.78512	1.629712333	19.14303333
SsNAC040	2.64E-08	0	0	0	0	0	0	0	0	0	0	0
SsNAC041	2.81193	3.04898	3.69536	1.79388	0.43102	1.75223	1.33781	2.62813	0.124762	0.442525	0.307692	0.194008233
SsNAC042	90.3074	88.2508	32.4137	25.2238	22.4279	84.3744	124.99	20.8785	37.0722	44.8177	12.03157	5.48203
SsNAC043	0	0	0	0	0	0	0	0	0	0	0	0
SsNAC044	0	0	0.704305	0	0	0	0	1.70845	0	0	0.012698167	0.121749333
SsNAC045	4.02932	3.54907	5.12925	18.525	16.6159	1.4907	3.18247	0.861132	11.5781	10.5679	3.498583333	8.398836667
SsNAC046	0	0	0	0	0	0	0	0	0	0	0	0
SsNAC047	0.180307	0	0.523597	0.184758	0	0	0	0.92011	2.39054	1.42226	0.113773667	0.562848
SsNAC048	0	0	0	0	0	0	0	0	0	0	0	0
SsNAC049	0	0	0.172259	0	0	0	0	0	0	0	0.041901	0
SsNAC050	0.981191	1.28682	0.65217	0.892688	1.10929	0.347608	0.900894	0.702932	0.496441	0.445655	0.371734667	0.398706333
SsNAC051	0.353843	0.557241	6.02E-07	0.040182	0.0322291	0	0.145	0	0	0	0.015846805	2.75E-08
SsNAC052	8.04158	10.085	10.7022	10.6543	8.21668	9.6115	7.01337	10.1256	8.00389	8.53417	5.891253333	6.168996667
SsNAC053	0	0	0.0785036	0.026903	0	0	0	0.685847	0	0	0	0.011889467
SsNAC054	0.285054	0.0354021	0.343194	0	0	0	0	0	0	0	0.030338133	0.289659333
SsNAC055	0.878937	1.33187	0.250466	0.130583	0.0642139	0.322847	0.302317	0	0	0	0.059964133	0.034319367
SsNAC056	0.106033	0.198468	0.158633	0.125221	0.194232	0	0	0.142856	0.0959269	0.226157	0.017789967	0.200850167
SsNAC057	0	0	0	0	0	0	0	0	0	0	0	0
SsNAC058	0.471215	0.64895	0.397116	0.369128	0.38214	0	0	0	0.492005	0.169114	0.894782667	0.833010333
SsNAC059	0.345877	0.388004	0.343957	0.425641	0.411674	0.76552	0.625419	0	0.273032	0.161052	0.352587667	0.597051333
SsNAC060	9.94816	9.18342	4.88221	8.60593	4.43318	8.62258	11.6408	3.05907	6.89297	9.10848	12.0317	3.44333
SsNAC061	1.42139	1.44057	2.98389	2.12973	1.30939	0.940351	0	0.573973	1.9148	1.69601	0.736745	0.931404667
SsNAC062	1.06739	0.962671	0.697656	1.27787	0.662631	0.425777	0.65521	0.311927	0.971716	0.689342	1.08877	0.689513333
SsNAC063	4.3267	4.35482	0.289942	0.876057	1.14907	2.5099	4.55722	0.472365	0.453598	0.391969	5.021403333	0.888028333
SsNAC064	0.515966	0.59146	0.476036	0.433554	0.555324	1.27413	0.108756	0.212	0.40151	0.0945824	2.77978	2.08461
SsNAC065	0	0	0.0961356	0	0	0	0	0	0	0	0.062308333	0.321375333
SsNAC066	0.079177	0	0.0513234	0	0	0	0	0	0	0	0.066219667	0.0281656
SsNAC067	0	0	0	0	0	0	0	0	0	0	0	0
SsNAC068	1.66317	0.101804	0.357657	13.4012	16.5299	0.999206	3.84338	0.421506	28.4025	43.8306	0.348169	0.274258167
SsNAC069	0	0	0	0	0	0	0	0	0	0	0	0
SsNAC070	0	0	0.101232	0	0	0	0	0.230544	0	0	0	0.044499667
SsNAC071	0	0	0.0223071	0	0	0	0	0	0	0	0	0
SsNAC072	0	0	0	0	0	0	0	0	0	0	0	0
SsNAC073	7.87559	12.9401	0.22432	1.28107	1.35261	0	1.9678	0.26584	0.887685	0.489093	1.305243333	0.625296333

SsNAC074	4.90838	6.938	0.0488147	1.51715	1.36586	0.570091	0.414001	0.202408	0.406666	0.159915	0.831842667	0.594057
SsNAC075	0	0	0	0	0.426947	0	0	0	0.317362	0.187858	0.2191741	0.074773
SsNAC076	0.698919	0.644014	0	0	0	0	0	0	0	0	0	0
SsNAC077	0	0	0	0	0	0	0	0	0	0	0.017131833	0
SsNAC078	10.9452	11.4834	0.403484	0.293189	0	6.20494	3.83176	0	0	0	0.500443	0
SsNAC079	0	0	0	0	0	0	0	0	0	0	0	0
SsNAC080	0	0	0	0	0	0	0	0	0	0	0	0
SsNAC081	0.131062	0.0734186	0	0.066502	0.13113	0	0	0	0	0	0	0
SsNAC082	0	0	0	0	0	0	0	0	0	0	0	0
SsNAC083	0	0	0	1.35485	3.5707	1.39316	0	0	4.9028	4.10186	0.080967667	0.169990667
SsNAC084	0	0.165628	0	0.029974	0.0591173	0	0	0	0	0	0	0
SsNAC085	0	0	0	0	0	0	0	0	0	0	0	0
SsNAC086	0	0	0	0	0	0	0	0	0	0	0	0
SsNAC087	0	0	0	0	0	0	0	0	0	0	0.180625667	0
SsNAC088	0.373521	0.236736	0.204971	1.05952	0.412674	0	0.475209	0.23395	1.14592	0.733198	1.07214	1.813531
SsNAC089	14.1837	13.4941	0.0859591	0.367466	0.144876	5.99972	5.64759	0	0.0515053	0.0811253	6.370933333	2.87249
SsNAC090	0.058541	0.0979803	0.480281	0.029834	0	0.124547	0.136005	0.268307	0.35412	0	0.106668967	0.873263333
SsNAC091	0.195525	0.193234	3.24643	4.23815	3.84409	0.548836	0.149563	1.27047	1.5645	1.57832	0.820468	5.791203333
SsNAC092	15.6474	14.6259	14.9016	5.89603	6.38834	6.90911	9.023	10.2241	3.9482	5.05536	7.5839	5.560106667
SsNAC093	41.8118	59.2528	4.29351	19.099	8.47658	22.5169	48.6224	0.747582	11.9778	13.9846	24.49823333	16.98633333
SsNAC094	90.6866	85.2989	90.0465	101.438	55.1041	68.5073	78.3783	114.699	114.766	104.231	99.28686667	72.6215
SsNAC095	0.028895	0.0484773	0.156816	0	0.115748	0	0	0.189219	0.12574	0	0.039283067	0.025908967
SsNAC096	0.050011	0.112199	0.0994546	0	0	0	0	0	0	0	0	0.022594233
SsNAC097	0	0.254504	0	0.410516	0.265498	0.304158	0	0	0	0	0.0311626	0.0376673
SsNAC098	0	0	0	0	0	0	0	0	0	0	0	0
SsNAC099	0.021116	0.0354919	1.1034	0.475626	0.675984	0.416627	0	0.933925	0.356388	0.28052	0.362110333	0.803355667
SsNAC100	0.207878	0.197343	0.254507	0.081019	0.0319726	0.123635	0.202006	0.197499	0.0881978	0.0520204	0.067164567	0.119314167
SsNAC101	0.19017	0.398831	0.509764	0.083796	0.0826661	0.224232	0.6281	0.102367	0.228499	0.134784	0.0778081	0.226967
SsNAC102	0	0	0	0	0	0	0	0	0	0	0	0
SsNAC103	0	0	0	0	0	0	0	0	0	0	0	0
SsNAC104	23.7641	26.6747	3.35929	6.98623	2.70421	10.4922	73.1474	3.52711	14.5996	11.4824	11.32536333	1.075648667
SsNAC105	2.23898	3.33672	0.0698195	0.694462	1.33262	1.2645	3.05807	0.30029	0.620739	0.157679	7.881156667	1.711326667
SsNAC106	1.18826	1.21342	1.26414	1.29527	0.682214	1.76192	1.54351	2.09343	2.8414	2.10473	1.430583333	1.78561
SsNAC107	0.2881	0.154979	0.420119	0.443884	0.430048	0.548801	0.06642	0.064933	0.848276	0.654249	0.714662333	0.8562
SsNAC108	0.116903	0.0830739	0.22876	0.406675	0.233731	0	0.065529	0.0640574	0.836935	0.392351	0.322402333	0.430227
SsNAC109	0.472303	0.203405	4.39246	13.5822	13.6901	0.29039	0.316576	1.86258	3.51741	7.08835	0.628533667	3.669176667
SsNAC110	0.139101	0	0.373273	0.623515	0.0557842	0	0	0.505224	0	0.395379	0	0.393845667
SsNAC111	0.344255	0.557808	1.20519	0.68066	0.588325	0.793225	0.246947	0.201431	0.592531	0.673932	1.235213	1.3109

SsNAC112	1.90042	2.33852	5.27216	4.49521	4.59243	1.64308	1.34253	1.75107	4.61421	2.76549	2.795316667	4.631243333
SsNAC113	0.017382	0.0778705	0.786052	0.723453	0.382664	0.276673	0.301589	0	0.197164	0	0.035768967	1.056236667
SsNAC114	0.491939	0.636138	0.808925	0.724581	0.563628	0.411106	0.524202	1.00479	1.57455	1.08158	0.902002333	0.865587667
SsNAC115	0	0	0	0	0	0	0	0	0	0	0	0
