# Supplementary material for: Genome-wide identification and expression analysis of the NAC transcription factor family in Saccharum spontaneum under different stresses
Source: Plant Signal Behav. 2022 Jun 22;17(1):2088665. doi: 10.1080/15592324.2022.2088665 (PMC9225438; doi:10.1080/15592324.2022.2088665)
Supplement: Supplemental Material [file KPSB_A_2088665_SM8717.zip › Supplementary Materials/Table S6.pdf]

Table S6. Drought stress for 7 days vs Control

GeneName	GeneID	Sspon-CK-RPKM	Sspon-RPKM	log2 Ratio(Sspon/Sspon-CK)	Up-Down-Regulation(gangeshoumi-2/geshoumi-2CK)	P-value	FDR	Description	GO Component	GO Function	GO Process
SsNAC004	Unigene0047094	12.468	15.9542	0.355705945	Up	0.38015	0.5242071	NAC domain-containing protein 78 [Aegilops tauschii]	-	GO:0003676 //nucleic acid binding	GO:0010468 //regulation of gene expression
SsNAC005	Unigene0075259	1.25769	22.7925	4.179716269	Up	9.76E-06	5.52E-05	NAC domain transcription factor [Zea mays]	-	GO:0003676 //nucleic acid binding	GO:0010468 //regulation of gene expression
SsNAC006	Unigene0040860	2.578	13.6048	2.399798529	Up	1.76E-06	1.13E-05	PREDICTED: NAC transcription factor 25-like [Setaria italica]	-	GO:0003676 //nucleic acid binding	GO:0010468 //regulation of gene expression
SsNAC007	Unigene0059193	0.7261	2.94756	2.021286906	Up	0.21242	0.3536673	NAC transcription factor [Zea mays]	-	-	-
SsNAC009	Unigene0043564	11.2173	4.21632	-1.411672501	Down	0.00585	0.0173757	PREDICTED: NAC domain-containing protein 21/22-like [Setaria italica]	-	GO:0003676 //nucleic acid binding	GO:0010468 //regulation of gene expression

SsNAC016	Unigene0074328	50.3647	367.854	2.868647564	Up	5.61E-13	8.01E-12	stress-induced NAC protein 1 [Sorghum bicolor]	-	GO:0003676 //nucleic acid binding	GO:0010468 //regulation of gene expression;GO:0009787//regulation of abscisic acid mediated signaling pathway;GO:0006950//response to stress
SsNAC019	Unigene0062491	0.001	0.4098	8.678778399	Up	0.06023	0.1303687	NAC transcription factor [Panicum virgatum]	-	GO:0003676 //nucleic acid binding	GO:0010468 //regulation of gene expression;GO:0009787//regulation of abscisic acid mediated signaling pathway;GO:0006950//response to stress

SsNAC020	Unigene0075347	0.59996	7.30651	3.606249407	Up	1.69E-05	9.09E-05	NAC transcription factor [Panicum virgatum]	-	GO:0003676 //nucleic acid binding	GO:0010468 //regulation of gene expression;GO:0009787//regulation of abscisic acid mediated signaling pathway;GO:0006950//response to stress
SsNAC022	Unigene0065760	0.59137	4.40115	2.895756024	Up	0.00015	0.0006397	NAC domain-containing protein 18 [Zea mays]	-	GO:0003676 //nucleic acid binding	GO:0010468 //regulation of gene expression

SsNAC028	Unigene0043205	56.4441	243.453	2.108749747	Up	3.04E-14	6.30E-13	stress-induced NAC protein 1 [Sorghum bicolor]	-	GO:0003676 //nucleic acid binding	GO:0010468 //regulation of gene expression;GO:0009787//regulation of abscisic acid mediated signaling pathway;GO:0006950//response to stress
SsNAC029	Unigene0066616	1.20555	0.97878	-0.300641189	Down	0.67117	0.7466259	PREDICTED: NAC transcription factor NAM-1-like [Setaria italica]	-	-	-
SsNAC030	Unigene0071947	3.96526	18.3808	2.212712977	Up	2.20E-13	3.52E-12	PREDICTED: NAC domain-containing protein 21/22-like [Setaria italica]	-	-	-
SsNAC035	Unigene0012898	3.82677	4.58977	0.262295006	Up	0.66281	0.7381215	PREDICTED: NAC transcription factor ONAC010-like isoform X1 [Setaria italica]	-	-	-

SsNAC036	Unigene0028174	9.15352	9.28958	0.021286906	Up	0.94625	0.9919027	NAC transcription factor [Zea mays]	-	GO:0003676 //nucleic acid binding	GO:0010468 //regulation of gene expression
SsNAC037	Unigene0043204	18.8046	133.169	2.824105048	Up	0	0	NAC transcription factor [Zea mays]	-	GO:0003676 //nucleic acid binding	GO:0010468 //regulation of gene expression
SsNAC045	Unigene0034531	30.235	32.4894	0.103749066	Up	0.41037	0.5566713	ANAC075 [Zea mays]	-	-	-
SsNAC046	Unigene0040862	0.001	4.77745	12.22202588	Up	0.00365	0.011406	PREDICTED: NAC transcription factor 25-like [Setaria italica]	-	GO:0003676 //nucleic acid binding	GO:0010468 //regulation of gene expression
SsNAC051	Unigene0071857	0.98261	1.35984	0.468745883	Up	0.41948	0.5681882	PREDICTED: NAC domain-containing protein 67-like [Setaria italica]	-	-	-
SsNAC052	Unigene0067555	44.1758	54.8372	0.311897556	Up	0.00046	0.0017854	NAC domain-containing protein 78 [Zea mays]	-	-	-

SsNAC056	Unigene0050544	10.0768	5.53937	-0.863235876	Down	0.08052	0.1607608	NAC domain-containing protein 43 [Aegilops tauschii]	GO:0043231//intracellular membrane-bounded organelle	GO:0003677//DNA binding	GO:0006355//regulation of transcription, DNA-dependent;GO:0009699//phenylpropanoid biosynthetic process;GO:0009900;GO:0009832//plant-type cell wall biogenesis
SsNAC057	Unigene0008169	0.001	3.01473	11.55781134	Up	0.00736	0.0210135	NAC13 NAC type transcription factor, partial [Zea mays subsp. mays]	GO:0043231//intracellular membrane-bounded organelle	GO:0003676//nucleic acid binding	GO:0010468//regulation of gene expression
SsNAC070	Unigene0067324	0.001	0.39277	8.61753121	Up	0.49265	0.6455123	NAC transcription factor [Hordeum vulgare subsp. vulgare]	-	-	-
SsNAC071	Unigene0009062	1.30548	5.96197	2.191211908	Up	0.00237	0.0076576	NAC domain-containing protein 21/22 [Zea mays]	-	GO:0003676//nucleic acid binding	GO:0010468//regulation of gene expression

SsNAC076	Unigene0050514	1.7752	5.70504	1.684251919	Up	0.00841	0.0235201	PREDICTED: NAC domain-containing protein 77-like [Setaria italica]	-	-	-
SsNAC077	Unigene0030713	2.19237	12.9047	2.557339806	Up	4.72E-05	0.000232	PREDICTED: NAC domain-containing protein 68-like [Setaria italica]	-	GO:0003676 //nucleic acid binding	GO:0010468 //regulation of gene expression
SsNAC078	Unigene0075326	22.071	26.3461	0.255434351	Up	0.04968	0.1102204	PREDICTED: NAC transcription factor NAM-1-like [Setaria italica]	-	-	-
SsNAC082	Unigene0073206	24.0552	34.0573	0.501615866	Up	0.01645	0.0422912	PREDICTED: NAC domain-containing protein 71-like isoform X2 [Setaria italica]	-	GO:0003676 //nucleic acid binding	GO:0010468 //regulation of gene expression
SsNAC086	Unigene0043032	0.72986	0.74071	0.021286906	Up	0.97538	1.0156464	NAC domain-containing protein 68 [Zea mays]	-	GO:0003676 //nucleic acid binding	GO:0010468 //regulation of gene expression
SsNAC087	Unigene0043561	6.54087	2.15738	-1.600201471	Down	0.00022	0.0008938	NAC domain-containing protein 21/22 [Zea mays]	-	-	-

SsNAC089	Unigene0043560	0.85871	1.01672	0.243679328	Up	0.76899	0.8251911	NAC transcription factor [Zea mays]	-	GO:0003676 //nucleic acid binding	GO:0010468 //regulation of gene expression
SsNAC091	Unigene0062683	4.30133	1.87083	-1.201105515	Down	0.00541	0.0161113	secondary wall NAC transcription factor 4 [Zea mays]	-	GO:0003676 //nucleic acid binding	GO:0009725 //response to hormone stimulus;GO:0010468//regulation of gene expression
SsNAC092	Unigene0050545	4.26368	2.70441	-0.656784999	Down	0.2639	0.3869446	secondary wall NAC transcription factor 2 [Zea mays]	GO:0043231//intracellular membrane-bounded organelle	GO:0003677 //DNA binding	GO:0006355 //regulation of transcription, DNA-dependent;GO:0009699//phenylpropanoid biosynthetic process;GO:0009900;GO:0009832//plant-type cell wall biogenesis
SsNAC093	Unigene0052694	9.71098	10.3481	0.091676234	Up	0.83985	0.8883097	NAC domain-containing protein 78 [Zea mays]	-	-	-

SsNAC095	Unigene0075084	42.7827	104.748	1.291827847	Up	0	0	PREDICTED: NAC domain-containing protein 55-like [Setaria italica]	-	GO:0003676 //nucleic acid binding	GO:0010468 //regulation of gene expression
SsNAC099	Unigene0066719	0.10595	0.8602	3.021286906	Up	0.02079	0.0524891	PREDICTED: NAC transcription factor NAM-1-like [Setaria italica]	-	-	-
SsNAC100	Unigene0004366	1.37643	0.001	-10.42671129	Down	0.25557	0.3769004	NAC domain-containing protein 78 [Aegilops tauschii]	-	GO:0003676 //nucleic acid binding	GO:0010468 //regulation of gene expression
SsNAC104	Unigene0050543	8.70475	9.41533	0.113209396	Up	0.6238	0.7071196	PREDICTED: NAC transcription factor ONAC010-like [Setaria italica]	-	-	-
SsNAC105	Unigene0043565	2.76315	2.80422	0.021286906	Up	0.97683	1.0162027	PREDICTED: NAC domain-containing protein 21/22-like [Setaria italica]	-	GO:0003676 //nucleic acid binding	GO:0010468 //regulation of gene expression

SsNAC107	Unigene0062815	6.7119	13.9072	1.05103425	Up	0.00275	0.0087891	PREDICTED: NAC domain-containing protein 8-like [Setaria italica]	-	GO:0003676//nucleic acid binding	GO:0010564//regulation of cell cycle process;GO:0010212//response to ionizing radiation;GO:0010468//regulation of gene expression;GO:0006974//response to DNA damage stimulus

SsNAC108	Unigene0077954	0.45225	2.29488	2.343215001	Up	0.12077	0.2287898	PREDICTED: NAC domain-containing protein 8-like [Setaria italica]	-	GO:0003676//nucleic acid binding	GO:0010564//regulation of cell cycle process;GO:0010212//response to ionizing radiation;GO:0010468//regulation of gene expression;GO:0006974//response to DNA damage stimulus
SsNAC109	Unigene0053402	59.5968	37.7412	-0.65909516	Down	0.00138	0.0047378	PREDICTED: NAC domain-containing protein 8-like [Setaria italica]	-	-	GO:0050896//response to stimulus;GO:0010564//regulation of cell cycle process
SsNAC110	Unigene0053403	4.55726	13.4125	1.557339806	Up	5.52E-08	4.47E-07	PREDICTED: NAC domain-containing protein 8-like [Setaria italica]	-	-	-

SsNAC111	Unigene0021645	3.6284	39.5851	3.447551661	Up	4.51E-08	3.73E-07	PREDICTED: NAC domain-containing protein 43-like [Setaria italica]	-	GO:0003676 //nucleic acid binding	GO:0010468 //regulation of gene expression
SsNAC113	Unigene0053401	43.6309	36.7279	-0.248473363	Down	0.18818	0.3191898	PREDICTED: NAC domain-containing protein 8-like [Setaria italica]	-	-	GO:0050896 //response to stimulus;GO:0010564//regulation of cell cycle process
SsNAC114	Unigene0049308	1.32077	2.37148	0.844409144	Up	0.09003	0.1778536	PREDICTED: NAC domain-containing protein 77-like [Brachypodium distachyon]	-	GO:0003676 //nucleic acid binding	GO:0010468 //regulation of gene expression
