# Supplementary material for: Genome-wide identification and expression analysis of the NAC transcription factor family in Saccharum spontaneum under different stresses
Source: Plant Signal Behav. 2022 Jun 22;17(1):2088665. doi: 10.1080/15592324.2022.2088665 (PMC9225438; doi:10.1080/15592324.2022.2088665)
Supplement: Supplemental Material [file Figure S1.pdf › Supplementary Materials/Figure S1.pdf]

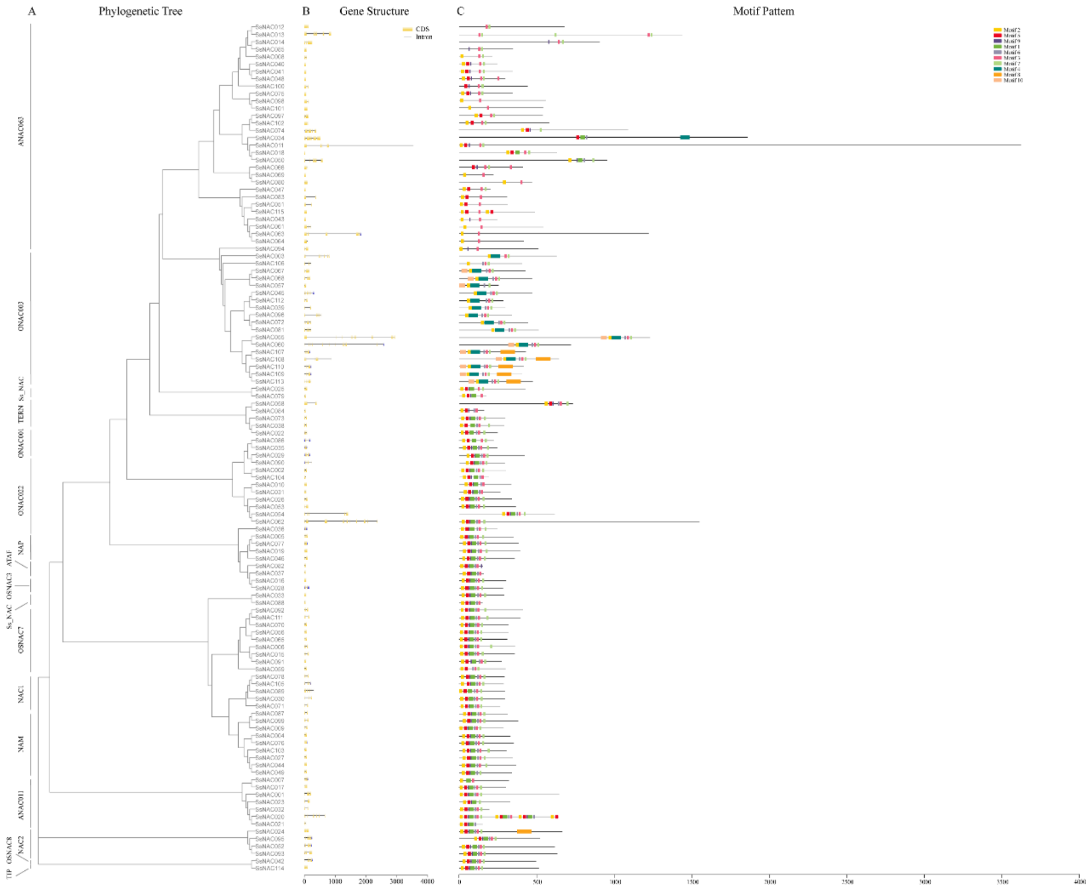

Figure S1. Phylogenetic relationships, gene structures, and motif structures of conserved protein motifs in *SsNAC* proteins. a. Using MEGA-X software, a phylogenetic tree was constructed based on conserved sequences in *SsNAC* proteins. b. The structure of *SsNAC* genes. The yellow box indicates an exon, and the black line indicates an intron. c. The motif composition of *SsNAC* proteins. Motifs 1-10 are represented by boxes with different colors. The sequence information for each motif is provided in Table S4.
